# Elucidation of the mechanism of berberine against gastric mucosa injury in a rat model with chronic atrophic gastritis based on a combined strategy of multi-omics and molecular biology

**DOI:** 10.3389/fphar.2024.1499753

**Published:** 2025-01-06

**Authors:** Lisheng Chen, Xin Wang, Jianyu Li, Lijuan Zhang, Wenbin Wu, Shizhang Wei, Wenjun Zou, Yanling Zhao

**Affiliations:** ^1^ College of Pharmacy, Chengdu University of Traditional Chinese Medicine, Chengdu, China; ^2^ Department of Pharmacy, The Fifth Medical Center of Chinese PLA General Hospital, Beijing, China; ^3^ Healthcare Office of the Service Bureau of Agency for Offices Administration of the Central Military Commission, Beijing, China; ^4^ National Cancer Center/National Clinical Research Center for Cancer/Cancer Hospital, Chinese Academy of Medical Sciences and Peking Union Medical College, Beijing, China

**Keywords:** berberine, chronic atrophic gastritis, widely targeted metabolomics, transcriptomics, 16S rRNA sequencing

## Abstract

**Background:**

Berberine (BBR) is widely used to treat gastrointestinal diseases. However, the pharmacological mechanism of action of BBR in anti-chronic atrophic gastritis (CAG) remains unclear. This study aimed to investigate the mechanism of action of BBR in CAG by integration of molecular biology and multi-omics studies strategy.

**Methods:**

The CAG model was established by alternating drinking water of 0.1% ammonia and 20 mmol/L sodium deoxycholate, accompanied by an irregular diet. Serum biochemical indices including PGI, PGII, GAS-17, IL-6, IL-1β, and TNF-α were analyzed. HE and AB-PAS staining were employed to assess pathological damage in gastric tissue. The underlying molecular mechanism of BBR in CAG treatment was explored via the integration of network pharmacology, transcriptomics, widely targeted metabolomics and intestinal flora analysis. Finally, relevant key targets and pathway were verified.

**Results:**

The results showed that BBR exerted therapeutic effects in improving CAG via alleviating inflammation response, maintaining the gastric mucosal barrier’s integrity and repairing gastric mucosal tissues. Network pharmacology showed that the treatment of CAG by BBR mainly involved in inflammatory response, apoptosis, angiogenesis and metabolic processes. Furthermore, 234 different expression genes were identified in the gastric tissue transcriptome, which were mainly involved in biological processes such as cell adhesion, angiogenesis, apoptosis, cell migration and lipids metabolism by regulating the MAPK signaling pathway. Metabolomics results showed that 125 differential metabolites were also identified, while the pathways were mainly involved in D-glutamine and D-glutamate metabolism, and tyrosine metabolism, etc. Integrating transcriptomics and metabolomics analyses indicated that BBR directly regulated Carnitine C3:0, LPC (0:0/20:3), L-Glutamic Acid and FFA (15:0) by acting on SLC25A20, PNLIPRP1, PLA2G4C, GSR, GFPT2, GCLM, CTPS1, ACSL1, ACOT4 and ACOT2. 16S rRNA sequencing revealed that BBR could restore the balance of gut microbiota dysbiosis by significantly regulating the relative abundance of *unclassified_Muribaculaceae* and *Lactobacillus_johnsonii*.

**Conclusion:**

This study demonstrated that BBR alleviates CAG through the regulation of the MAPK signaling pathway, metabolic disorders and gut microbiota dysbiosis, thereby revealing the complex mechanism of BBR in relation to alleviating CAG from multiple levels and perspectives.

## 1 Introduction

Chronic atrophic gastritis (CAG) is a common digestive tract disease characterized by atrophy of the gastric mucosal epithelium and glands, mucus thinning, decrease in glands, and thickening of the mucosa and muscle (Botezatu and Bodrug, 2021). The typical clinical symptoms of patients with CAG include upper abdominal pain, indigestion, bloating, nausea, vomiting, belching, appetite loss, and weight loss (Zhou et al., 2022). As reported, the incidence of CAG is increasing owing to various factors such as emotional stress, physicochemical factors, and microbial infection. Most importantly, patients with CAG have an increased risk of GC, with an estimated annual risk of GC being 0.1% year (Yin et al., 2021). Nevertheless, no specific medicine has been effectively employed to treat CAG because of its complicated etiology and pathophysiology (Liu et al., 2023a). Therefore, the development of new and effective treatments for CAG is critical.

Berberine (BBR) is an isoquinoline alkaloid mainly derived from *Coptis chinensis* that has been widely applied in clinical therapy for many centuries. Mounting evidence suggests that BBR possesses multiple pharmacological effects, including anti-inflammatory, anti-tumor, bacteriostatic, and antidiabetic effects (Ayati et al., 2017; Sharifi-Rad et al., 2021; Wang et al., 2017). BBR has been widely studied owing to its excellent biological activities, and an increasing number of studies have been conducted on its further development. There are increasing reports elucidated BBR exerts potential gastroprotective effects (Tong et al., 2021a; Yang et al., 2020). However, the mechanism by which BBR treats CAG has not been completely elucidated.

In recent years, multi-omics research focusing on quantitative and high-throughput screening has developed rapidly, providing a new direction for discovering the basis of biochemical substances and understanding their molecular mechanism, thus providing new ideas and methods in research of traditional Chinese medicine (Yan et al., 2015). Metabolomics is a relatively new approach for revealing the metabolic trends and laws of the whole system under the influence of internal and external factors that reflect a series of biological changes that occur during certain pathological and physiological processes (Alarcon-Barrera et al., 2022). Transcriptomics is a potent approach for elucidating tissue properties and various physiological functions by sensitively detecting small changes in mRNA expression (Jian et al., 2020). Gut microbiota studies provide a detailed analysis of the composition of the microbes in the gut, which helps to determine the diversity, abundance, and function of the flora. Genes are associated with metabolites and gut microbiota; therefore, the combination of gut microbiota, transcriptomics, and metabolomics can reveal metabolic pathways and gene functions associated with phenotypes by identifying metabolites, differential microbiota, and differential genes, further obtaining potential therapeutic targets for CAG and clarify the mechanism of BBR therapy for CAG.

**FIGURE 1:**
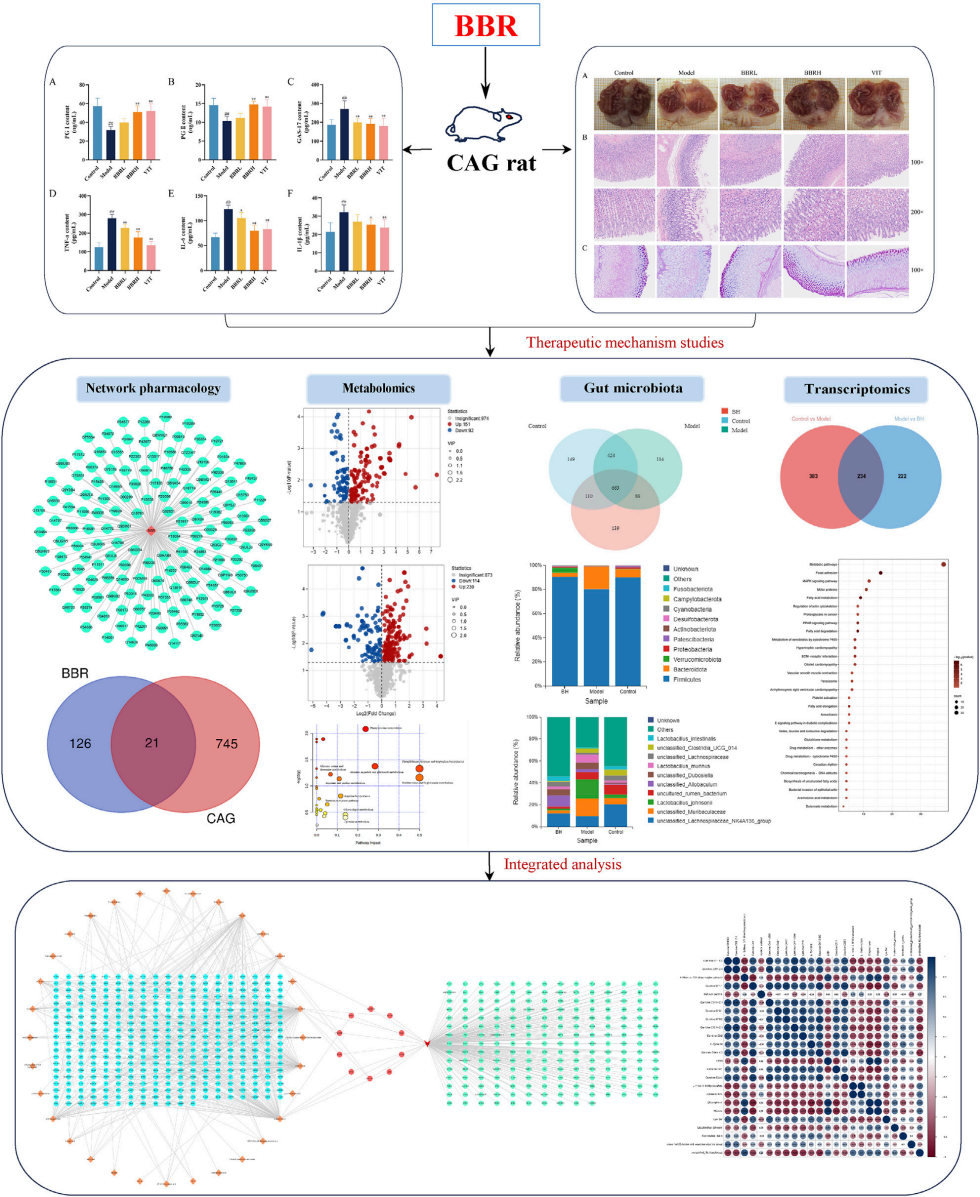
Flow chart of this study.

The aim of the present study was to investigate the therapeutic effect of BBR on CAG and preliminarily investigate the detailed mechanism of BBR in improving CAG based on integrative gut microbiota, transcriptomics and metabolomics, which might lay the foundation for further investigation of its protective mechanism against CAG (Figure 1).

## 2 Materials and methods

### 2.1 Chemicals and reagents

BBR (purity ≥98%, Lot: CHB210108) was supplied by Chengdu Keloma Biotechnology Co., Ltd. Sodium deoxycholate (Lot: CD33141310) and ammonia (Lot: 2023AS0328) were respectively supplied by Beijing Zhongke Ruijin Technology Co., Ltd. and Biotechnology Co., Ltd. All antibodies and reagents were obtained from commercial sources.

### 2.2 Animal experiments

Male Sprague-Dawley rats weighing 200 ± 20 g were purchased from Sibeifu Biotechnology Co., Ltd. [Beijing, China; license No: SCXK (Beijing) 2019-0010]. This study was approved by the Institutional Animal Ethics Committee (approval ID: IACUC-2021-0022). The rats were acclimatized for 1 week and then randomly divided into five groups: control, model, low-dose BBR (BBRL, 14 mg/kg), high-dose BBR (BBRH, 28 mg/kg), positive drug (Vitacoenzyme, VIT, 200 mg/kg) (Tong et al., 2021a). The establishment of the CAG model was based on published literature (Cui et al., 2017). The details were as follows: Except for the control group, which was normally fed, all other groups were provided with 20 mmol/L sodium deoxycholate solution and 0.1% ammonia water solution alternately as drinking water for free consumption, along with an irregular diet. The model was established continuously for 10 weeks, and two rats were randomly selected from each group to test whether the CAG model was successful. After the success of the CAG model, each administration group was given the corresponding drug according to the dose of 1 mL/100 g by intragastric administration for 28 days.

### 2.3 Sample collection

After 28 days of continuous administration, rats were fasted with water for 48 h. Next, blood, gastric tissue, cecal contents, and gastric juice samples were collected. Part of the gastric tissue was fixed in 10% paraformaldehyde for histopathological analysis, the other part was stored at −80°C for subsequent research.

### 2.4 Measurement of gastric PH

The pH value of the centrifuged gastric juice was measured using precision pH indicator paper. Afterward, the same individual used a colorimetric chart to read the pH for qualitative analysis.

### 2.5 Enzyme-linked immunosorbent assay (ELISA)

The serum levels of Pepsinogen I (PG I), Pepsinogen II (PG II), Gastrin17 (GAS-17), interleukin-6 (IL-6), interleukin-1β (IL-1β), and tumor necrosis factor-α (TNF-α) in the serum were measured by ELISA kits. All kits were provided by Shanghai Enzyme-Linked Biotechnology Co., Ltd. The kit information was shown in Supplementary Table 1.

### 2.6 Histological staining

The gastric tissues were fixed with 10% formaldehyde solution for 48 h, and embedded in paraffin. Then, 4 µm sections were used for hematoxylin and eosin (H&E) and Alcian blue-periodic acid-Schiff (AB-PAS) staining. Histopathological images of the gastric tissues were obtained using Nikon microscope (Nikon Instruments, Tokyo, Japan).

### 2.7 Network pharmacology

#### 2.7.1 Acquisition of BBR targets and CAG targets

The targets of the candidate components of BBR was screened in the SwissTargetPrediction database (http://www.swisstargetprediction.ch/) and PharmMapper database (http://www.lilab-ecust.cn/pharmmapper/). The CAG targets were retrieved from the GeneCards (http://www.genecards.org/) and Online Mendelian Inheritance in Man (OMIM, http://www.omim.org/) databases with “chronic atrophic gastritis” or “CAG” as the search term. After integrating and deduplicating, the Cytoscape 3.8.0 software was used to visualize the network graph.

#### 2.7.2 Screening and network construction of intersecting targets

A Venn diagram of the CAG and ZJP targets was created using the online Venn tool (https://bioinformatics.psb.ugent.be/webtools/Venn/) and the intersection was taken.

#### 2.7.3 GO and KEGG pathway enrichment analysis

Kyoto Encyclopedia of Genes and Genomes (KEGG) pathway enrichment analysis and Gene Ontology (GO) analysis were conducted by associating targets with the Database for Annotation, Visualization and Integrated Discovery. Finally, the top 30 pathways in KEGG and the top 10 pathways in cell composition, biological process, and molecular function in GO enrichment analysis were selected for visual analysis.

### 2.8 Transcriptomic analysis of gastric tissue

Total RNA was extracted from gastric tissue according to the instruction manual of the TRIzol Reagent. RNA concentration and purity were measured using a NanoDrop 2000 spectrophotometer (Thermo Fisher Scientific). RNA integrity was accurately determined. A cDNA library was constructed and library quality was assessed using the Agilent Bioanalyzer 2100 system (Agilent Technologies, Inc.). The Illumina NovaSeq platform was used for sequencing. The raw reads were further processed using the bioinformatics pipeline, BMKCloud (www.biocloud.net). Significantly differentially expressed genes (DEGs) were screened by DESeq2 based on a *P*-value <0.01 and a Fold Chang ≥2.

### 2.9 Widely targeted metabolomics analysis of gastric tissue

Gastric tissue stored at −80°C refrigerator was thawed on ice and ground with liquid nitrogen. A 400 μL solution (methanol: water = 7:3, v/v) containing the internal standard was added to 20 mg of ground gastric tissue and shaken at 2,500 rpm for 5 min. After placing the sample on ice for 15 min, it was centrifuged at 12,000 rpm and 4°C for 10 min. The supernatant was incubated for 30 min at −20°C and centrifuged for 3 min at 12,000 rpm at 4°C. The supernatant (200 μL) was collected for further analysis.

Metabolomic profiling of gastric tissue was performed using an LC-ESI-MS/MS system (UPLC, ExionLC AD, https://sciex.com.cn/; MS, QTRAP^®^ System, https://sciex.com/). The column temperature was set at 40°C and the injection volume was 2 μL. The mobile phase was water (0.1% formic acid) and acetonitrile (0.1% formic acid) with the following gradient program: 95:5 V/V at 0 min, 10:90 V/V at 11.0 min, 10:90 V/V at 12.0 min, 95:5 V/V at 12.1 min, 95:5 V/V at 14.0 min. The flow rate was set at 0.40 mL/min. The ESI source operating parameters were as follows: source temperature, 500°C; ion spray voltage (IS), 5,500 V (positive), −4,500 V (negative); ion source gas I (GSI), gas II (GSII), and curtain gas (CUR) were set at 55, 60, and 25.0 psi, respectively; and the collision gas (CAD) was high.

Differential metabolites were determined using VIP (VIP > 1) and *P*-value (*P*-value <0.05, Student’s t-test). MetaboAnalyst 5.0 (http://www.metaboanalyst.ca/) was employed to identify potential metabolic pathways.

### 2.10 Integrated analysis of metabolomics and transcriptomic

To further investigate the potential mechanisms of BBR regulation of differential metabolites, we conducted a comprehensive analysis combining transcriptomics and metabolomics. The targets of the metabolites were obtained from the Metabolites Biological Role database (MBrole: http://cs.bg.cnb.csic.es/mbrole2/). The “BBR-target-metabolite” interaction network was constructed by Cytoscape3.8.0.

### 2.11 16S rRNA gene sequencing analysis

The bacterial genome was extracted from cecal content of rat and DNA concentration was determined. PCR amplification, purification and quantification were performed. Then the PacBioSequelII platform (Beijing Biomarker Technology Co., LTD., Beijing, China) was used for sequencing. The sequencing data were analyzed using the online platform BMKCloud (https://www.biocloud.net). To assess the complexity of the species, alpha and beta diversity were performed. One-way analysis of variance was used to compare bacterial abundance and diversity. Linear discriminant analysis (LDA) and effect size (LEfSe) were used for the evaluation of taxa with differential abundance.

### 2.12 Total RNA extraction and real-time PCR

Total RNA was extracted from rat gastric tissues using a total RNA extraction kit. cDNA was synthesized using a cDNA Reverse Transcription Kit. RT-qPCR was performed using the SYBR Green PCR Master Mix on a 7500 Fast Real-Time PCR System (Applied Biosystems, Foster City, CA, United States). GAPDH was used as an internal control and the relative transcription levels of the target genes were calculated using the 2^−△△Ct^ method. The primer sequences used were listed in Supplementary Table 2.

### 2.13 Western blot analysis

The total protein of gastric tissue was extracted using ice-cold radioimmunoprecipitation assay (RIPA) buffer, which included phenylmethylsulfonyl fluoride (PMSF) and a phosphatase inhibitor. Protein concentrations were then quantified using a BCA protein assay kit. Protein samples were subjected to sodium dodecyl sulfate-polyacrylamide gel electrophoresis (SDS-PAGE) and subsequently transferred to polyvinylidene difluoride (PVDF) membranes. Following this, the membranes were blocked at room temperature for 2 h. Then, the corresponding primary antibody was incubated overnight at 4°C with primary antibodies respectively. All membranes were washed three times with TBST, which consists of TBS supplemented with 0.1% Tween 20. Subsequently, the membranes were incubated with HRP-conjugated secondary antibody. Finally, the light microscope (Olympus, Japan) was applied to obtain the images. Quantitative analysis of the bands was performed using ImageJ software (National Institutes of Health, Bethesda, United States). GAPDH served as an internal control. The information of the antibodies used was shown in Supplementary Table 3.

### 2.14 Immunohistochemical (IHC) staining

Gastric tissues were routinely dehydrated, embedded, sectioned, dewaxed, and antigen retrieved. Primary antibodies against ZO-1 (1:100), occludin (1:300), E-cadherin (1:300) and Claudin-4 (1:100) were added and incubated overnight in a moist box at 4°C. Next, the sections were incubated with horseradish peroxidase (HRP)-conjugated goat anti-rabbit secondary antibody and counterstained with hematoxylin. The sections were monitored with a light microscope (Olympus, Japan) at ×40 magnification, and images were captured in random fields of vision. The expression of ZO-1, occludin, E-cadherin and Claudin-4 was evaluated with IOD using ImageJ software (National Institutes of Health, Bethesda, MD, United States).

### 2.15 Statistical analysis

Statistical analysis of the data was performed using GraphPad Prism 8.0, and the results were expressed as mean ± standard deviation (SD). Statistical differences between groups were analyzed with a two-tailed Student’s t-test or one-way analysis of variance (ANOVA). *P* < 0.05 was considered statistically significant.

## 3 Results

### 3.1 Evaluation of CAG model establishment

**FIGURE 2:**
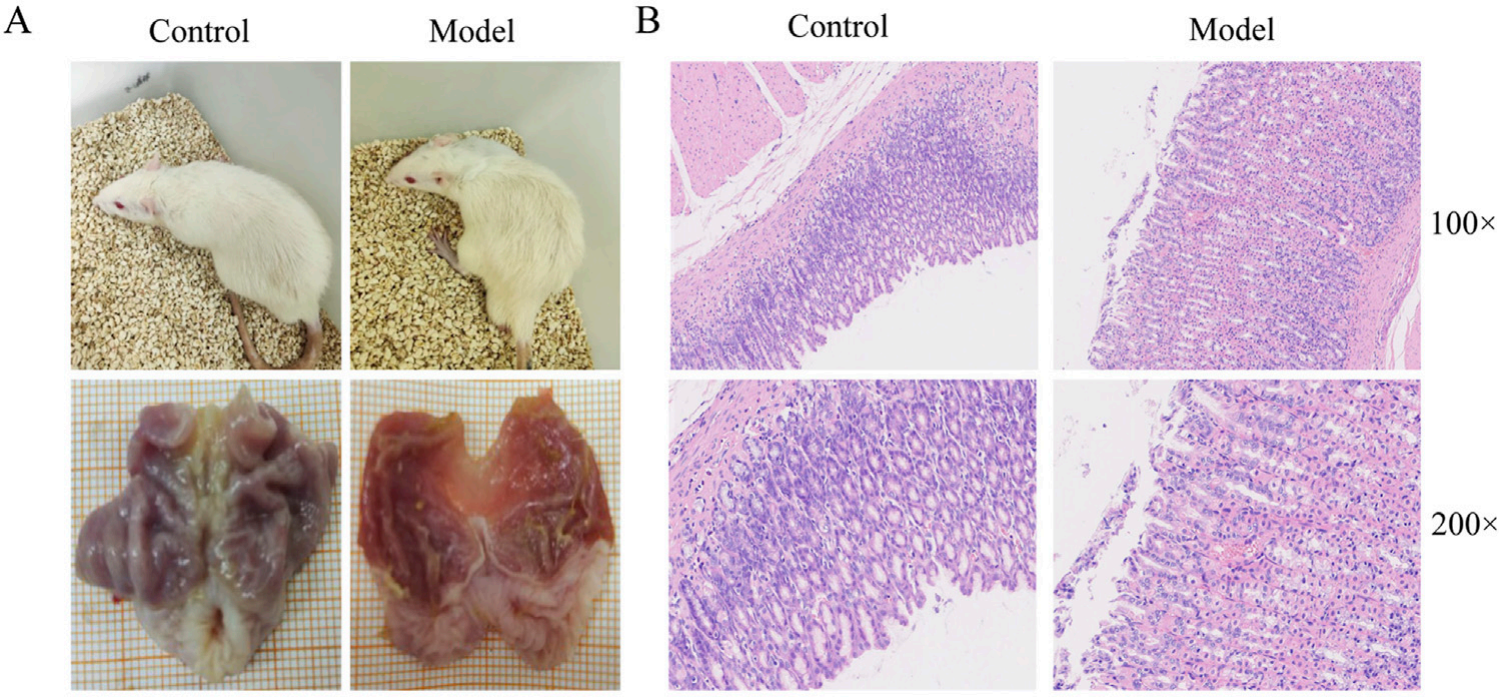
Evaluation of CAG model establishment. **(A)** The general state of rats and macroscopic manifestation of gastric tissue. **(B)** Gastric tissue pathological changes of rats in control and model group.

To verify the success of the model, we observed the general condition, macroscopic and pathologic appearance of rat gastric tissues. The results showed that the rats in the CAG model group were lethargic, had decreased activity, disheveled fur, and lacked luster. Compared with the control group, the CAG model group had thinner stomach lining, fewer and flatter lining folds, and less elastic tissue (Figure 2A). HE staining showed intrinsic gland loss, partial mucosal detachment, and neutrophil infiltration in the CAG model group, indicating successful model establishment (Figure 2B).

### 3.2 BBR improved the general signs of rats with CAG

**FIGURE 3:**
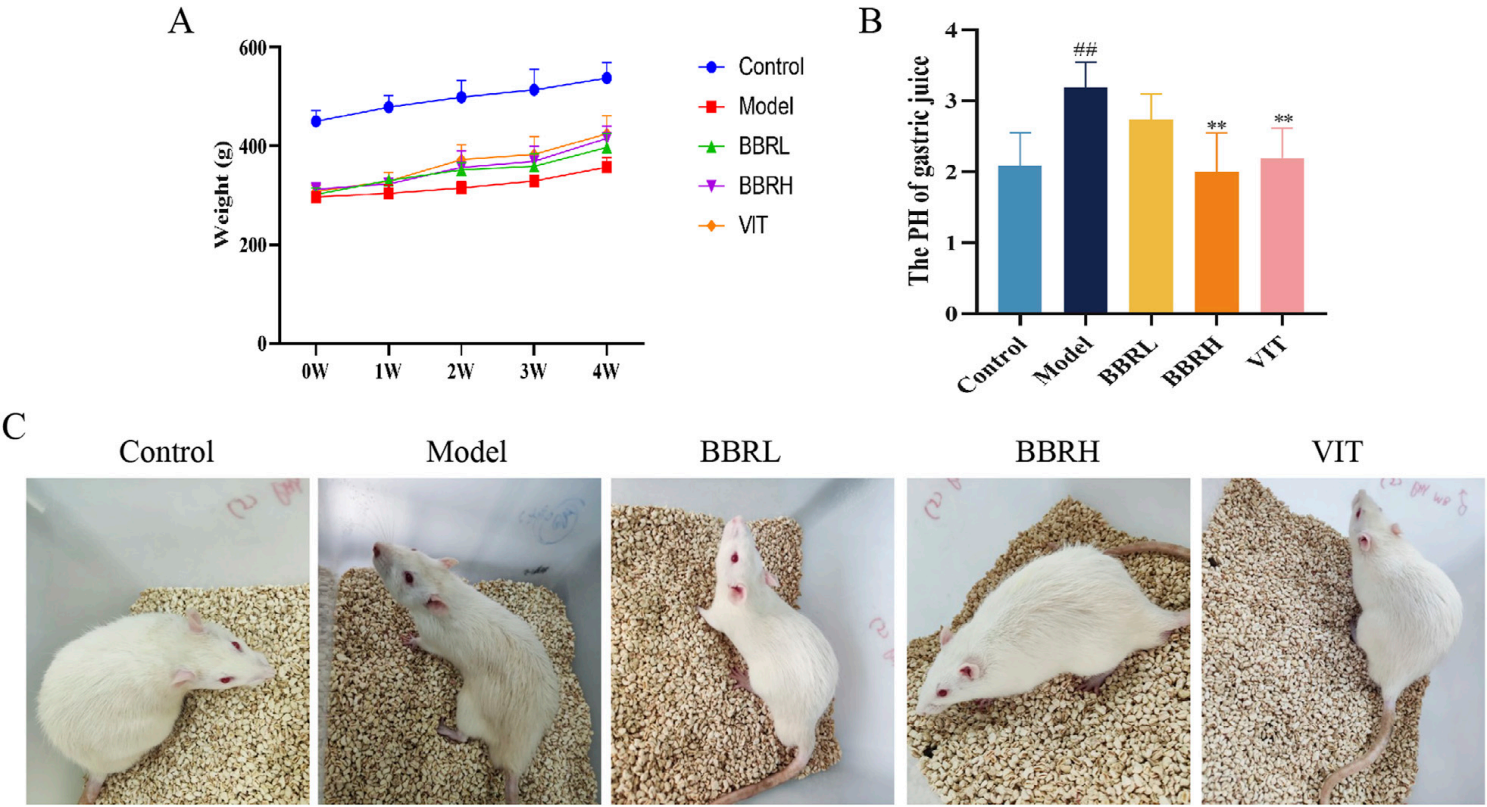
Therapeutic effects of BBR on CAG rats. **(A)** The curve of rat body weight changes. **(B)** The result of gastric juice PH. **(C)** Status of rats in each group. All data were expressed as mean ± SD. ^##^ *P* < 0.01 vs. the control group. **P < 0.01 vs. the model group (n = 6).

The body weight of rats in the control group gradually increased with dietary time. In contrast, the weight gain of rats in the CAG group was slower, and by the end of the experiment, the body weight of rats in the model group was significantly lower compared to that of the rats in the control group. The BBR and positive drug groups showed significant improvement in body weight (Figure 3A). The pH value of gastric juice in the model group was significantly higher compared to the control group. However, after administration of BBR, the pH value of gastric juice decreased to varying degrees, indicating that BBR had an excellent moderating effect on gastric juice pH (Figure 3B). Additionally, CAG rats showed symptoms such as yellowish fur, low energy, lethargy, and decreased appetite. All these symptoms were alleviated to some extent following treatment with BBR and VIT (Figure 3C).

### 3.3 BBR improved histopathological changes of gastric tissue in CAG rats

**FIGURE 4:**
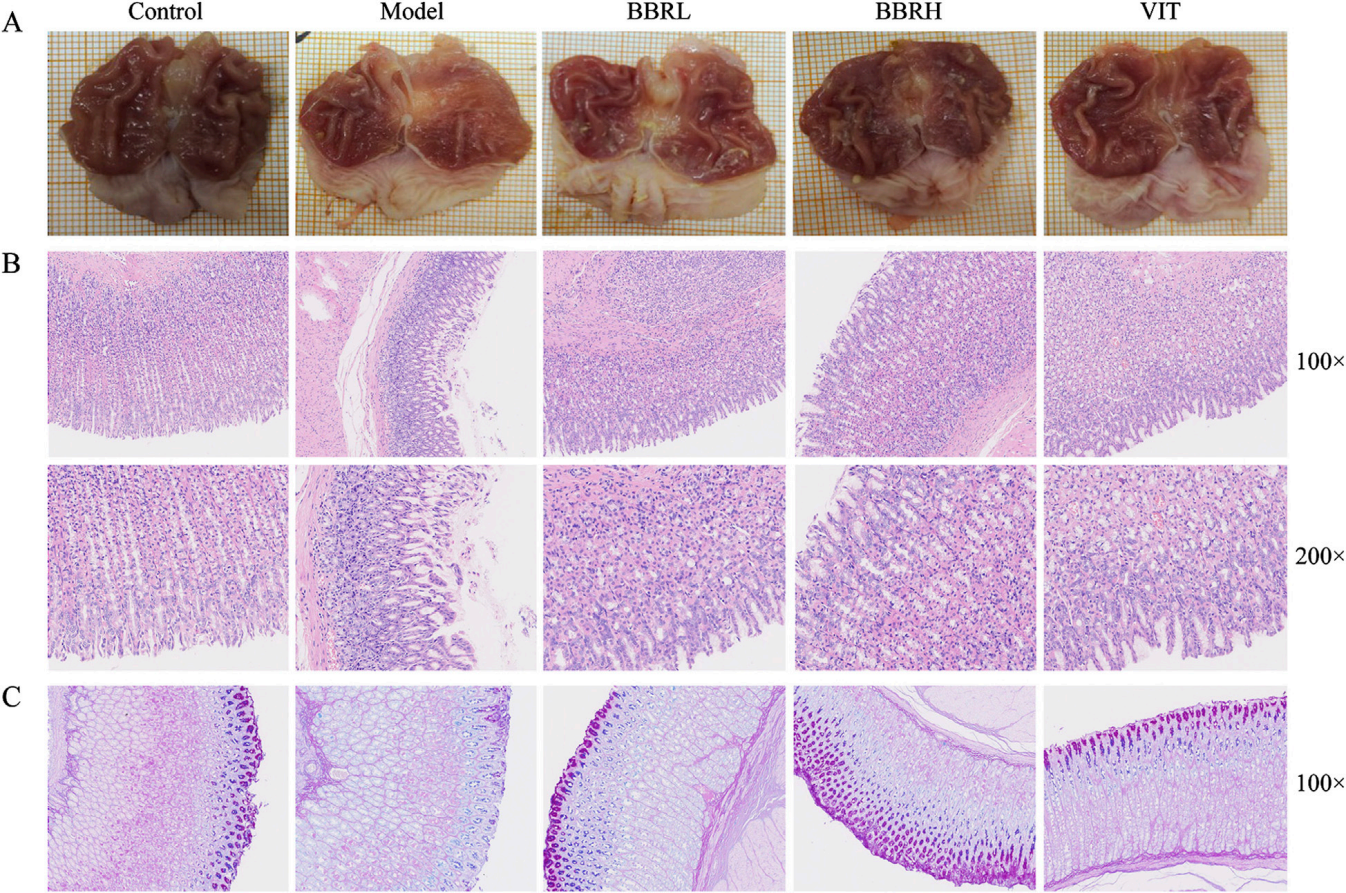
BBR alleviated gastric mucosal injury in CAG rats. **(A)** The macroscopical manifestation of gastric tissue in rats. **(B)** Histological examinations of gastric tissues (HE staining, ×100, ×200). **(C)** The result of AB-PAS staining.

To assess the potential role of BBR in CAG, both macroscopic and microscopic analyses were conducted to examine the overall morphology and pathological changes in the gastric tissues. Compared with the control group, the gastric tissue in the model group exhibited thinning and shallower wrinkles. In contrast, treatment with BBR notably improved both the thinning of the gastric tissue and the reduction in wrinkles in CAG rats (Figure 4A). HE staining indicated that the gastric mucosal epithelial cells were exfoliated, the number of glands was decreased, and inflammatory cells infiltrated the model group. Gastric mucosal injury in rats improved to a certain extent after BBR and VIT (Figure 4B). Additionally, the thickness of the functional gastric mucosa was assessed using AB-PAS staining. The results revealed that the positive staining layer was significantly thinner in the model group, whereas it was restored to varying degrees in the BBR and VIT groups. This suggests that BBR and VIT had a protective effect on the mucosal barrier (Figure 4C).

### 3.4 BBR inhibited inflammatory injury and maintained the balance of blood biomarkers of CAG

**FIGURE 5:**
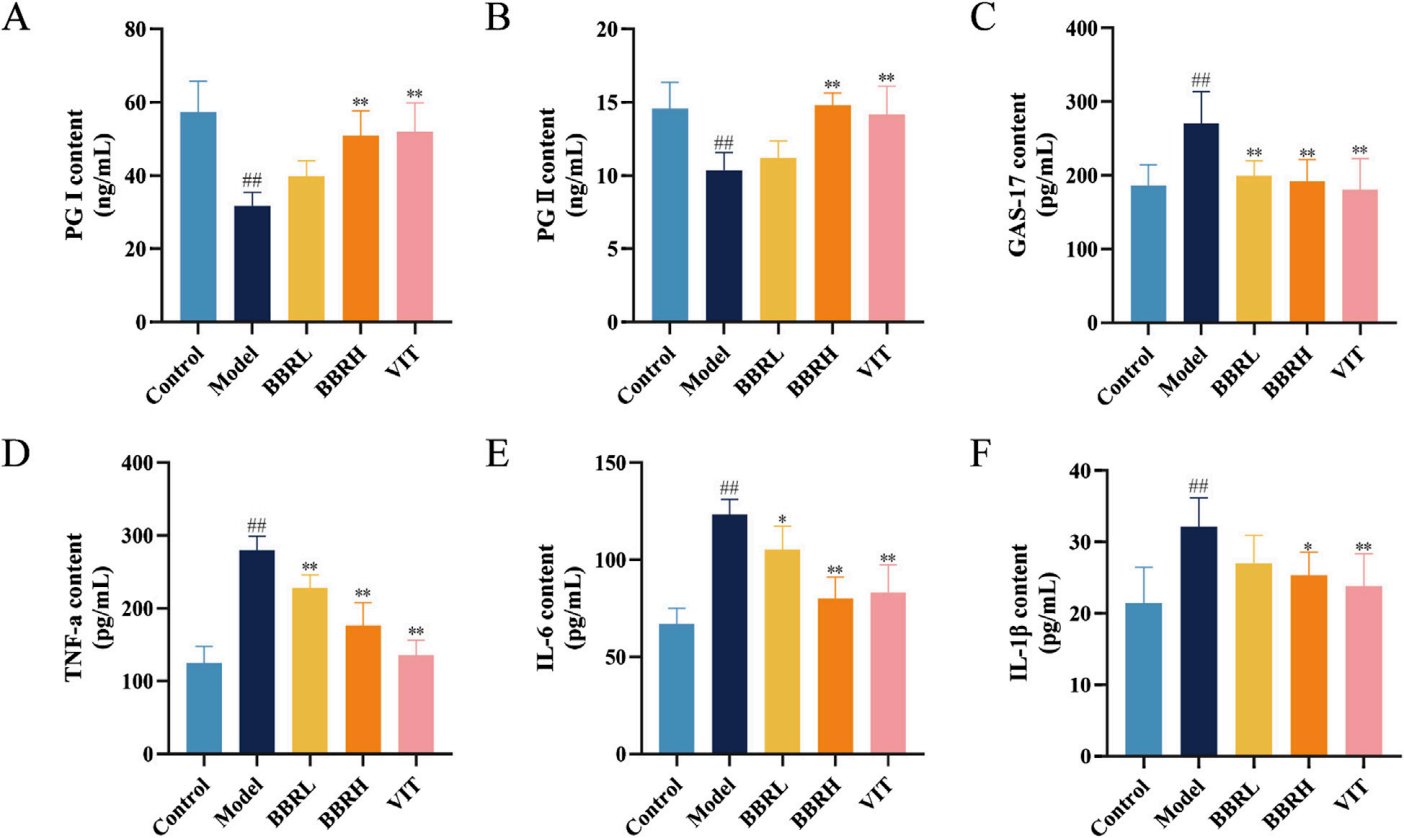
BBR improved inflammatory injury and specific biomarkers of CAG. Effect of BBR on serum levels of PG I **(A)**, PG II **(B)**, GAS-17 **(C)**, TNF-α **(D)**, IL-6 **(E)** and IL-1β **(F)** in CAG rats. All data were expressed as mean ± SD. ^#^ *P* < 0.05 and ^##^ *P* < 0.01 vs. the control group. *P < 0.05 and **P < 0.01 vs. the model group (n = 6).

To elucidate the activities of various specific markers and inflammatory cytokines associated with CAG, the serum levels of PG I, PG II, GAS-17, TNF-α, IL-6, and IL-1β were estimated. As illustrated in Figures 5A–F, the levels of PG I and PG II were significantly reduced in the model group compared with the control group. However, following BBR administration, the levels of PG I and PG II markedly increased, with the BBRH group showing higher levels than the BBRL group. Additionally, serum levels of GAS-17, TNF-α, IL-6, and IL-1β were significantly elevated in the model group compared to the control group. After treatment with BBR and vitamins, these indices decreased substantially, with the BBRH group demonstrating greater improvement than the BBRL group.

### 3.5 BBR protected the gastric mucosal barrier in CAG rats

**FIGURE 6:**
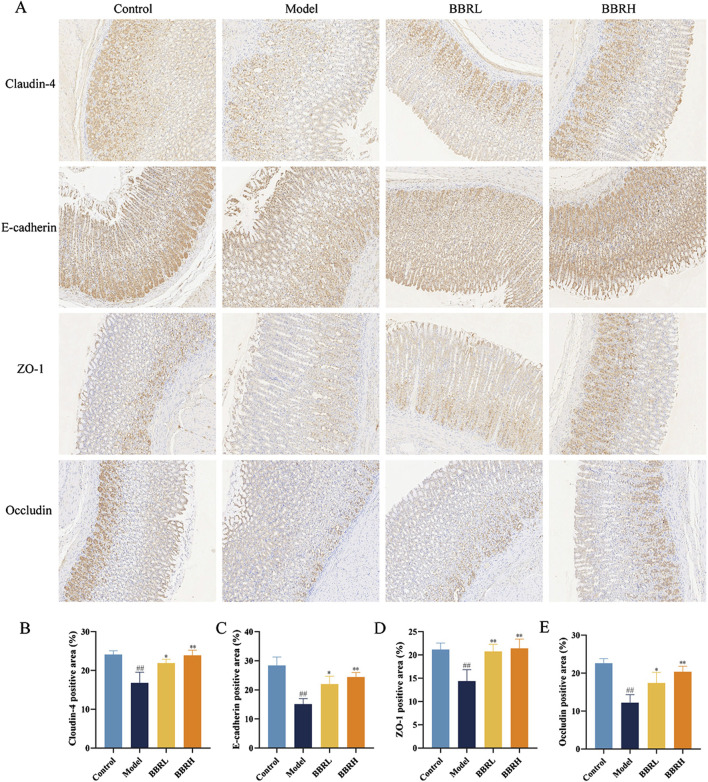
BBR protected the gastric mucosal barrier in CAG rats. **(A)** The images of Occludin, ZO-1, Claudin-4 and E-cadherin in gastric tissues of rats were measured using immunohistochemical staining (×40). **(B)** The protein expression of Claudin-4. **(C)** The protein expression of E-cadherin. **(D)** The protein expression of ZO-1. **(E)** The protein expression of Occludin. All data were expressed as mean ± SD. ^#^ *P* < 0.05 and ^##^ *P* < 0.01 vs. the control group. *P < 0.05 and **P < 0.01 vs. the model group.

To further assess BBR’s impact on the integrity of the gastric mucosal barrier, we analyzed the protein expression of Occludin, ZO-1, Claudin-4, and E-cadherin in gastric tissues. As seen in Figures 6A–E, immunohistochemical results revealed a significant reduction in the protein levels of Occludin, ZO-1, Claudin-4, and E-cadherin. However, following BBR treatment, the expression of these proteins was notably increased, suggesting that the mucosal barrier’s integrity was restored.

### 3.6 Network pharmacology analysis

**FIGURE 7:**
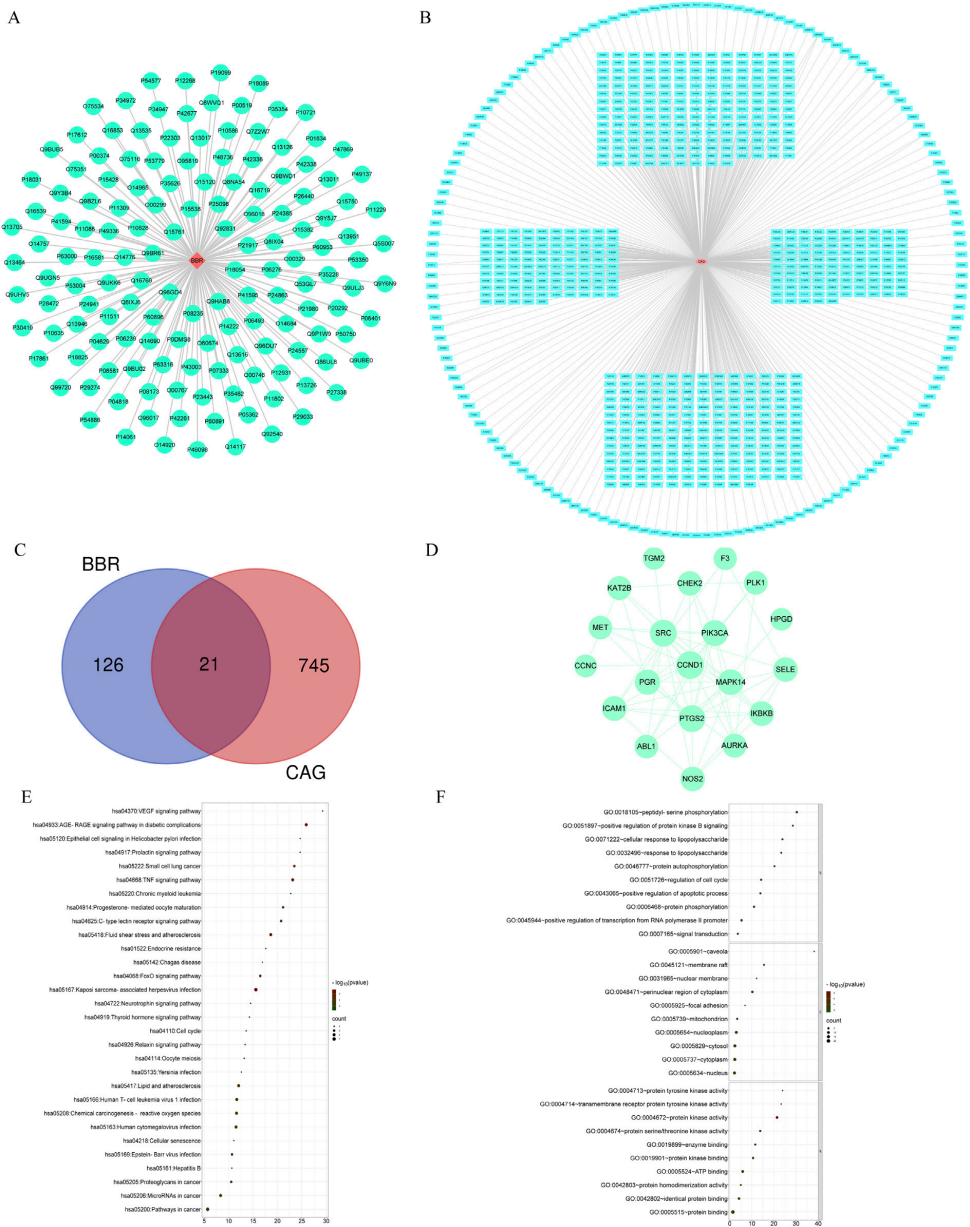
Network pharmacological analysis of BBR therapy for CAG. **(A)** “BBR-Targets” network diagram. **(B)** “CAG-Targets” network diagram. **(C)** Venn diagram. **(D)** PPI network. **(E)** Top 30 KEGG terms of ZJP in CAG. **(F)** Top 30 GO terms of ZJP in CAG.

To explore the mechanism by which BBR treats CAG, we employed network pharmacology analysis. We identified 147 targets for BBR and 766 targets associated with CAG (Figures 7A, B). Among these, 21 common targets were found between BBR and CAG, suggesting that BBR may exert its therapeutic effects on CAG through these shared targets (Figures 7C, D). Additionally, KEGG enrichment analysis revealed that the key targets were closely linked to the TNF signaling pathway, FoxO signaling pathway, VEGF signaling pathway, and others. These pathways are primarily involved in inflammatory responses, apoptosis, angiogenesis, and metabolic processes. GO enrichment analysis results indicated that BBR treatment of CAG mainly involved positive regulation of transcription from RNA polymerase II promoter and protein kinase activity, etc. The most critical 30 signaling pathways and GO terms were visualized (Figures 7E, F).

### 3.7 Transcriptomics analysis

**FIGURE 8:**
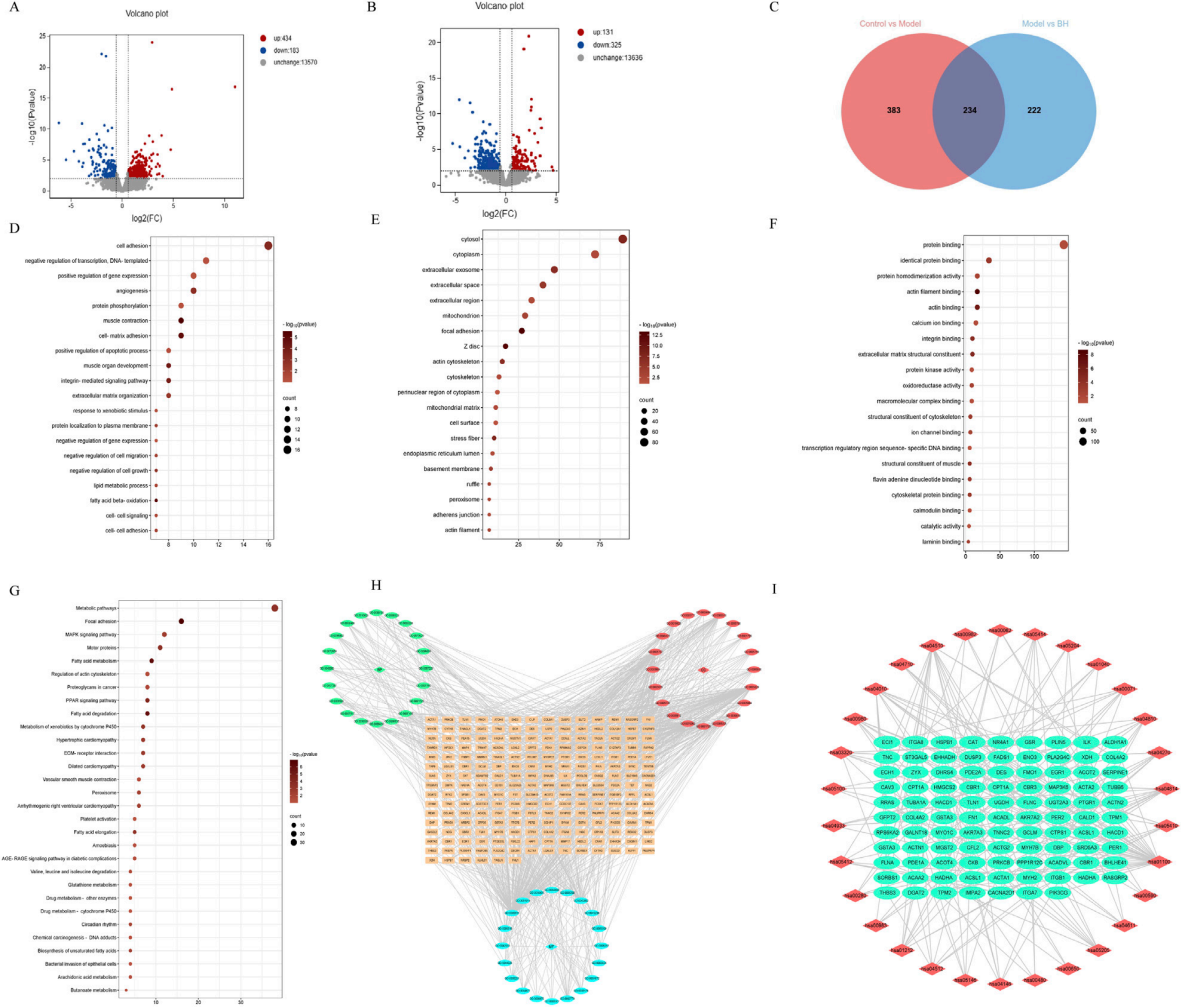
Transcriptological analysis of CAG treated with BBR. **(A)** DEGs volcano map of control vs. model. **(B)** DEGs volcano map of model vs. BBRH. **(C)** Venn diagram of differently expressed genes among control, model and BBRH groups. **(D)** The top important 20 signaling pathways of BP terms. **(E)** The top important 20 signaling pathways of CC terms. **(F)** The top important 20 signaling pathways of MF terms. **(G)** KEGG enrichment analysis of DEGs. **(H)** Gene-GO pathway network of BBR in CAG. **(I)** Gene-KEGG pathway network of BBR in CAG.

To further investigate the potential mechanisms of BBR in treating CAG, we conducted RNA sequencing on gastric tissue. We identified a total of 617 differentially expressed genes (DEGs) between the control and model groups, including 434 upregulated and 183 downregulated genes (Figure 8A). Additionally, 456 DEGs were found between the BBR treatment and model groups, comprising 131 upregulated and 325 downregulated genes (Figure 8B). Finally, 234 common DEGs (annotated genes: 216; unannotated genes: 18) were identified using Venn analysis (Figure 8C). The annotated differential genes were analyzed later. To further visualize the differences among the DEGs in the three groups, a heatmap was generated (Supplementary Figure 4). GO and KEGG pathway enrichment analyses were performed for these DEGs. As indicated by the results, the enrichment in biological functions mainly involved cell adhesion, angiogenesis and lipid metabolic process, etc. At the cell component level, the genes were involved in cytosol, cytoplasm, extracellular exosome and extracellular space, etc. At the molecular functions level, the DEGs mainly existed protein binding, identical protein binding, actin filament binding and actin binding, etc. Furthermore, KEGG enrichment analysis of DEGs identified several enriched pathways, such as metabolic pathways, MAPK signaling pathway and focal adhesion and so on. Among them, the MAPK signaling pathway is the most important pathway. The top 30 signaling pathways of KEGG and the top 20 signaling pathways of GO terms were significantly enriched, and the interaction network of corresponding targets was constructed (Figures 8D–I).

**FIGURE 9:**
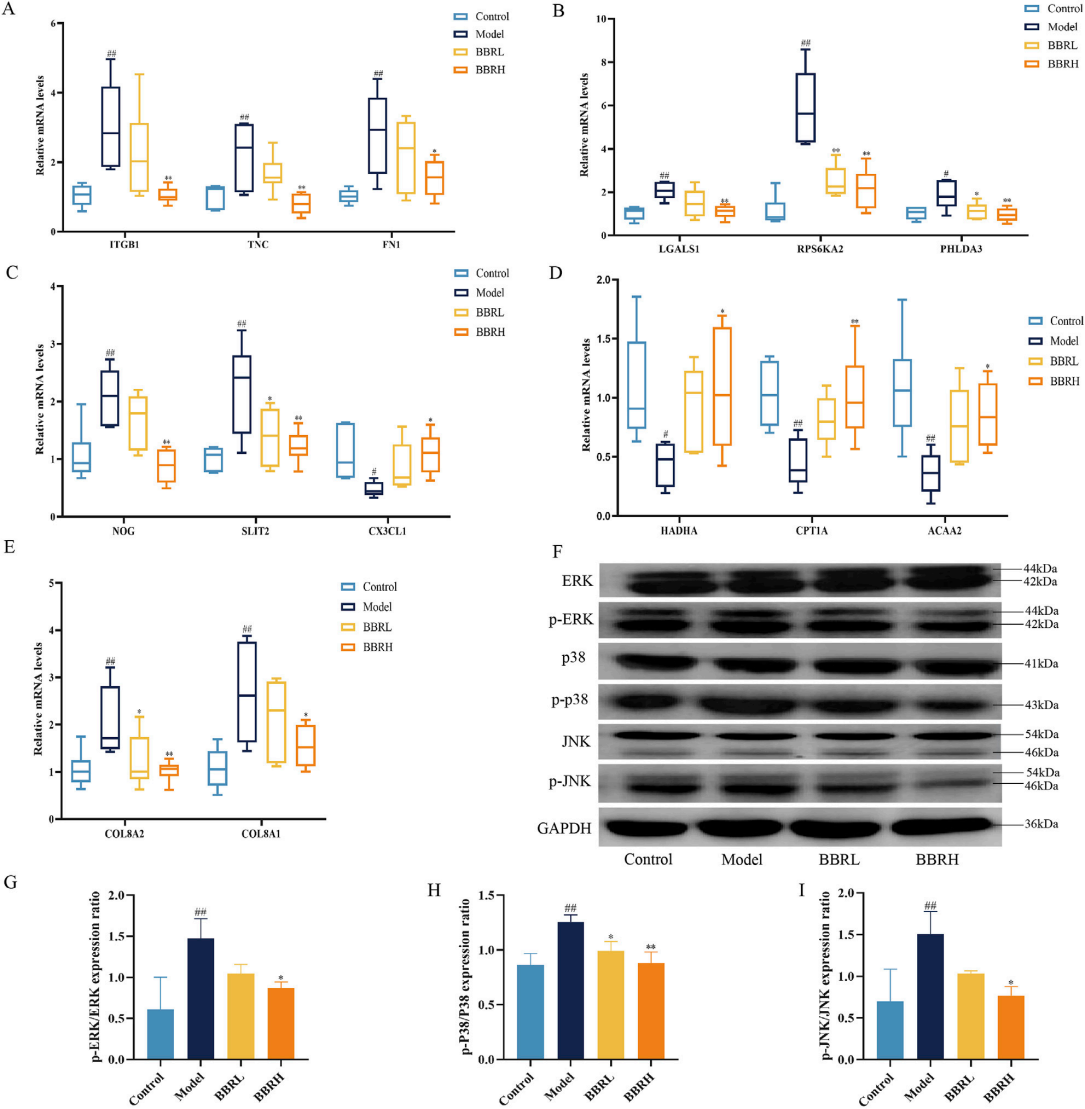
The effect of BBR on the expression of key targets and pathway. **(A)** Relative mRNA levels of cell adhesion genes. **(B)** Relative mRNA levels of apoptosis genes. **(C)** Relative mRNA levels of cell migration genes. **(D)** Relative mRNA levels of lipid metabolic genes. **(E)** Relative mRNA levels of angiogenesis genes. (n = 6). **(F)** Western blotting images of MAPK pathway. **(G)** Protein expression of p-ERK/ERK. **(H)** Protein expression of p-p38/p38. **(I)** Protein expression of p-JNK/JNK (n = 3). All data were expressed as mean ± SD. ^##^ *P* < 0.01 vs. the control group. *P < 0.05 and **P < 0.01 vs. the model group.

To further ascertain the biological function and key pathway of BBR in treating CAG, we validated the expression of several genes using RT-qPCR. These included genes related to cell adhesion (ITGB1, TNC, FN1), angiogenesis (COL8A2, COL8A1), apoptosis (LGALS1, RPS6KA2, PHLDA3), cell migration (NOG, SLIT2, CX3CL1), and lipid metabolism (HADHA, CPT1A, ACAA2). Additionally, we performed Western blotting to assess the protein levels associated with the MAPK signaling pathway. As seen in Figures 9A–E, BBR notably enhanced the expression of genes related to cell adhesion, angiogenesis, apoptosis, and cell migration, aligning with the transcriptomic data. Moreover, compared with the control group, the model group showed a significant increase in the levels of p-ERK/ERK, p-p38/p38, and p-JNK/JNK. However, these protein expressions were significantly reduced in the BBR-treated group (Figures 9F–I).

### 3.8 Widely targeted metabolomics analysis

**FIGURE 10:**
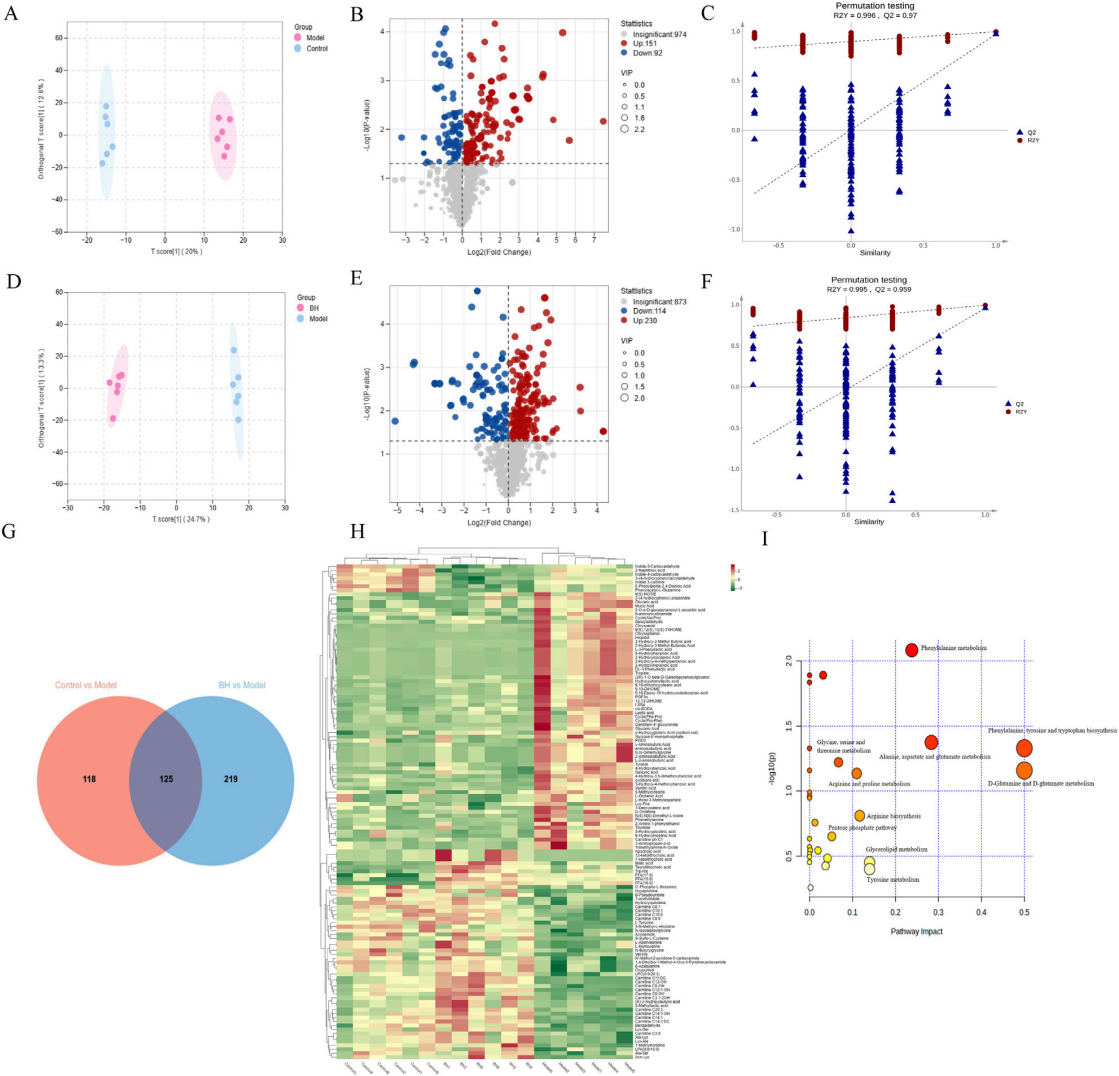
Metabolomics analysis of CAG treated with BBR. Control group vs. model group: OPLS-DA score plot **(A)**, metabolite volcano plot **(B)**, and 200-ranking test **(C)**. Model group vs. BBRH group: OPLS-DA score plot **(D)**, metabolite volcano plot **(E)**, and 200-ranking test **(F)**. **(G)** Venn diagram of every differential metabolite between control group vs. model group and model group vs. BBRH group. **(H)** Heat map of differential metabolites. **(I)** Pathway analysis was conducted by MetaboAnalyst 5.

To further explore the impact of BBR on the metabolism of CAG rats, we employed a widely targeted metabolomics approach to analyze changes in metabolites within gastric tissue. PCA was conducted on all metabolic profiles to examine global metabolic variations among rats from each group. The PCA plot revealed a clear separation among the control, model, and BBRH groups (Supplementary Figure 2). Next, the volcano plot further highlights the differential expression of metabolites, with red dots indicating those that are significantly upregulated, blue dots for those that are significantly downregulated, and gray dots for those that do not show significant changes. This visual representation underscores the distinct metabolic alterations among the groups and supports the findings from the OPLS-DA analysis, reinforcing the effectiveness of BBR treatment in modifying metabolic profiles in CAG rats (Figures 10A–F). Subsequently, 125 differential metabolites were identified among the control, model, and BBRH groups using criteria of OPLS-DA VIP >1 and *p* < 0.05. The heatmap analysis revealed that, compared to the model group, the metabolites in the BBRH group were significantly reversed. This finding indicates that BBR intervention has a regulatory effect on metabolic disorders (Supplementary Table 4). To further investigate the metabolic pathways, differential metabolites were analyzed using MetaboAnalyst 5.0. As filtered according to pathway impact size, the top 10 pathways including D-Glutamine and g-glutamate metabolism, phenylalanine, tyrosine and tryptophan biosynthesis, alanine, aspartate and glutamate metabolism, tyrosine metabolism, arginine biosynthesis, arginine and proline metabolism, glycolysis/gluconeogenesis, glycine, serine and threonine metabolism, pentose phosphate pathway and cysteine and methionine metabolism were considered to be the key signaling pathways, which are mainly involved in amino acid metabolism and lipid metabolism (Figures 10G–I; Table 1).

**TABLE 1:** Pathways enrichment analysis of differential metabolites.

Pathway Name	Match Status	p	-log(p)	Holm p	FDR	Impact
Phenylalanine metabolism	2/12	0.0082676	2.0826	0.69448	0.30687	0.2381
Nicotinate and nicotinamide metabolism	2/15	0.012877	1.8902	1.0	0.30687	0.0
Butanoate metabolism	2/15	0.012877	1.8902	1.0	0.30687	0.03175
Histidine metabolism	2/16	0.014613	1.8353	1.0	0.30687	0.0
Alanine, aspartate and glutamate metabolism	2/28	0.0423	1.3737	1.0	0.56008	0.28366
D-Arginine and D-ornithine metabolism	1/4	0.046913	1.3287	1.0	0.56008	0.0
Phenylalanine, tyrosine and tryptophan biosynthesis	1/4	0.046913	1.3287	1.0	0.56008	0.5
Glycine, serine and threonine metabolism	2/34	0.06019	1.2205	1.0	0.56008	0.06742
Nitrogen metabolism	1/6	0.069582	1.1575	1.0	0.56008	0.0
D-Glutamine and D-glutamate metabolism	1/6	0.069582	1.1575	1.0	0.56008	0.5
Arginine and proline metabolism	2/38	0.073344	1.1346	1.0	0.56008	0.10985
Ubiquinone and other terpenoid-quinone biosynthesis	1/9	0.10263	0.98871	1.0	0.68041	0.0
Aminoacyl-tRNA biosynthesis	2/48	0.10976	0.95956	1.0	0.68041	0.0
Ascorbate and aldarate metabolism	1/10	0.1134	0.94538	1.0	0.68041	0.0
Arginine biosynthesis	1/14	0.15527	0.80892	1.0	0.86949	0.11675
Glycerolipid metabolism	1/16	0.17549	0.75574	1.0	0.92134	0.01246
Pentose phosphate pathway	1/21	0.22407	0.64961	1.0	1.0	0.05183
Pyruvate metabolism	1/22	0.23346	0.63179	1.0	1.0	0.0
Glycolysis/Gluconeogenesis	1/26	0.26994	0.56873	1.0	1.0	0.0
Phosphatidylinositol signaling system	1/28	0.28756	0.54127	1.0	1.0	0.00152
Glutathione metabolism	1/28	0.28756	0.54127	1.0	1.0	0.01966
Porphyrin and chlorophyll metabolism	1/30	0.30478	0.51601	1.0	1.0	0.0
Glycerophospholipid metabolism	1/32	0.32161	0.49267	1.0	1.0	0.0
Cysteine and methionine metabolism	1/33	0.32987	0.48165	1.0	1.0	0.04179
Arachidonic acid metabolism	1/36	0.35411	0.45086	1.0	1.0	0.0
Glycerophospholipid metabolism	1/36	0.35411	0.45086	1.0	1.0	0.13987
Pyrimidine metabolism	1/39	0.37752	0.42306	1.0	1.0	0.03727
Tyrosine metabolism	1/42	0.40012	0.39781	1.0	1.0	0.13972
Purine metabolism	1/66	0.555	0.25571	1.0	1.0	0.00249

### 3.9 Comprehensive analysis of the transcriptomics and metabolomics

**FIGURE 11:**
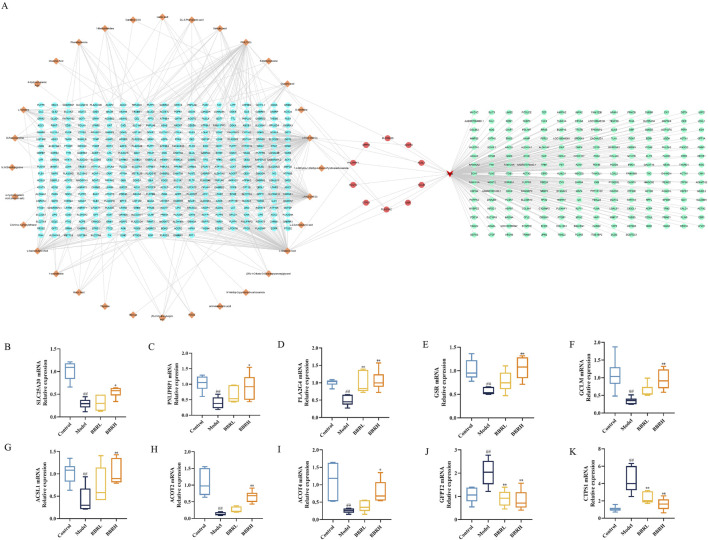
Comprehensive analysis of the transcriptomics and metabolomics. **(A)** The interaction relationship between differential metabolites and DEGs. **(B)** The relative mRNA expression of SLC25A20. **(C)** The relative mRNA expression of PNLIPRP1. **(D)** The relative mRNA expression of PLA2G4C. **(E)** The relative mRNA expression of GSR. **(F)** The relative mRNA expression of GCLM. **(G)** The relative mRNA expression of ACSL1. **(H)** The relative mRNA expression of ACOT4. **(I)** The relative mRNA expression of ACOT2. **(J)** The relative mRNA expression of GFPT2. **(K)** The relative mRNA expression of CTPS1. All data were expressed as mean ± SD. ^#^ *P* < 0.05 and ^##^ *P* < 0.01 vs. the control group. *P < 0.05 and **P < 0.01 vs. the model group.

To gain a deeper insight into the regulatory mechanism of BBR on differential metabolites in CAG, we constructed an interaction network based on key targets. The results showed that a total of 385 relevant targets for the metabolites were obtained by searching the MBrole database. Importantly, BBR directly regulated Carnitine C3:0, LPC (0:0/20:3), L-glutamic acid and FFA (15:0) by acting on solute carrier family 25 member 20 (SLC25A20), pancreatic lipase-associated protein 1 (PNLIPRP1), phospholipase A2 group 4c (PLA2G4C), glutathione reductase (GSR), glutamine-fructose-6-phosphate transaminase 2 (GFPT2), glutamate-Cysteine Ligase modifier Subunit (GCLM), CTP synthase 1 (CTPS1), long-chain acyl-CoA synthetase 1 (ACSL1), acyl-coenzyme A thioesterase 4 (ACOT4) and acyl-coenzyme A thioesterase 2 (ACOT2) (Figure 11A). Furthermore, to evaluate the effect of BBR on the key targets of the integration of metabolomics and transcriptomics, the expression levels of the key targets were determined using RT-qPCR. As illustrated in Figures 11B–K, the results showed that the expression of SLC25A20, PNLIPRP1, PLA2G4C, GSR, GCLM, ACSL1, ACOT4 and ACOT2 was dramatically reduced, and the GFPT2 and CTPS1 mRNA expression levels were significantly elevated compared to those in the control group. After the BBR intervention, the above targets were significantly reversed.

### 3.10 Intestinal flora analysis

**FIGURE 12:**
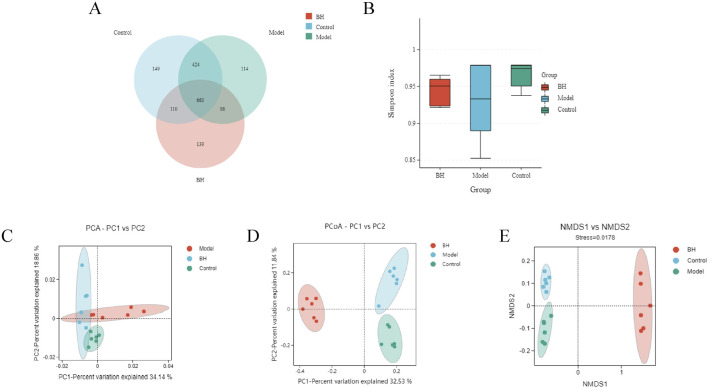
Effects of BBR on intestinal flora of α diversity and β diversity. **(A)** Gut microbiota OTU number of rats in different groups. **(B)** Simpson index. **(C)** PCA analysis. **(D)** PCoA analysis. **(E)** NMDS analysis. All data were expressed as mean ± SD. ^##^ *P* < 0.01 vs. the control group. *P < 0.05 and **P < 0.01 vs. the model group.

A total number of 1687 OTUs with 149,114,139 unique OTUs were identified in the control, model and BBRH groups, respectively (Figure 12A). The species richness and the species diversity were reflected in the analysis of the alpha diversity. The results showed that there was no significant difference in Simpson index among the control group, model group and BBR treatment group (Figure 12B). For beta diversity, the microbial community structure between the control and model groups and between the model and BBRH groups was significantly different according to the results of PCA, PCoA and NMDSD (Figures 12C–E).

**FIGURE 13:**
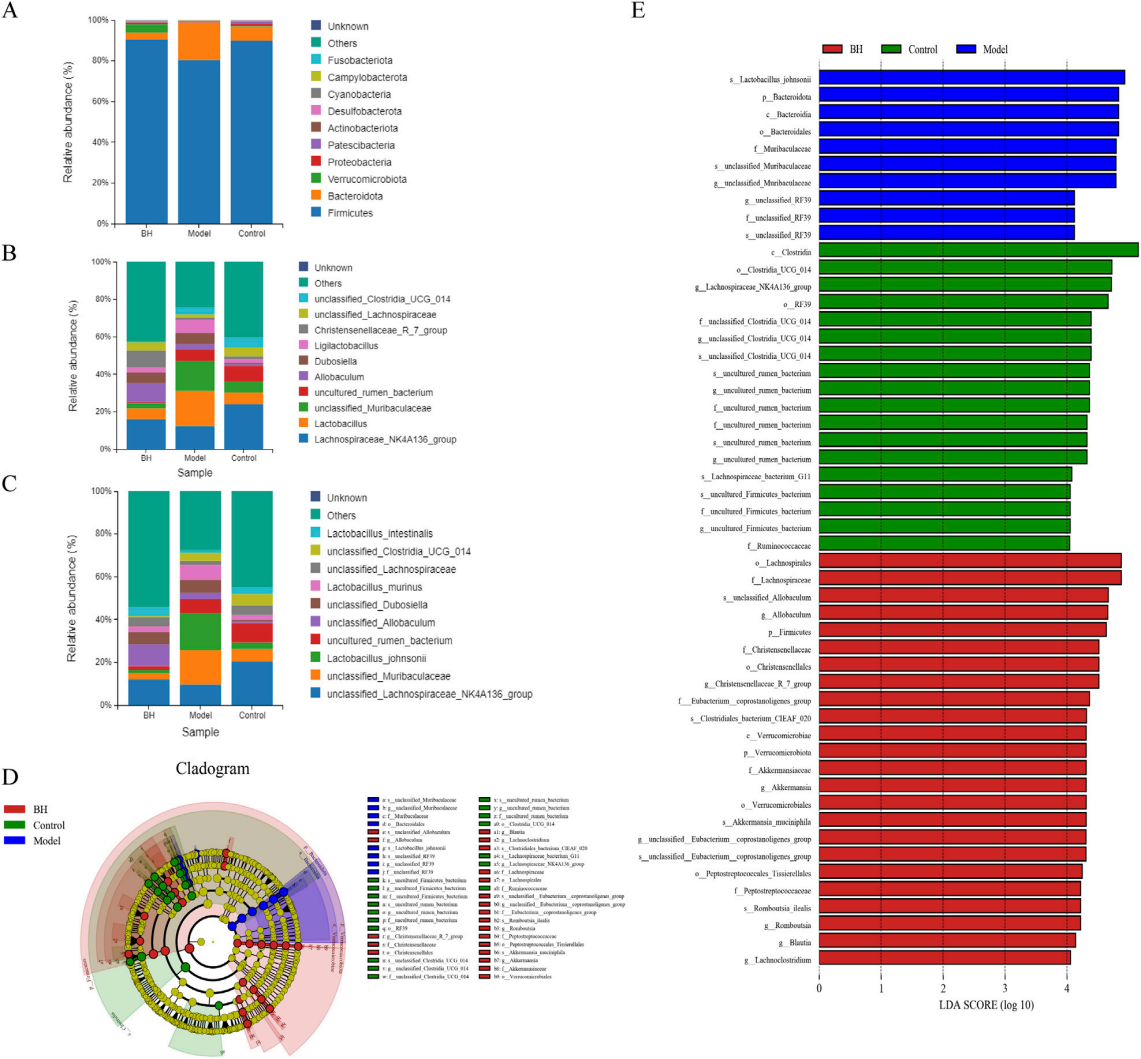
Effects of BBR intervention on the regulation of intestinal microbial structure. **(A)** Relative abundance of flora structure at the phylum level. **(B)** Relative abundance of flora structure at the genus level. **(C)** Relative abundance of flora structure at the species level. **(D)** The taxonomic cladogram obtained from LEfSe analysis. **(E)** Differential bacteria obtained by LEfSe with the threshold of LDA > 4.

At the phylum level, the predominant bacteria were *Firmicutes* and *Bacteroidota*. *Bacteroidota* was significantly higher in the model group than in the control group, while Firmicutes was significantly lower. Compared to the model group, the BBRH group showed a significant increase in *Firmicutes* but a significant decrease in *Bacteroidota* (Figure 13A). Additionally, the top three bacteria were Lachnospiraceae*_NK4A136_group*, *Lactobacillus* and *unclassified_Muribaculaceae* at the genus level. Compared with the control group, the relative abundance of Lachnospiraceae*_NK4A136_group* was remarkably decreased and the relative abundance of *Lactobacillus* and *unclassified_Muribaculaceae* was increased significantly in the model group, while after BBR intervention, the relative abundance of Lachnospiraceae*_NK4A136_group* was increased slightly and the relative abundance of *Lactobacillus* and *unclassified_Muribaculaceae* was decreased significantly compared with model group (Figure 13B). The flora structure at the level of species was further analyzed. As shown in Figure 13C, the predominant bacteria were *Lactobacillus_johnsonii, unclassified_Muribaculaceae* and *unclassified_*Lachnospiraceae*_NK4A136_group.* Compared with control group, the relative abundance of *Lactobacillus_johnsonii and unclassified_Muribaculaceae* showed an increasing trend, and the relative abundance of *unclassified_*Lachnospiraceae*_NK4A136_group* was lower in the model group. After BBR treatment, the relative abundance of these microflora was well alleviated. The LEfSe analysis is used for the identification of biomarkers with significant differences between the treatments. In this study, 52 differential bacteria were screened, including 3 phylums, 3 classes, 7 orders, 12 families, 14 genera, and 13 species. In the control group, *Clostridia*, *Clostridia_UCG_014* and Lachnospiraceae*_NK4A136_group* had the highest abundance. However, in the model group, the abundance of *Lactobacillus_johnsonii*, *Bacteroidota* and *Bacteroidia* were the highest among the three groups. In addition, the abundance of *Lachnospirales*, Lachnospiraceae and *unclassified_Allobaculum* was the highest in the BBRH group (Figures 13D, E).

### 3.11 Correlation analysis between intestinal flora and differential metabolites

**FIGURE 14:**
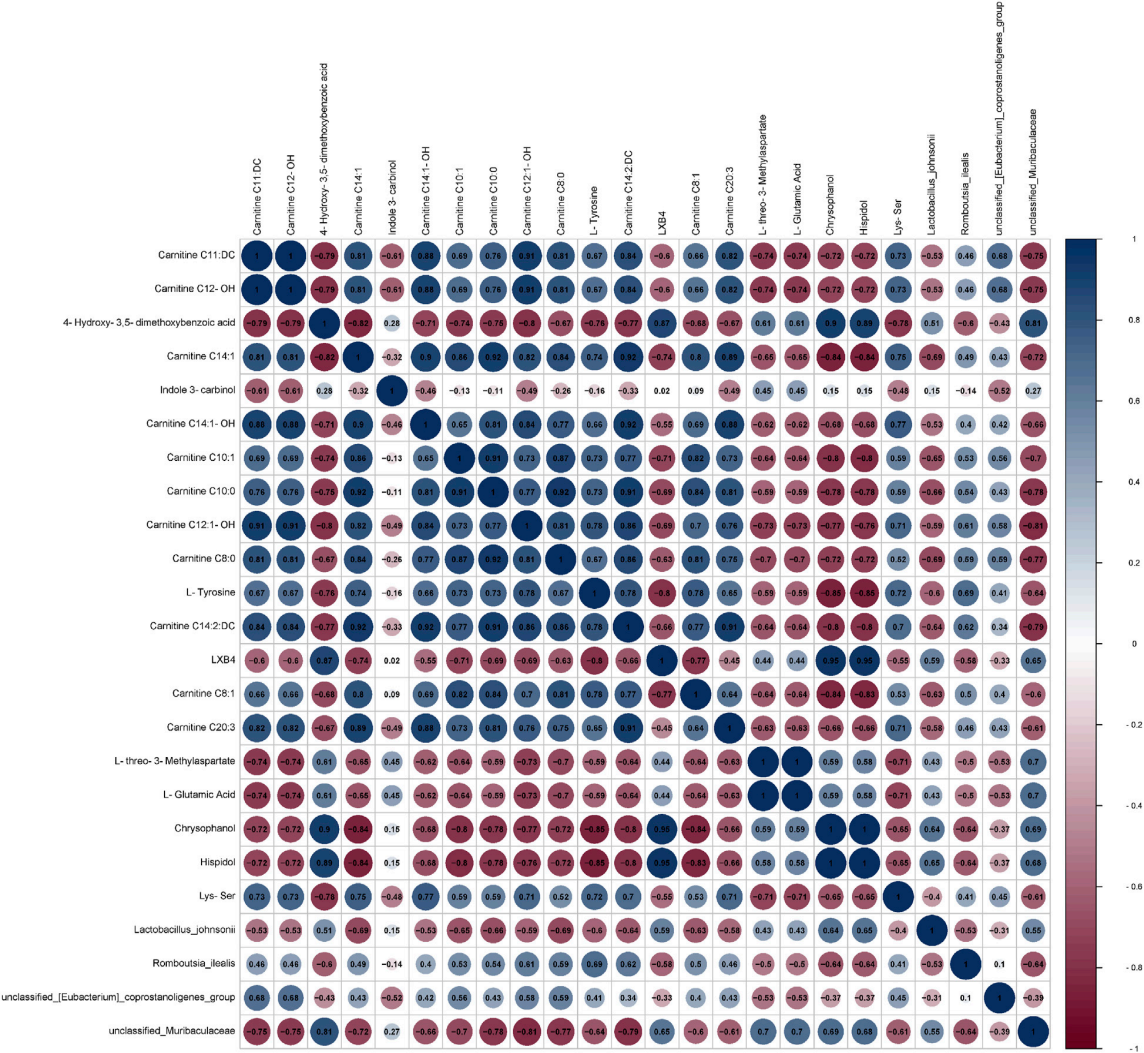
Correlation analysis between microbiota and differential metabolites.

A joint analysis of the species-level gut flora significantly regulated by BBR and the top 20 significantly regulated differential metabolites was performed to explore the relationship between differential metabolites and gut flora. As shown in Figure 14, the correlation analysis revealed that *unclassified_Muribaculaceae* had a positive correlation with 4-hydroxy-3,5-dimethoxybenzoic acid and a negative correlation with carnitine C12:1-OH(|r|>0.8).

## 4 Discussion

CAG is one of the most common gastrointestinal diseases and its annual incidence is on the rise every year. *Helicobacter pylori* infection and bile reflux are considered to be the main reasons for the formation and development of CAG (Liu et al., 2023a). The main active components of traditional Chinese medicine (TCM) have attracted much attention as important components in TCM. An increasing number of studies have shown that BBR derived from *C. chinensis* has been widely used for the treatment of CAG (Tong et al., 2021c; Yang et al., 2020). However, it is difficult to clarify its mechanism of action using traditional pharmacological methods because of its extremely complex composition and multiple targets. In the present study, we confirmed that the therapeutic effect of BBR on CAG was mainly achieved by the regulation of biological processes such as apoptosis, metabolic disorders and intestinal flora disorders via the MAPK signaling pathway through a comprehensive analysis of the gut microbiota, transcriptome, metabolomics, and molecular biology.

There is an increasing number of evidence certifying that many inflammatory cytokines have been proven to be overexpressed during the occurrence of CAG (Rudnicka et al., 2019; Tong et al., 2021b). In addition, previous studies have shown that excessive production of inflammatory cytokines helps to increase mucosal permeability (Chen et al., 2024; Ye et al., 2020). The mucosal barrier is mainly composed of tight junctions, adhesive junctions, desmosomes and mucins, which can protect the digestive tract from harmful substances and are important barriers to maintaining the stability of the digestive tract (Zhang et al., 2014). Occludin is a transmembrane protein with four transmembrane domains and 2 cell outer rings, which mainly plays a role in tight junctions. It forms tight junctions by interacting with ZO-1 (Al-Sadi et al., 2011). ZO-1 is a major tight junction spot protein, which directly binds to the cytoskeleton and occludin and is an important part of maintaining epithelial barrier function (Lagha et al., 2018). Claudin-4 is a transmembrane protein in tight junction, which is involved in regulating intercellular communication and interacting with other tight junction proteins such as occludin and ZO-1 to maintain the function of cell barrier (Fredriksson et al., 2015). E-cadherin is a kind of cadherin expressed on epithelial cell membrane, which plays a role in intercellular adhesion through its connection with actin collagen skeleton (Tunggal et al., 2005). These proteins play an important role in maintaining barrier function. Therefore, inhibition of the inflammation cascade could improve barrier function and block the progression of CAG. In the present study, we found that BBR significantly inhibited the expression of inflammatory cytokines, including TNF-α, IL-6, and IL-1β. In addition, BBR significantly increased the expression of Occludin, ZO-1, Claudin-4 and E-cadherin, thereby improving the mucosal barrier. It is well known that pepsinogen (PG) and gastrin (GAS) can be used as serological indexes in the diagnosis of chronic atrophic gastritis. GAS is mainly secreted by antral G cells. Gastric acid secretion decreased and intragastric PH increased during CAG. Then, feedback acts on the antral G cells and stimulates the release of GAS. Furthermore, some studies have found that the contents of PGI and PGII decrease when gastric mucosal injury occurs (Chen et al., 2020; Magris et al., 2020). In this study, BBR significantly increased the levels of PG I and PG II, and decreased the expression of GAS-17, revealing that BBR could effectively ameliorate CAG.

MAPK belongs to the family of serine-threonine proteases and is a basic pathway in cell biology. It is well known that the MAPK signaling pathway is mainly composed of the extracellular signal-regulated kinase (ERK), C-Jun-n-terminal kinase (JNK) and p38 (Sun et al., 2015). An increasing number of studies have shown that the MAPK signaling pathway plays an important role in regulating cell adhesion, angiogenesis, apoptosis, cell migration and lipid metabolism (Fan et al., 2024; Ferreira et al., 2023; Jiang et al., 2023; Liu et al., 2023b; Sandhu et al., 2023). CAG is a precancerous disease, and its development and evolution are affected by many factors, including aggravation of gastric mucosal injury, specific roles of angiogenic factors, effects of tumor suppressor genes and oxygen free radicals, etc. Tumor growth, invasion and metastasis are important causes of patient death, and abundant blood vessels and cell migration are important conditions for tumor growth. Therefore, inhibition of the expression of angiogenesis and migration-related genes can significantly inhibit the progression of CAG to gastric cancer (Wen et al., 2021). Cell adhesion is a dynamic process that is the most basic life activity of cells and plays a key role in cell proliferation, differentiation, migration and resistance to apoptosis (Khalili and Ahmad, 2015). Many studies have reported that E-cadherin/catenin complexes exert a role in cell adhesion (Maître and Heisenberg, 2013; Zali et al., 2009). Furthermore, intercellular adhesion molecules and vascular cell adhesion molecules are involved in the regulation of inflammation, tumor growth, migration, angiogenesis and related processes and are considered new targets for cancer treatment and clinical diagnosis of malignant tumors (Bui et al., 2020; Kong et al., 2018). It has been well-reported that apoptosis is a characteristic feature of CAG. Once the apoptosis program is initiated, a cascade of apoptotic and pathological disorders leads to tissue damage (Han et al., 2023). Therefore, suppression of gastric mucosal apoptosis can effectively block the progression of CAG. Moreover, a recent investigation has shown that lipid metabolism was obviously affected in CAG rats (Xi et al., 2021). Of note, CPT1A-mediated activation of fatty acid oxidation increases the proliferation and migration of gastric cancer cells (Wang et al., 2020). In the current study, the transcriptomic analysis indicated that BBR improved CAG by regulating cell adhesion, angiogenesis, apoptosis, cell migration and lipid metabolism via the MAPK signaling pathway.

CAG is associated with abnormal amino acid metabolism. Amino acids are the basic units of proteins and are precursors for tumor cells to synthesize purines and pyrimidines. The rapid growth and uncontrolled proliferation of tumor cells require a large number of nutrients, especially the amino acids needed to synthesize tumor cell proteins and nucleic acids, which will inevitably lead to the disorder of amino acid metabolism in patients (Yu et al., 2011). Therefore, the regulation of amino acid metabolism can block the transformation from CAG to gastric cancer. Previous studies have shown that tyrosine phosphorylation is the cause of atrophic gastritis, and the levels of tyrosine and phenylalanine in patients with early gastric cancer or atrophic gastritis are significantly higher than those in healthy people (Deng et al., 2011; Deng et al., 2012), which is consistent with our results. In addition, L-glutamic acid plays an important role in regulating the metastasis and invasion of gastric cancer cells. A recent investigation has shown that L-glutamic acid inhibits the growth of gastric cancer through the Notch signaling pathway (Sreeranganathan et al., 2017). Mounting evidence supported that glycerophospholipid metabolism was obviously affected in CAG rats (Chen et al., 2020; Xi et al., 2021). Lysophosphalipids are an important intermediate of glycerol phospholipid metabolism, which can promote the release of arachidonic acid through different signaling mechanisms (Uehara et al., 2016). Additionally, under CAG conditions, disruption of glycolysis may promote fatty acid oxidation to provide the required energy, during which the fatty acid acylcarnitine is consumed to facilitate the conversion of long-chain fatty acids into mitochondria. Fatty acid oxidation was incomplete, and lipid overload was found in the mitochondria of CAG rats (Liu et al., 2020). In the present study, BBR significantly improved amino acid metabolism and lipid metabolism by regulating differential metabolism of Carnitine C3:0, FFA (15:0), LPC (0:0/20:3), L-GlutamicAcid, etc.

By integrating transcriptome and metabolomic approaches, we identified SLC25A20, PNLIPRP1, PLA2G4C, GSR, GFPT2, GCLM, CTPS1, ACSL1, ACOT4 and ACOT2 as crucial targets involved in the regulation of differential metabolites. SLC25A20, also known as carnitine/acylcarnitine transporter (CACT), is a key molecule that transfers acyl-carnitine esters to free carnitine across the mitochondrial membrane during mitochondrial beta-oxidation (Guénard et al., 2019; Yuan et al., 2021). Mounting evidence supported that mutations in SLC25A20 are closely associated with carnitine-acylcarnitine-translocase deficiency, resulting in a variety of metabolic diseases, which further implies its critical physiological functions (Chinen et al., 2020; Vatanavicharn et al., 2015). PNLIPRP1, a member of the pancreatic triglyceride lipase family, plays a crucial role in the digestion and absorption of dietary fat (Wagner et al., 2022; Zhu et al., 2021). PLA2G4C is identified as an ortholog cPLA2a belonging to the cPAL2 family, which is a crucial gene in both the MAPK pathway and glycerophospholipid metabolism (Xia et al., 2021). Additionally, confirmatory investigations have revealed that PLA2G4C regulates the release of arachidonic acid and promotes the activation of inflammatory mediators (Chen et al., 2024). ACSL1 is a key enzyme that directs fatty acids towards β-oxidation and ceramide production. Accumulated data have further revealed that ACSL1 activates FFA β-oxidation in the liver to improve hepatic steatosis *in vitro* and *in vivo* (Zhuoyu et al., 2023). The acyl-CoA thioesterase (ACOT) family plays a vital role in lipid metabolism by maintaining an appropriate ratio of activated and free fatty acids, as well as CoA. It has been well documented that peroxisome proliferator-activated receptor alpha-mediated upregulation of ACOT4 contributes to hepatic lipid homeostasis in mice with oxidative stress induced by glutathione depletion (Konstandi et al., 2013; Yamauchi et al., 2011). In addition, upregulation of ACOT4 can promote the biosynthesis of ω-3 and ω-6 polyunsaturated fatty acids, which play an important role in regulating blood lipids and preventing the formation of atherosclerosis (Dessì et al., 2013). Furthermore, recent studies have revealed that upregulation of ACOT2 promotes adipocyte differentiation (Shen et al., 2022). Taken together, BBR significantly regulated the above genes to improve lipid metabolism during CAG. Cells have a powerful endogenous antioxidant system that plays a key role in defending against ROS and repairing oxidative damage. GSR and GCLM play important roles in oxidative stress response. Increasing data suggested that the electron pairs in NADPH are extracted by flavin-containing GSR for the reduction of glutathione disulfide (GSSG) to two molecules of the thiol-containing tripeptide glutathione (GSH) (Miller et al., 2018). GCLM also plays an important role in the regulation of GSH synthesis (Hu et al., 2023). GFPT2 is a rate-limiting enzyme in the hexosamine biosynthesis pathway (HBP) that controls the flux of glucose into HBP. As reported, high GFPT2 expression is associated with poor prognosis in various tumors, including gastric cancer, lung adenocarcinoma and colorectal cancer (Ding et al., 2022; Yang et al., 2023; Zhang et al., 2018). Additionally, increased expression of GFPT2 promotes acylation of p65 to o-glcnac, and accelerates nuclear translocation of p65, thereby enhancing NF-κB activity and promoting tumor progression (Szymura et al., 2019; Yang et al., 2023). CTPS1 consists of a CTP synthetase domain and glutamine transfer domain, whose main function is to promote UTP and glutamine to form CTP. CTPS1 plays a critical role in tumor progression. Many studies have shown that CTPS1 promotes the proliferation and migration of tumor cells, thus accelerating the occurrence of tumors (Minet et al., 2023; Wu et al., 2022). In this study, Western blotting results showed that BBR could regulate the expression of these main differential genes to approximate normal values to alleviate CAG.

Homeostasis of the intestinal flora is important for regulating gastrointestinal functions, including nutrient absorption, energy metabolism, and protecting the gastrointestinal mucosa from pathogens. The imbalance of intestinal flora may lead to the impairment of intestinal barrier function, which makes harmful substances (such as bacteria and endotoxin) easily enter the blood circulation through intestinal mucosa, thus affecting the immune state and inflammatory reaction of gastric mucosa. There has been evidence that the gut microbiota may be dysregulated in gastric diseases (Man et al., 2023). In CAG, an imbalance in the gut microbiota may exacerbate inflammation and gastric mucosal atrophy (Liu et al., 2022). *Firmicutes*, a class of Gram-positive bacteria, is pivotal in the production of short-chain fatty acids (SCFAs) including propionic acid, acetic acid, and butyric acid. These SCFAs not only furnish energy for cellular metabolism but also play a crucial role in maintaining the integrity of the mucosal barrier (Wang et al., 2024). It has been found that *Bacteroidota* can activate innate immune response, stimulate the increase of NF-κB p65 in macrophage nuclei and enhance the transcription of inflammatory cytokines (Wang X et al., 2023). *Unclassified_Muribaculaceae* and *Lactobacillus_johnsonii* were mainly associated with short-chain fatty acids, and amino acid metabolites (Blacher et al., 2017). Recently, a study also showed that the increase of tryptophan metabolites and propionate derived from microorganisms can alleviate gastrointestinal syndrome and promote inflammatory reactions (Guo et al., 2020). Additionally, it is well documented that *unclassified_Muribaculaceae* has an important role in the modulation of glucose and lipid metabolism and inflammation (Guan et al., 2024; Wang J. et al., 2023). *Lactobacillus_johnsonii* is a recognized probiotic. However, microbes in the gut can spill over into the blood and cause bacteremia when immune function is compromised. Furthermore, the overgrowth of lactic acid bacteria generates large amounts of lactic acid, causing lactic acidosis (Salminen et al., 2004; Uribarri et al., 1998; Zhang et al., 2024). In the study, BBR restored the balance of gut microbiota dysbiosis by significantly regulating the relative abundance of *Firmicutes* and *Bacteroidota* at the phylum level and the relative abundance of *unclassified_Muribaculaceae* and *Lactobacillus_johnsonii* at the species level. Therefore, BBR played a role in treating CAG by improving the balance of intestinal flora.

## 5 Conclusion

In summary, the mechanisms for BBR acting on CAG with the integration of network pharmacology, metabolomics, transcriptomics and gut microbiota were firstly explored. Our study provided compelling evidence that BBR ameliorated CAG by regulating inflammatory response, apoptosis, angiogenesis through the modulation of metabolic processes, intestinal flora and MAPK signaling pathway. Integrated analysis revealed that BBR mainly modulated differential metabolisms by regulating SLC25A20, PNLIPRP1, PLA2G4C, GSR, GFPT2, GCLM, CTPS1, ACSL1, ACOT4 and ACOT2 key targets. Our study provided theoretical and experimental support for the anti-CAG mechanism of BBR. In addition, these findings indicated that multi-omics combined network pharmacological approach is an effective strategy for evaluating the complex mechanisms of traditional Chinese medicine in various disease conditions.

## Data Availability

The raw data supporting the conclusions of this article will be made available by the authors, without undue reservation.

## Abbreviations

AB-PAS, Alcian blue-periodic acid-Schiff; ACSL1, Long-chain acyl-CoA synthetase 1; ACOT4, Acyl-coenzyme A thioesterase 4; ACOT2, Acyl-coenzyme A thioesterase 2; BBR, Berberine; BBRL, Berberine low dose; BBRH, Berberine high dose; CAG, Chronic atrophic gastritis; CTPS1, CTP synthase 1; GSR, Glutathione reductase; GFPT2, Glutamine-fructose-6-phosphate transaminase 2; GCLM, Glutamate-Cysteine Ligase Modifier Subunit; HE, Hematoxylin and eosin; IL-10, Interleukin-10; IL-6, Interleukin-6; MTL, Motilin; OPLS-DA, Orthogonal partial least squares discriminant analysis; PCA, Principal component analysis; PDE4, Phosphodiesterase 4; PVDF, Polyvinylidene difluoride; PNLIPRP1, Pancreatic lipase-associated protein 1; PLA2G4C, Phospholipase A2 group 4c; PP, Pepsin; ROS, Reactive oxygen species; SS, Somatostatin; SLC25A20, Solute carrier family 25 member 20; TNF-α, Tumor necrosis factor-alpha; TCM, Traditional Chinese medicine; VIT, Vitacoenzyme.
