# Supplementary material for: Elucidation of the mechanism of berberine against gastric mucosa injury in a rat model with chronic atrophic gastritis based on a combined strategy of multi-omics and molecular biology
Source: Front Pharmacol. 2025 Jan 6;15:1499753. doi: 10.3389/fphar.2024.1499753 (PMC11743660; doi:10.3389/fphar.2024.1499753)
Supplement: Supplementary file 1 [file Table1.docx]

**Supplementary Table 1. The specific information of the kits**

| **ELISA kit** | **Manufacturer** | **Cat. No.** |
| --- | --- | --- |
| IL-6 ELISA kit | Shanghai Enzyme-Linked Biotechnology Co., Ltd | ml102828V |
| TNF-α ELISA kit | Shanghai Enzyme-Linked Biotechnology Co., Ltd | ml002859V |
| PG I ELISA kit | Shanghai Enzyme-Linked Biotechnology Co., Ltd | ml965188V |
| PG II ELISA kit | Shanghai Enzyme-Linked Biotechnology Co., Ltd | ml365188V |
| IL-1β ELISA kit | Shanghai Enzyme-Linked Biotechnology Co., Ltd | ml037361V |
| GAS-17 ELISA kit | Shanghai Enzyme-Linked Biotechnology Co., Ltd | ml920633V |
