# Supplementary figures and images for: Elucidation of the mechanism of berberine against gastric mucosa injury in a rat model with chronic atrophic gastritis based on a combined strategy of multi-omics and molecular biology
Source: Front Pharmacol. 2025 Jan 6;15:1499753. doi: 10.3389/fphar.2024.1499753 (PMC11743660; doi:10.3389/fphar.2024.1499753)

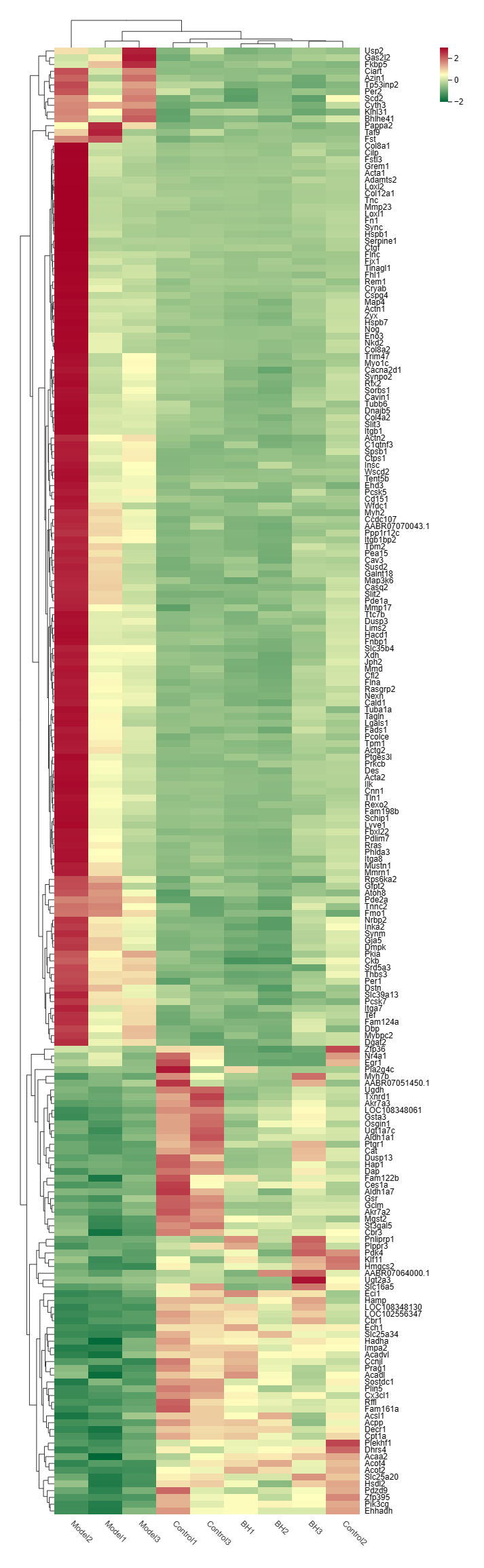

Supplement: Supplementary file 2 [file Image1.jpeg]

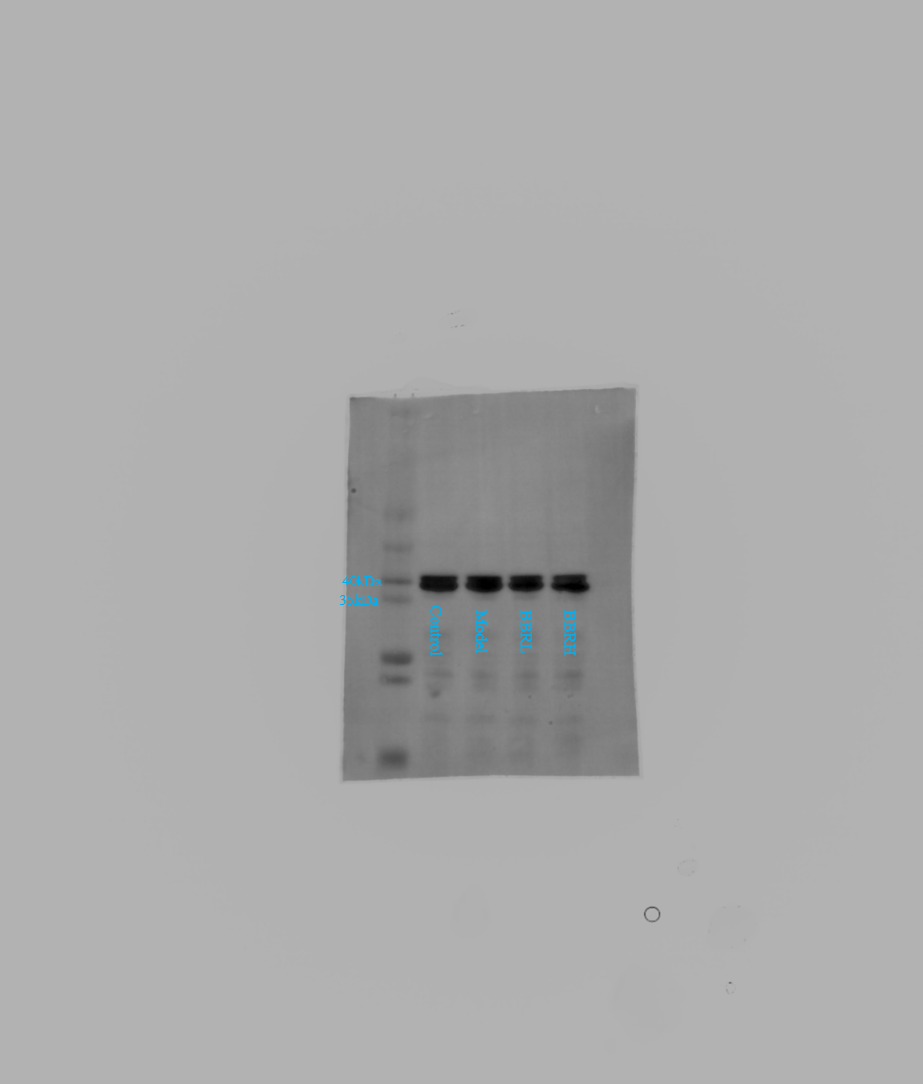

Supplement: Supplementary file 3 [file DataSheet1.zip › raw data/Original Images for Blots/ERK/1.tif]

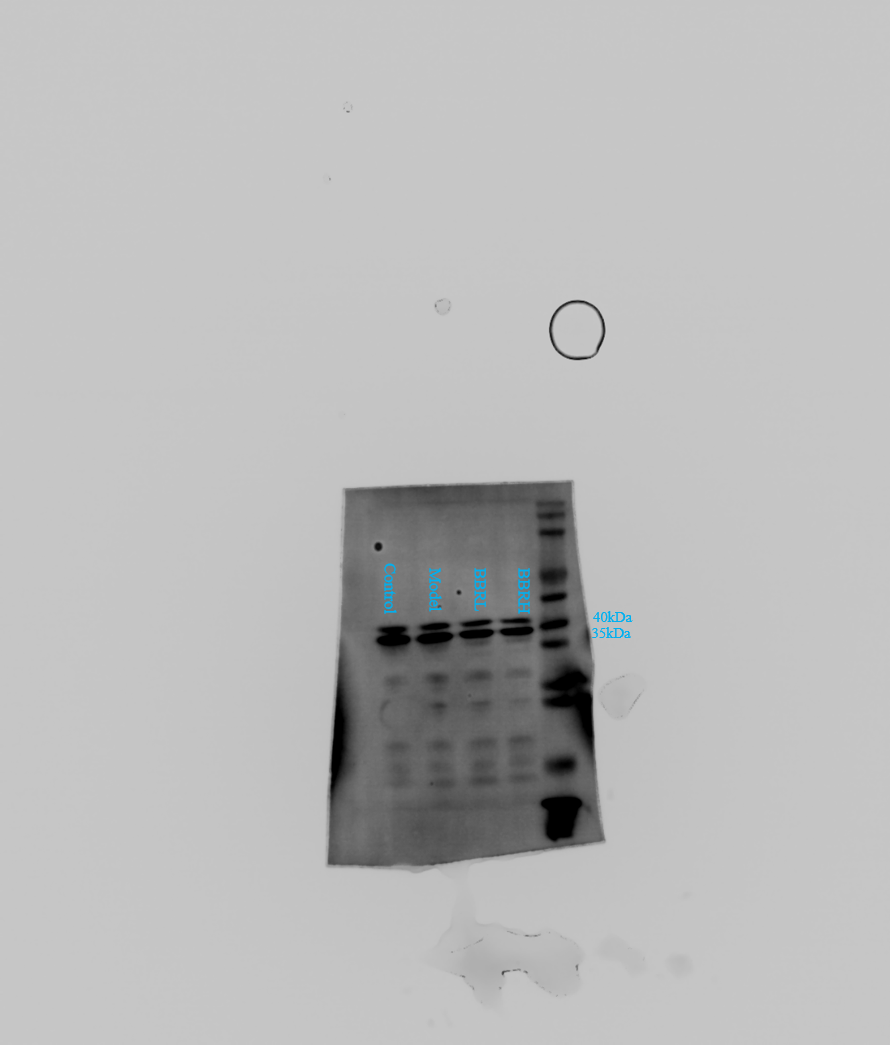

Supplement: Supplementary file 3 [file DataSheet1.zip › raw data/Original Images for Blots/ERK/2.tif]

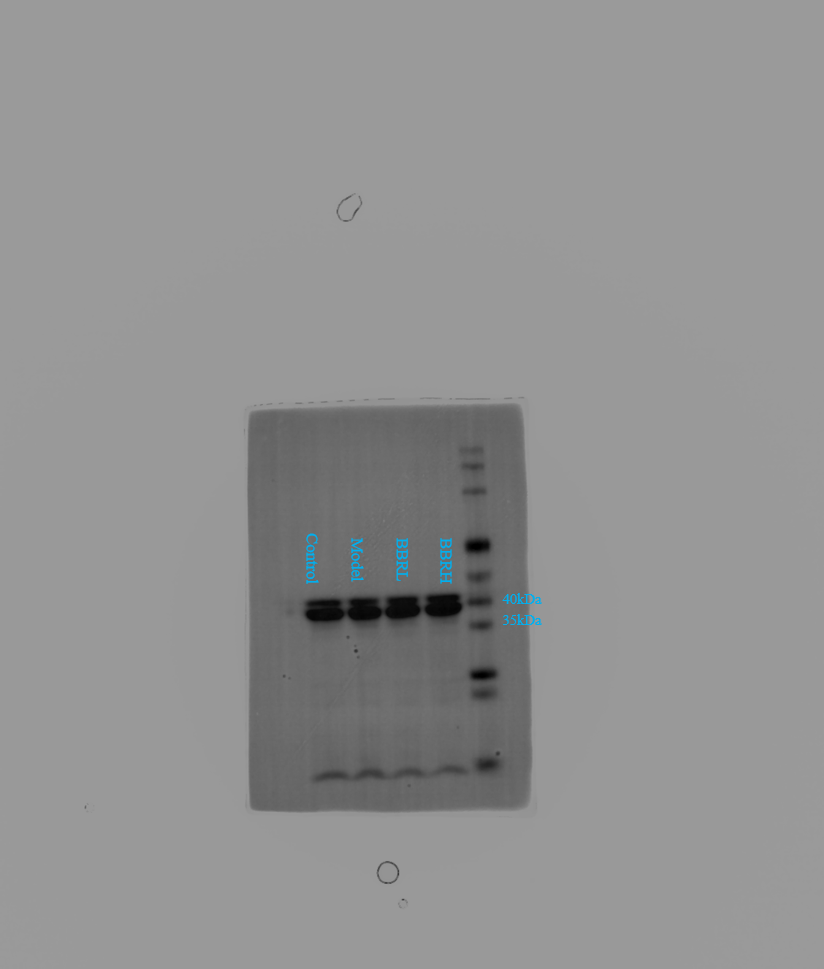

Supplement: Supplementary file 3 [file DataSheet1.zip › raw data/Original Images for Blots/ERK/3.tif]

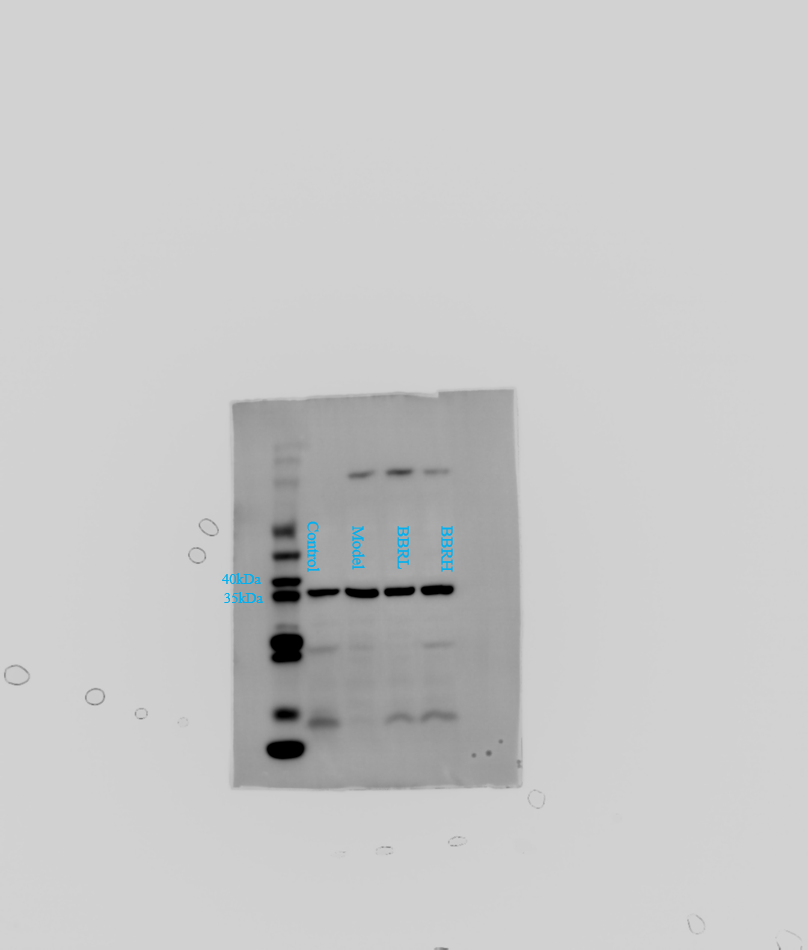

Supplement: Supplementary file 3 [file DataSheet1.zip › raw data/Original Images for Blots/GAPDH/1.tif]

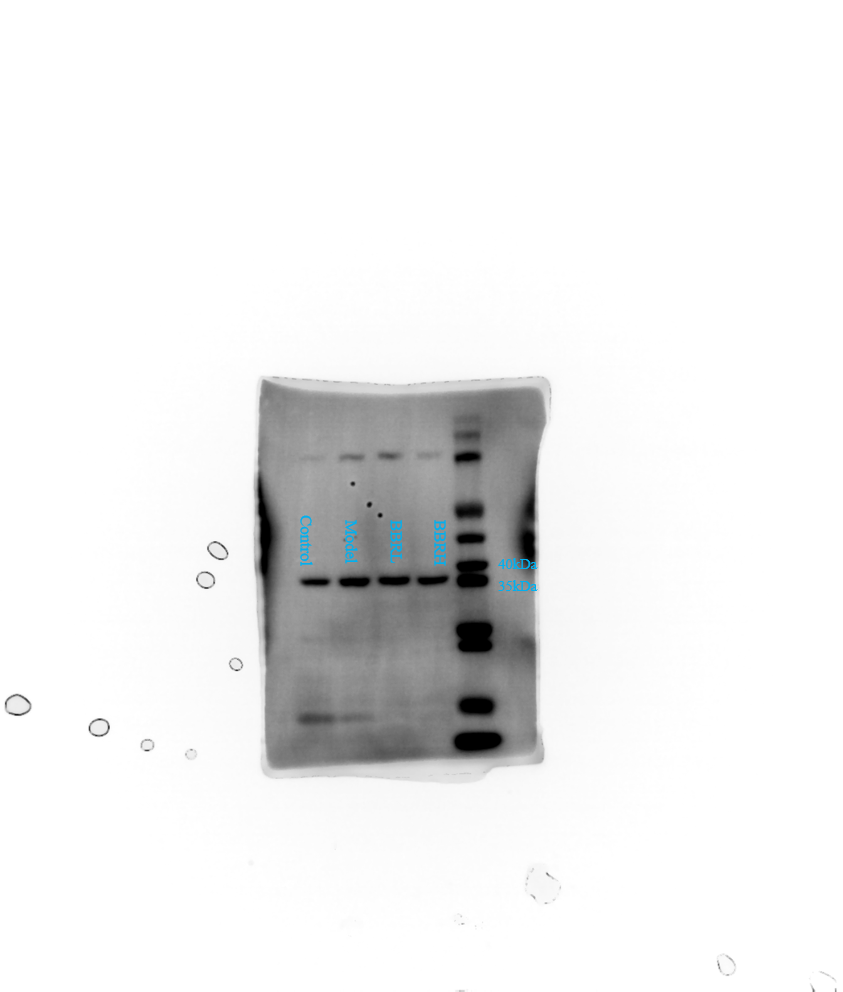

Supplement: Supplementary file 3 [file DataSheet1.zip › raw data/Original Images for Blots/GAPDH/2.tif]

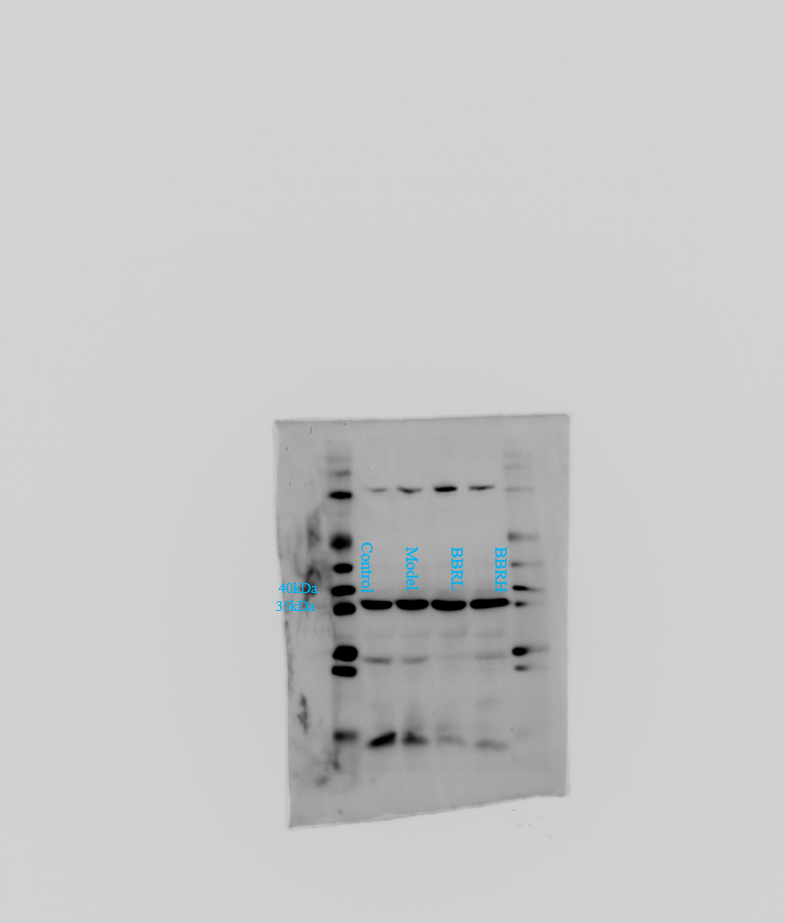

Supplement: Supplementary file 3 [file DataSheet1.zip › raw data/Original Images for Blots/GAPDH/3.tif]

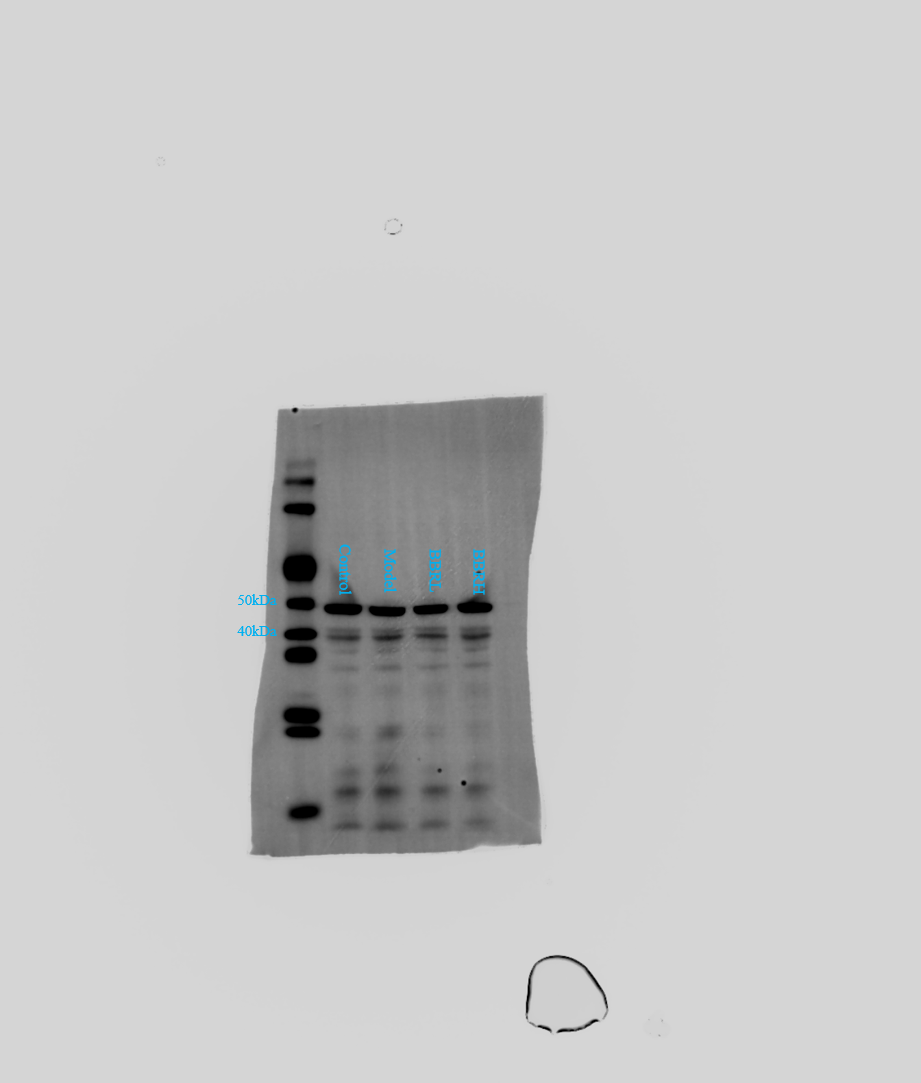

Supplement: Supplementary file 3 [file DataSheet1.zip › raw data/Original Images for Blots/JNK/1.tif]

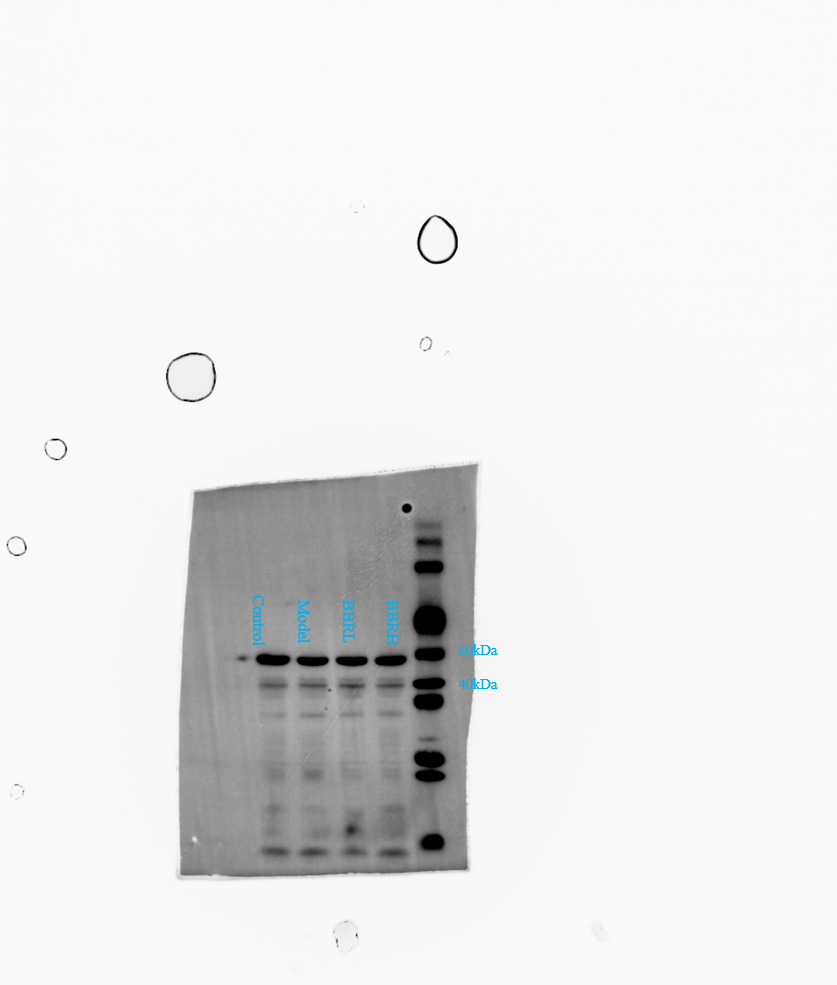

Supplement: Supplementary file 3 [file DataSheet1.zip › raw data/Original Images for Blots/JNK/2.tif]

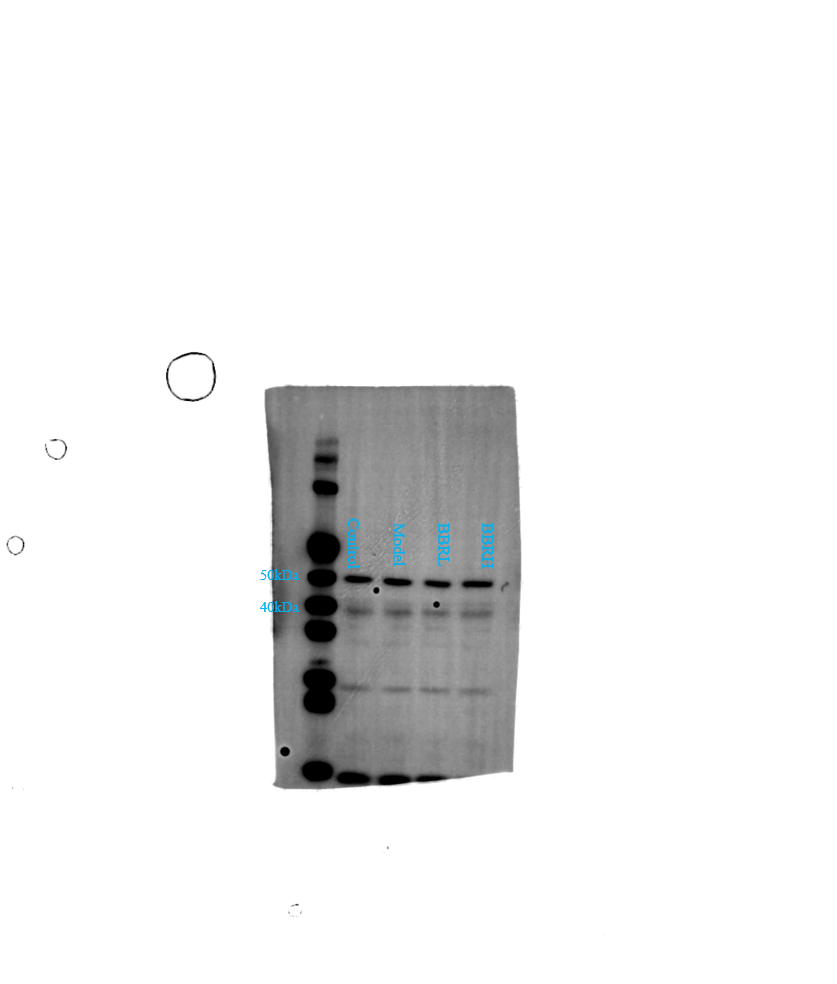

Supplement: Supplementary file 3 [file DataSheet1.zip › raw data/Original Images for Blots/JNK/3.tif]

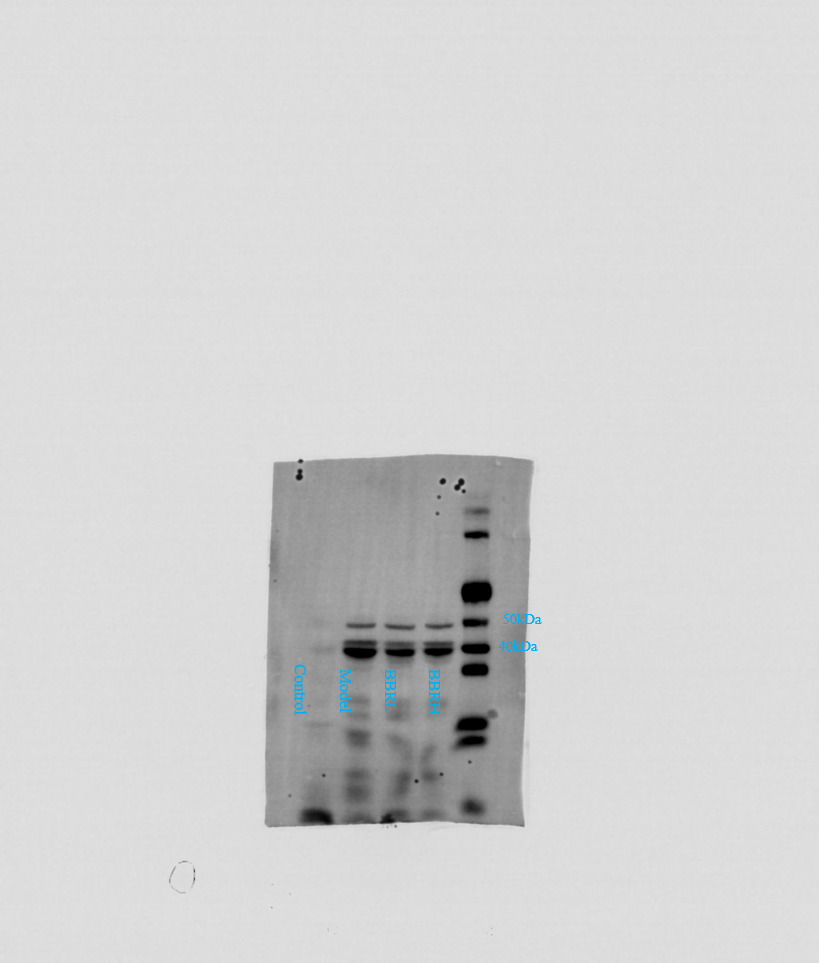

Supplement: Supplementary file 3 [file DataSheet1.zip › raw data/Original Images for Blots/P-ERK/1.tif]

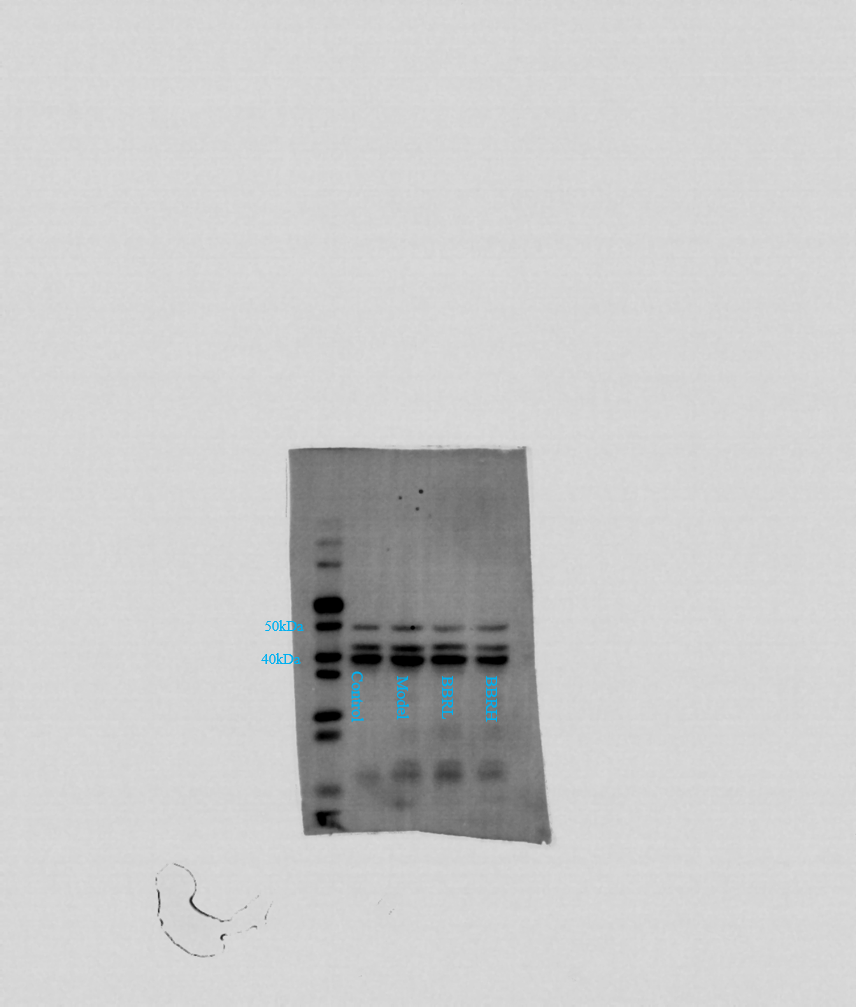

Supplement: Supplementary file 3 [file DataSheet1.zip › raw data/Original Images for Blots/P-ERK/2.tif]

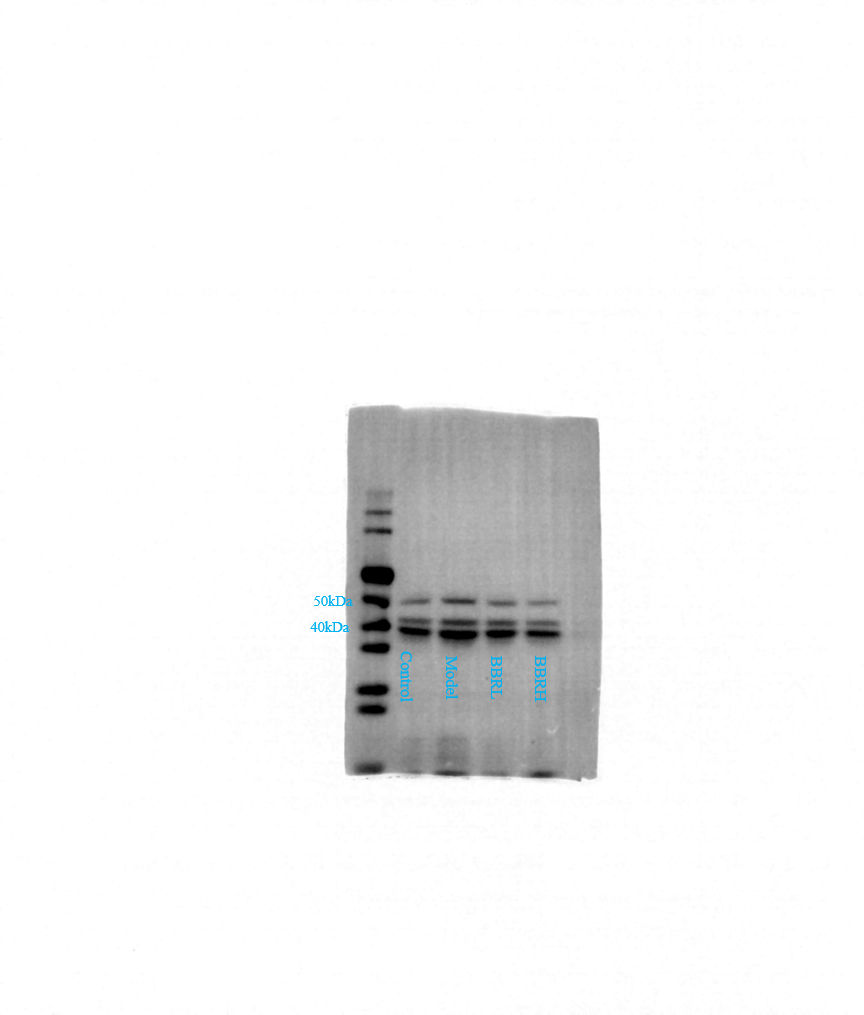

Supplement: Supplementary file 3 [file DataSheet1.zip › raw data/Original Images for Blots/P-ERK/3.tif]

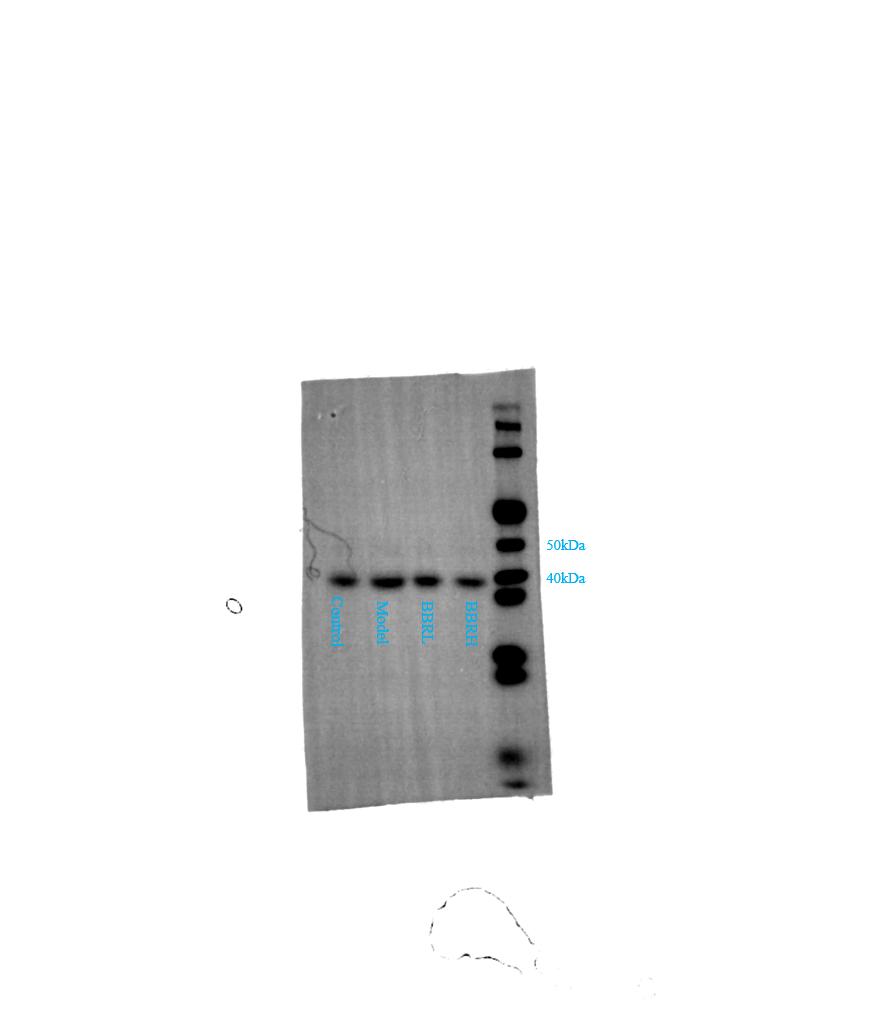

Supplement: Supplementary file 3 [file DataSheet1.zip › raw data/Original Images for Blots/p-JNK/1.tif]

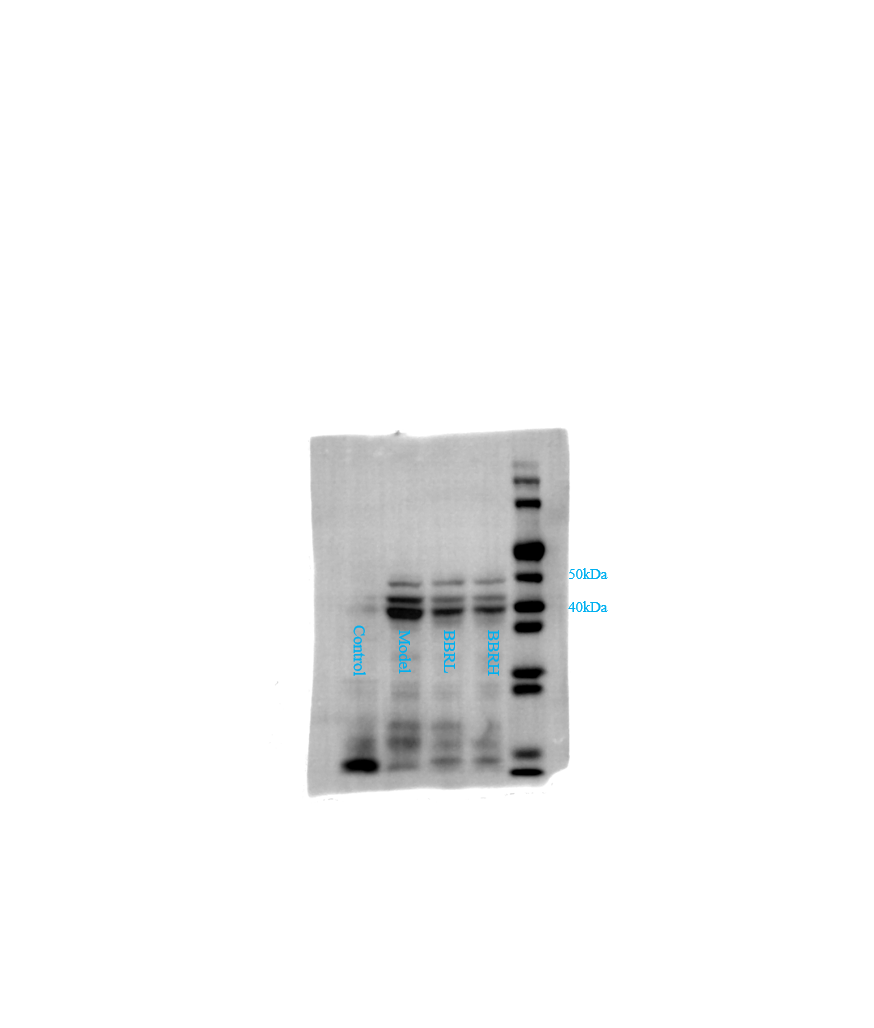

Supplement: Supplementary file 3 [file DataSheet1.zip › raw data/Original Images for Blots/p-JNK/2.tif]

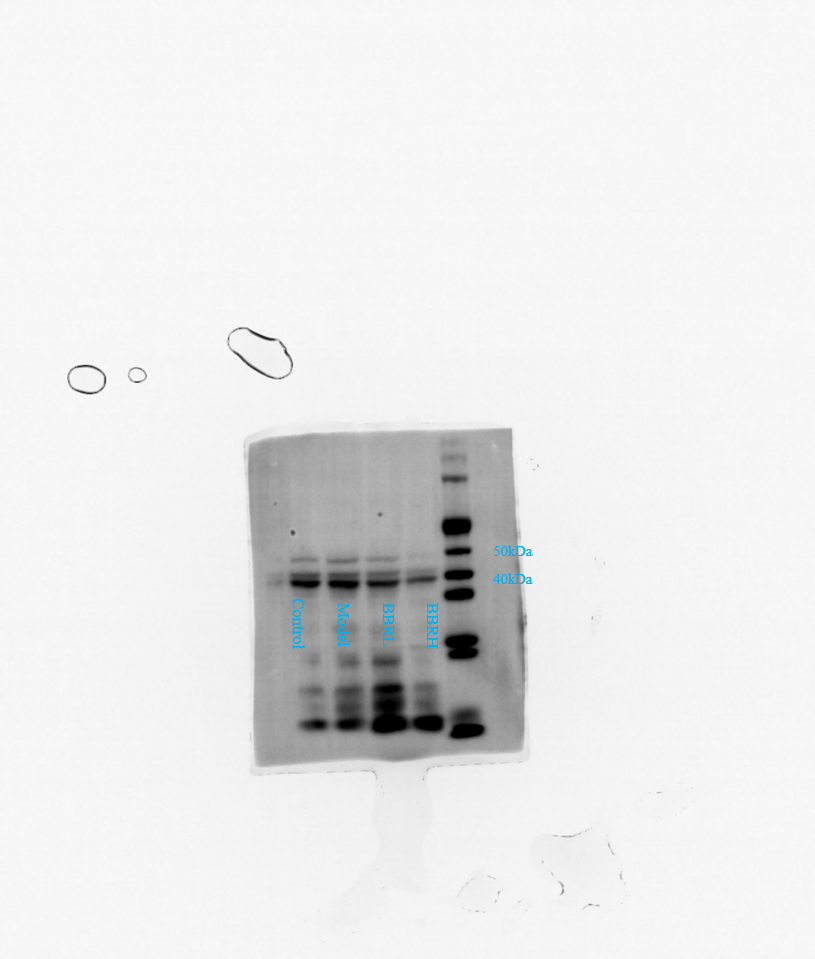

Supplement: Supplementary file 3 [file DataSheet1.zip › raw data/Original Images for Blots/p-JNK/3.tif]

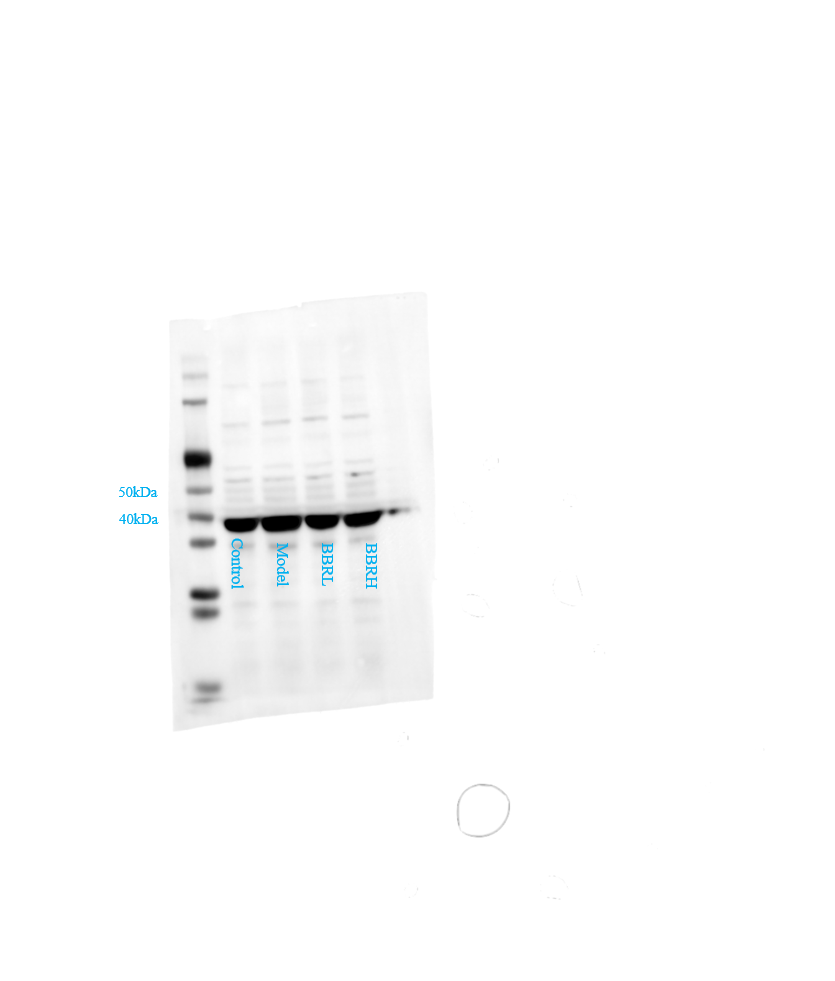

Supplement: Supplementary file 3 [file DataSheet1.zip › raw data/Original Images for Blots/p-p38/1.tif]

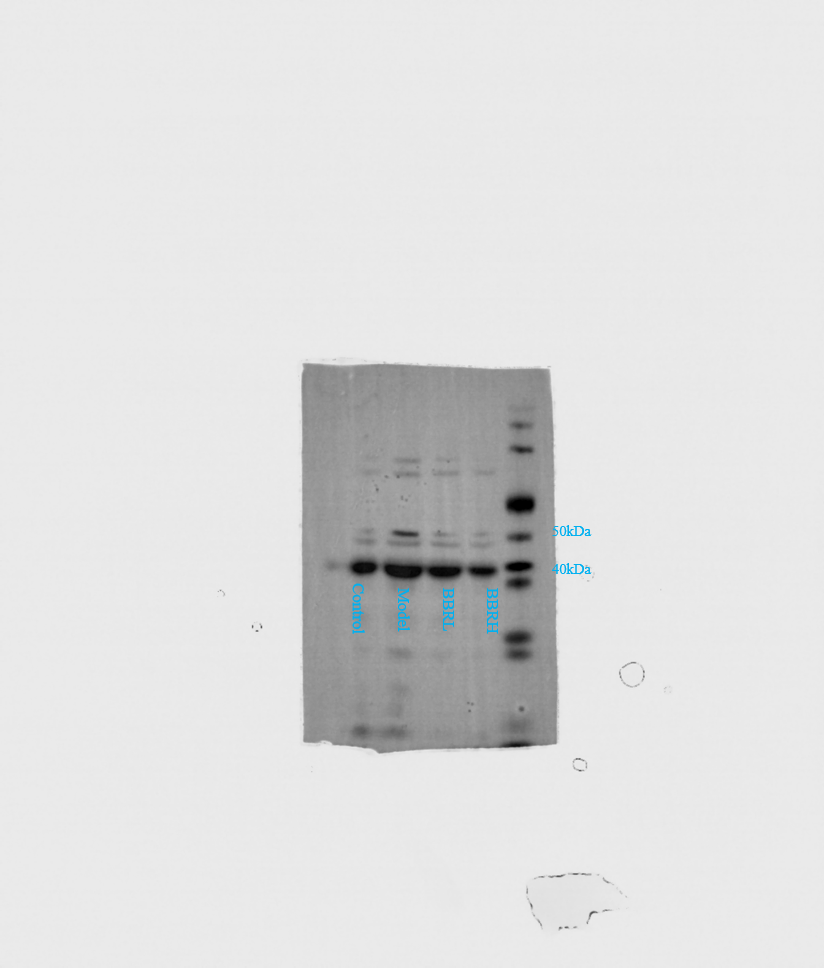

Supplement: Supplementary file 3 [file DataSheet1.zip › raw data/Original Images for Blots/p-p38/2.tif]

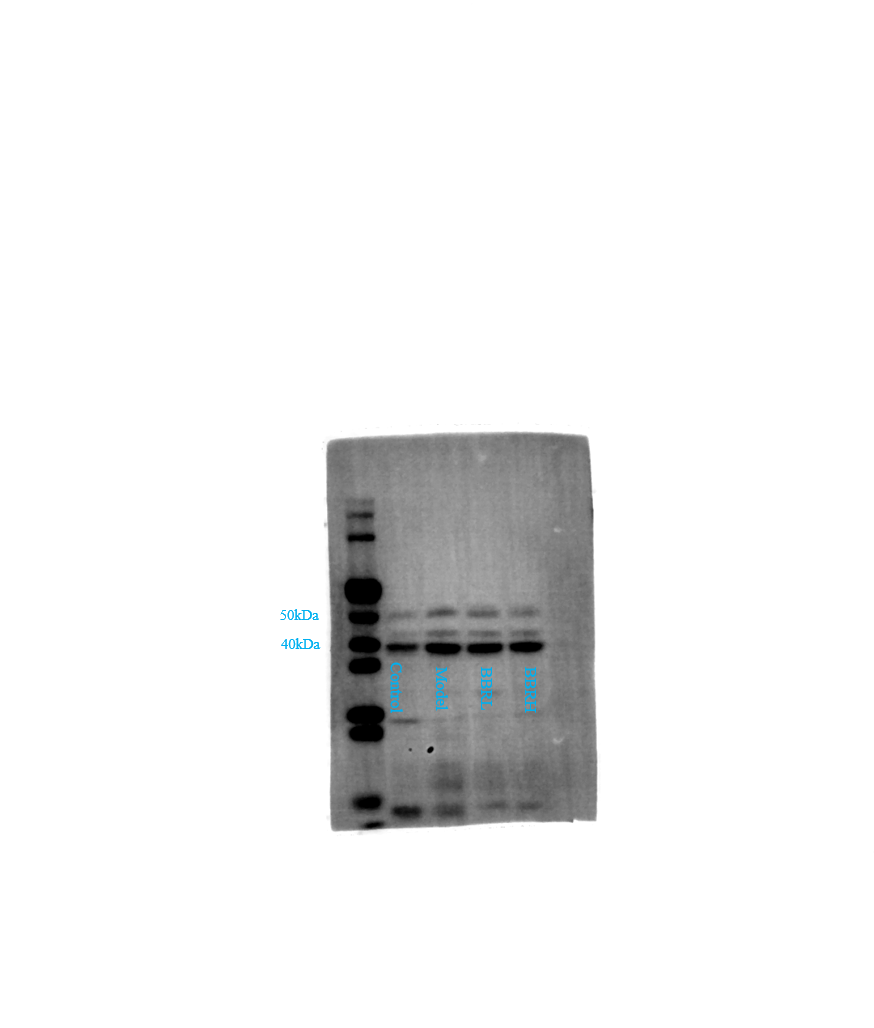

Supplement: Supplementary file 3 [file DataSheet1.zip › raw data/Original Images for Blots/p-p38/3.tif]

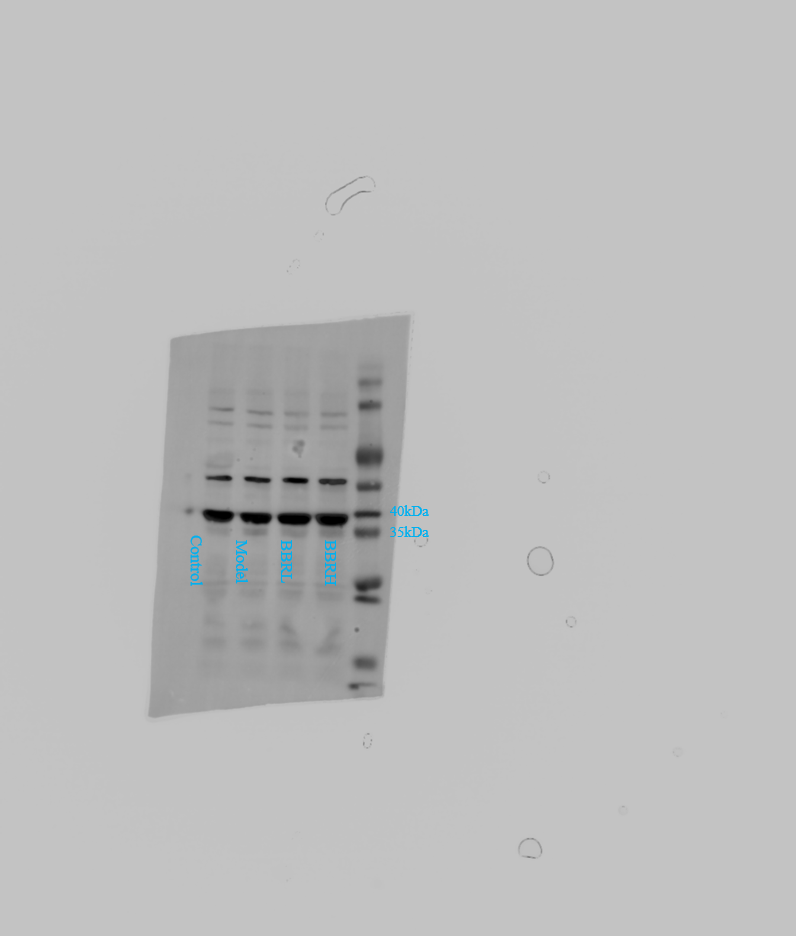

Supplement: Supplementary file 3 [file DataSheet1.zip › raw data/Original Images for Blots/p38/1.tif]

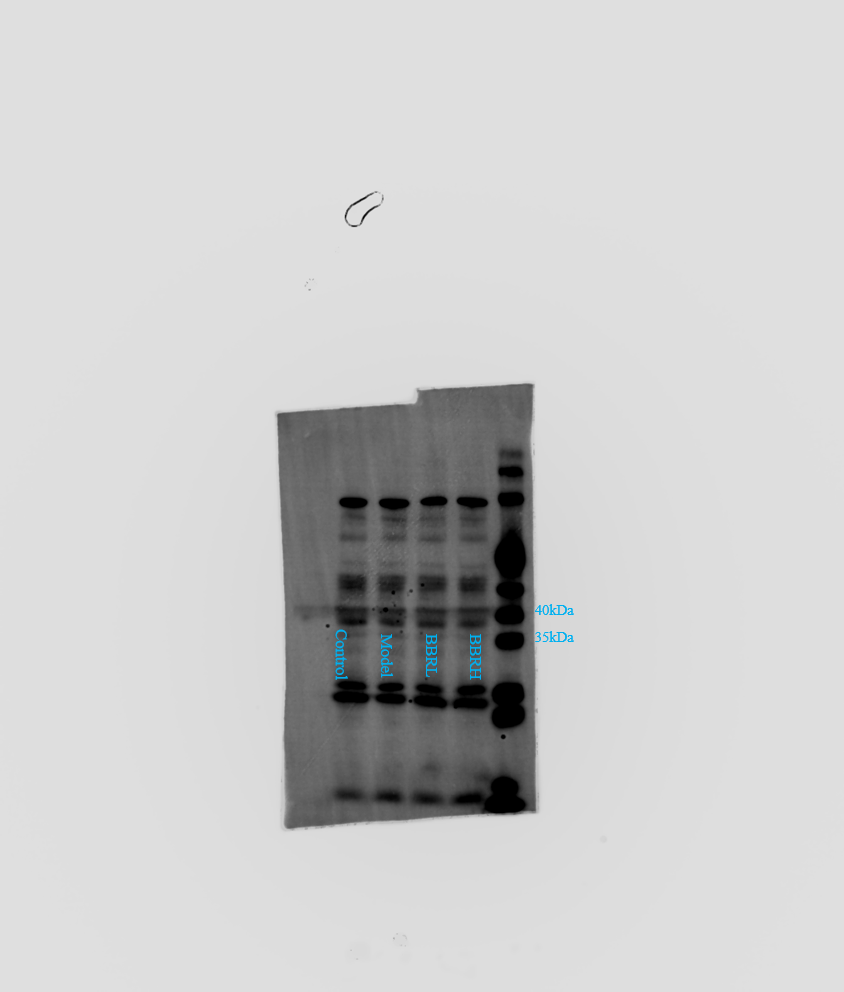

Supplement: Supplementary file 3 [file DataSheet1.zip › raw data/Original Images for Blots/p38/2.tif]

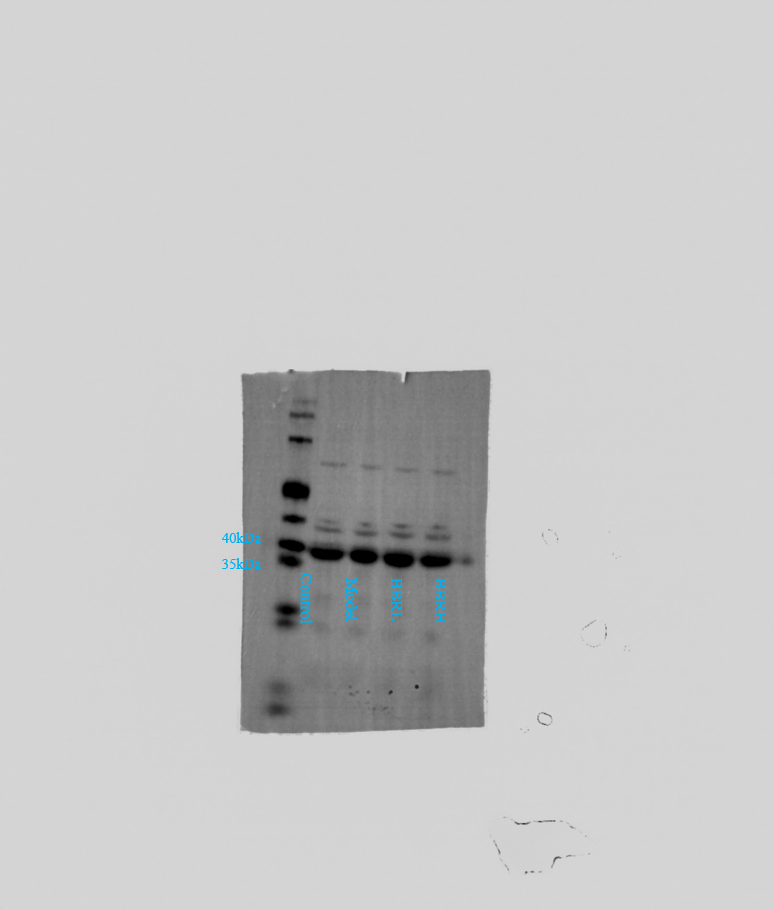

Supplement: Supplementary file 3 [file DataSheet1.zip › raw data/Original Images for Blots/p38/3.tif]

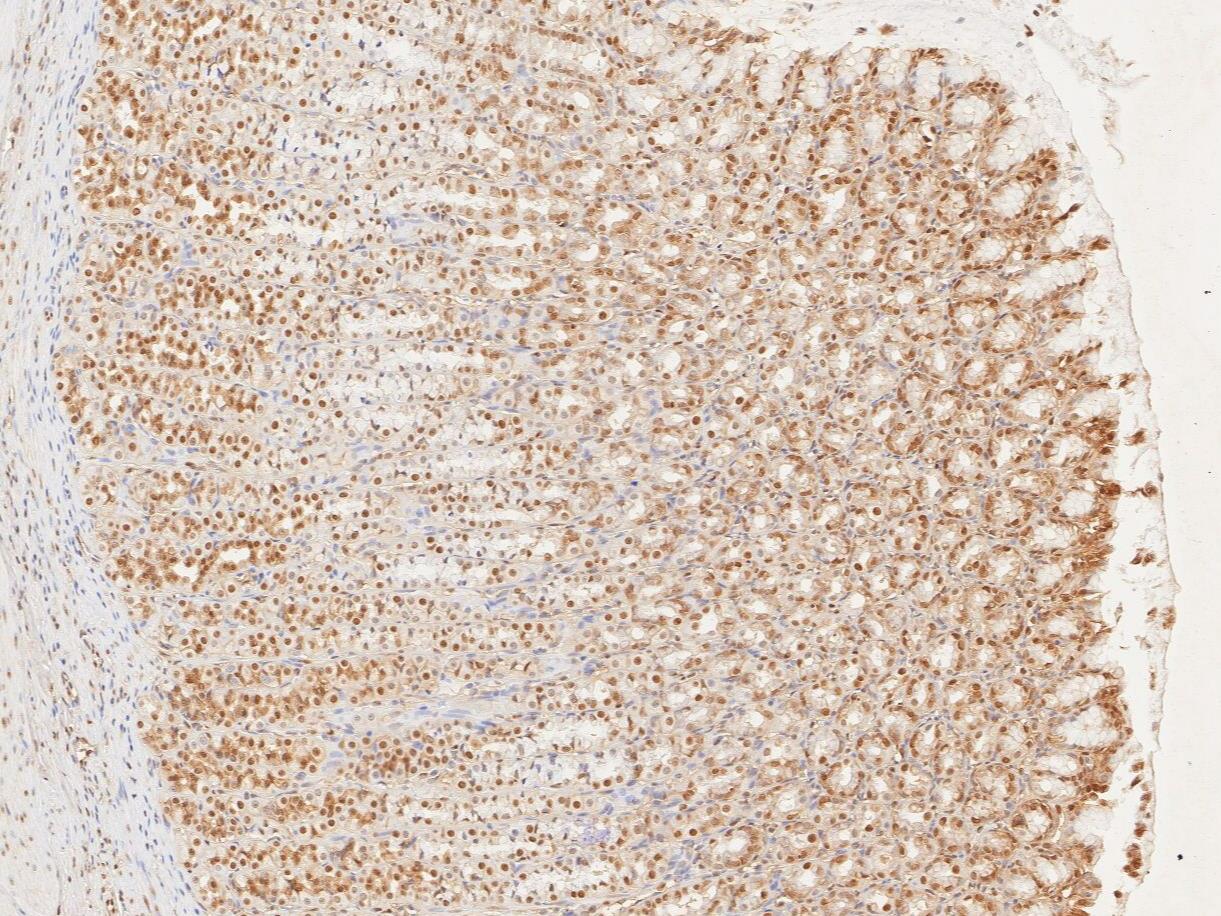

Supplement: Supplementary file 3 [file DataSheet1.zip › raw data/The images of Occludin, ZO-1, Claudin-4 and E-cadherin/E-cadherin_BH1.jpg]

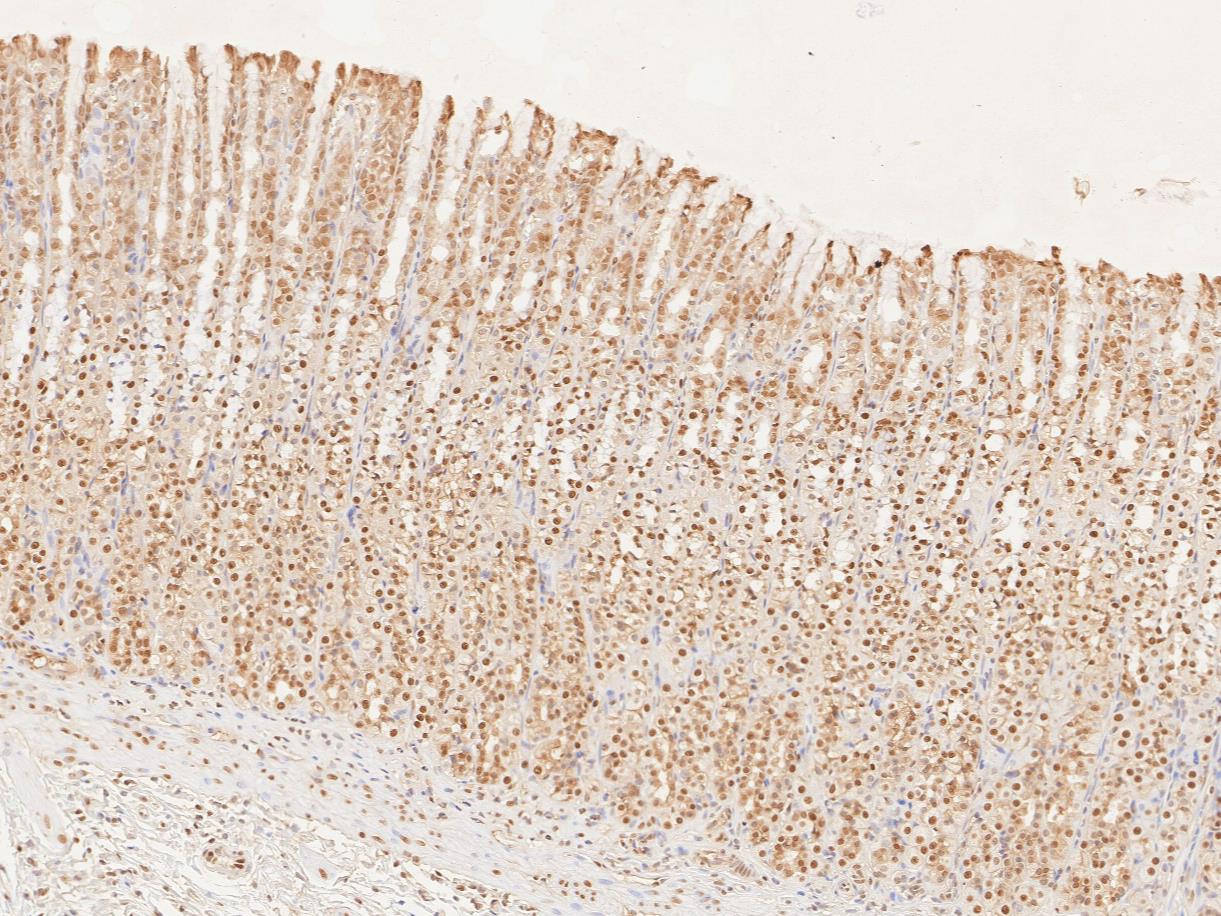

Supplement: Supplementary file 3 [file DataSheet1.zip › raw data/The images of Occludin, ZO-1, Claudin-4 and E-cadherin/E-cadherin_BH2.jpg]

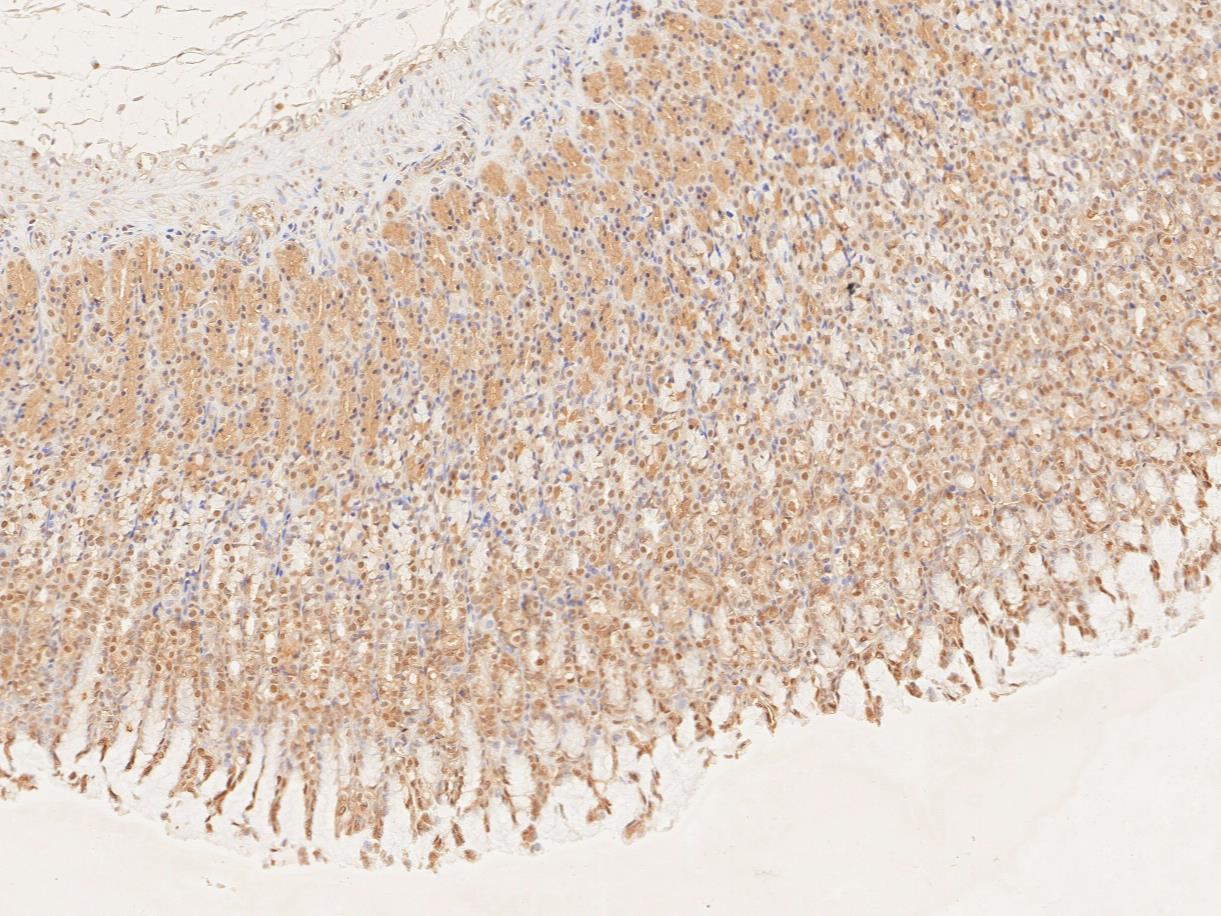

Supplement: Supplementary file 3 [file DataSheet1.zip › raw data/The images of Occludin, ZO-1, Claudin-4 and E-cadherin/E-cadherin_BH3.jpg]

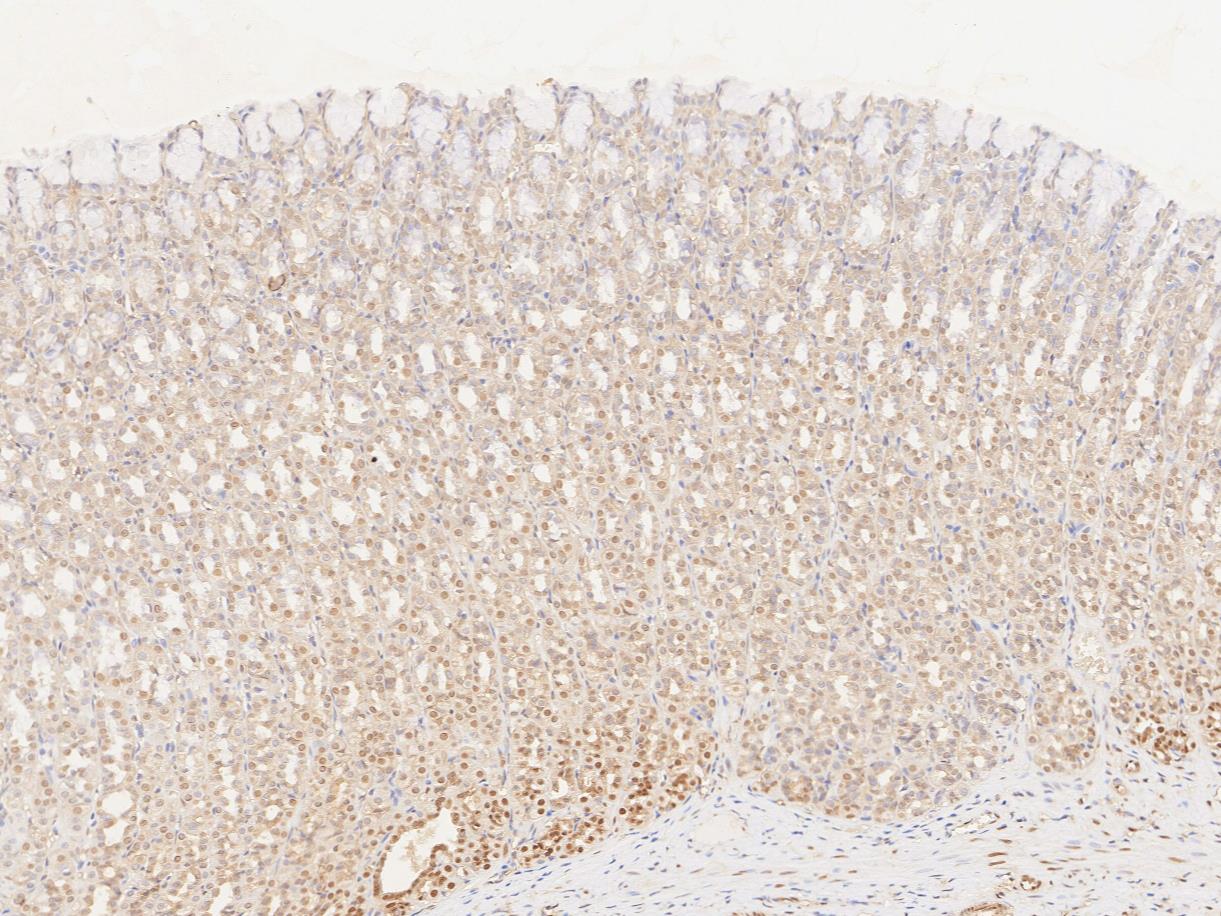

Supplement: Supplementary file 3 [file DataSheet1.zip › raw data/The images of Occludin, ZO-1, Claudin-4 and E-cadherin/E-cadherin_BL1.jpg]

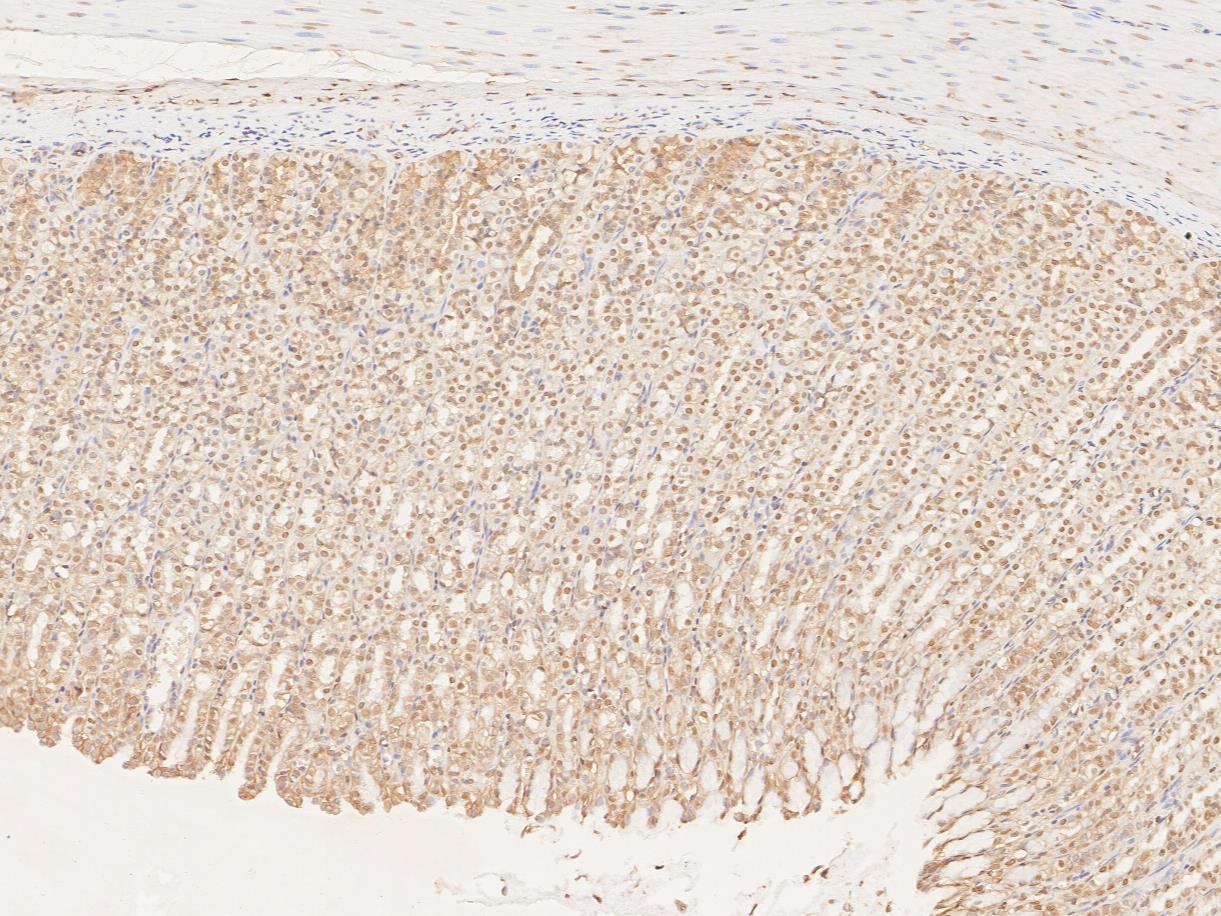

Supplement: Supplementary file 3 [file DataSheet1.zip › raw data/The images of Occludin, ZO-1, Claudin-4 and E-cadherin/E-cadherin_BL2.jpg]

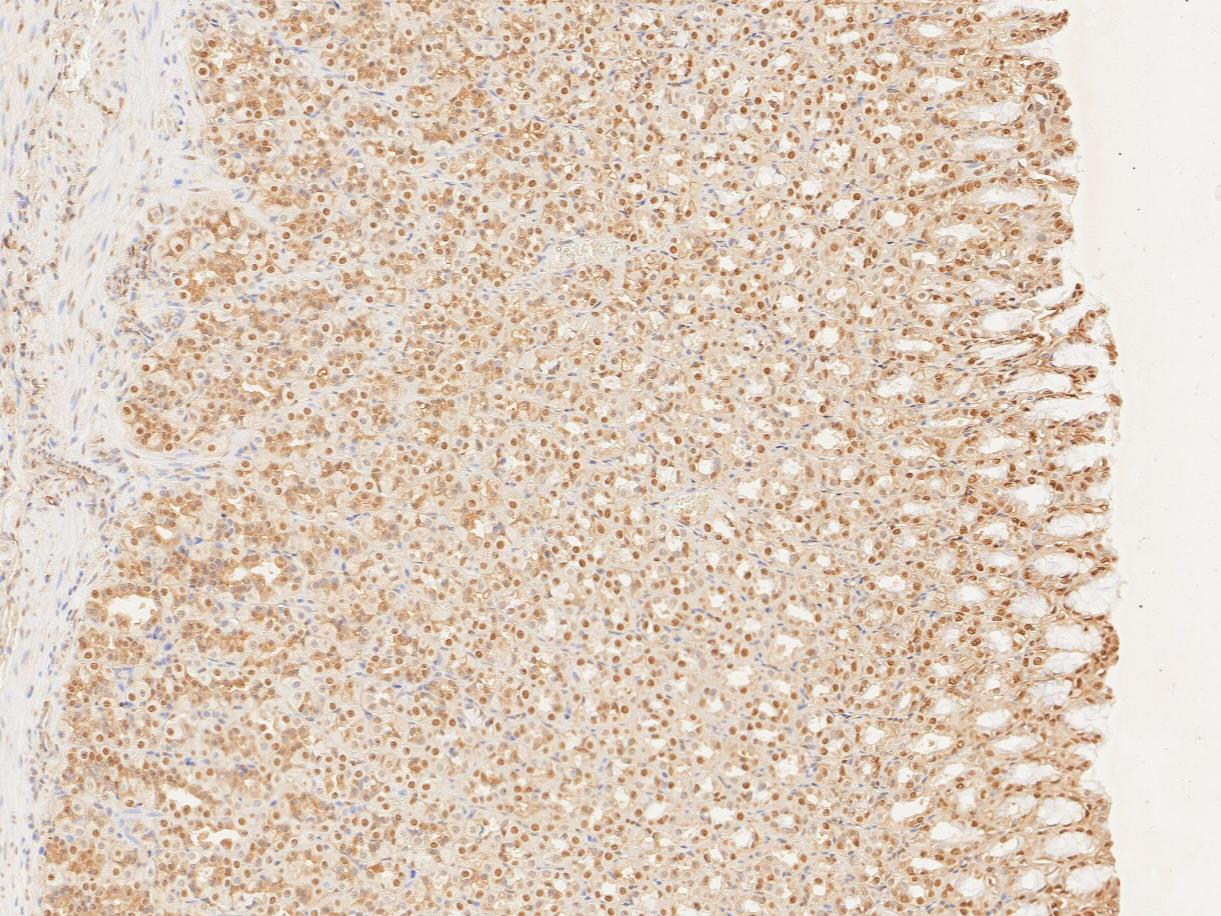

Supplement: Supplementary file 3 [file DataSheet1.zip › raw data/The images of Occludin, ZO-1, Claudin-4 and E-cadherin/E-cadherin_BL3.jpg]

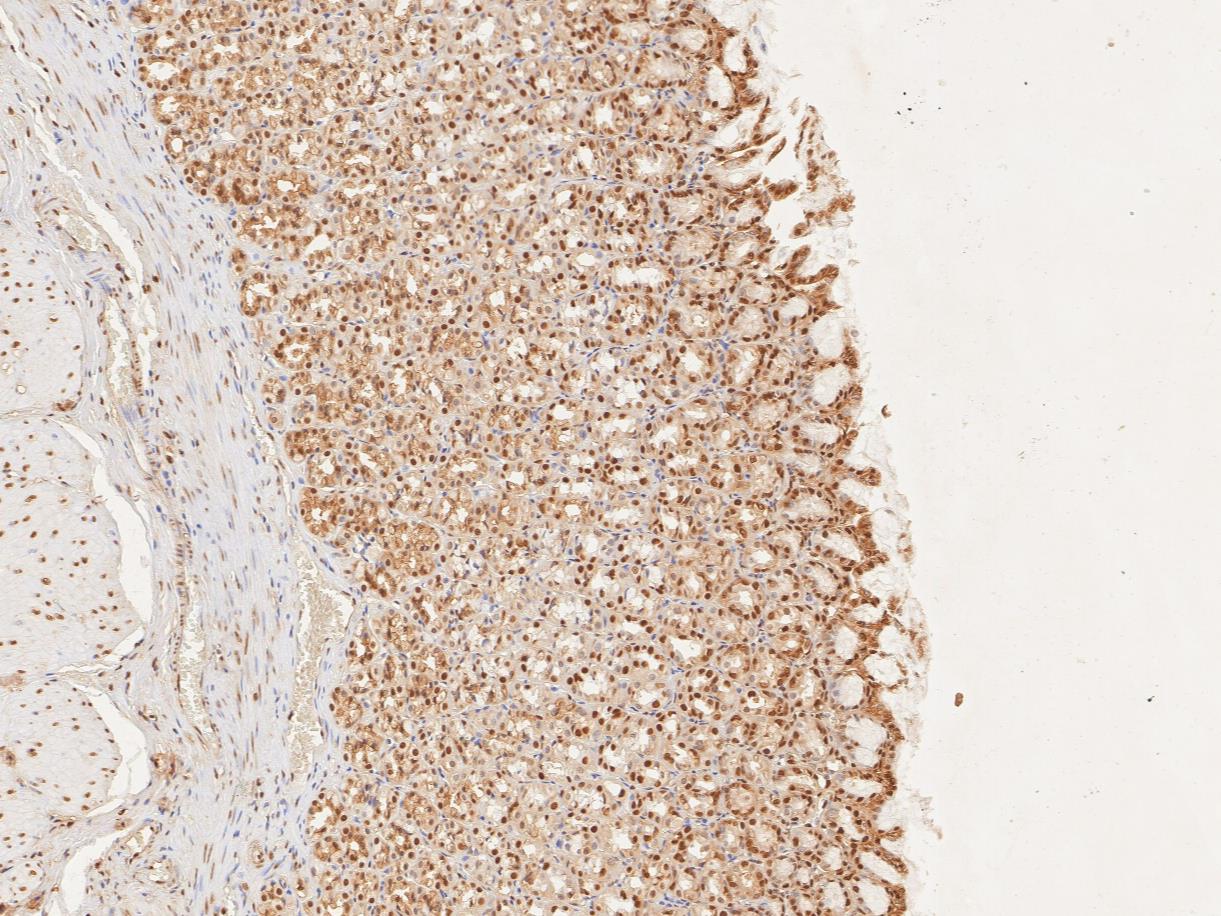

Supplement: Supplementary file 3 [file DataSheet1.zip › raw data/The images of Occludin, ZO-1, Claudin-4 and E-cadherin/E-cadherin_K1.jpg]

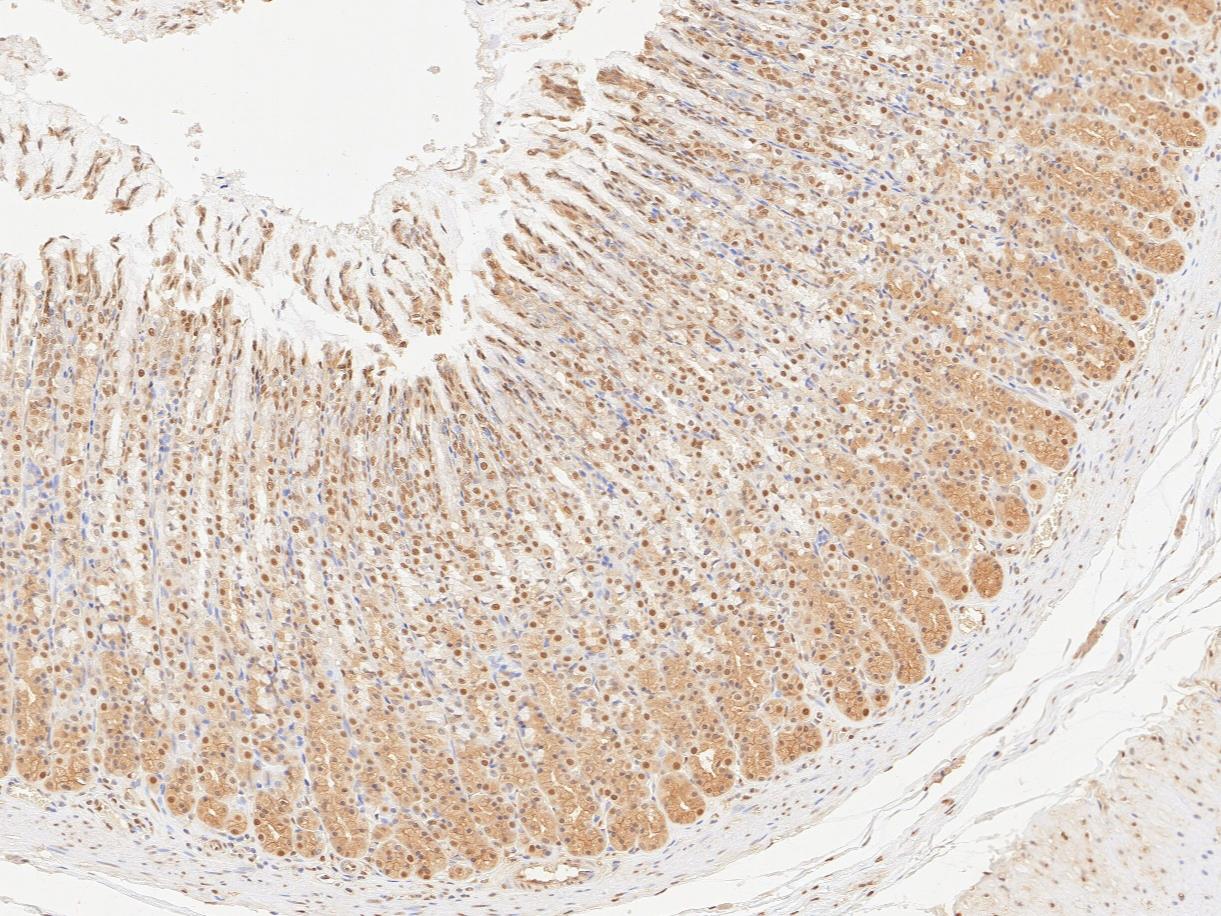

Supplement: Supplementary file 3 [file DataSheet1.zip › raw data/The images of Occludin, ZO-1, Claudin-4 and E-cadherin/E-cadherin_K2.jpg]

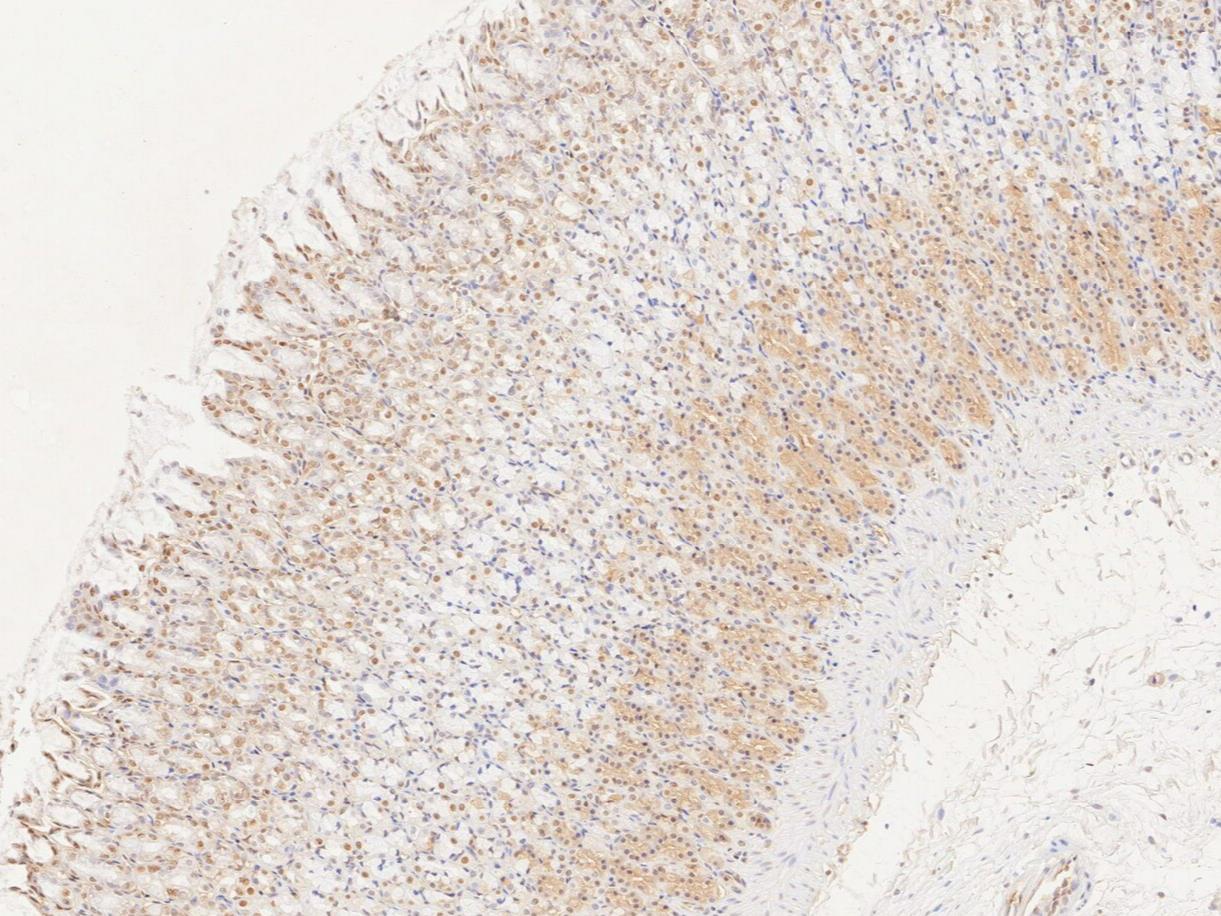

Supplement: Supplementary file 3 [file DataSheet1.zip › raw data/The images of Occludin, ZO-1, Claudin-4 and E-cadherin/E-cadherin_K3.jpg]

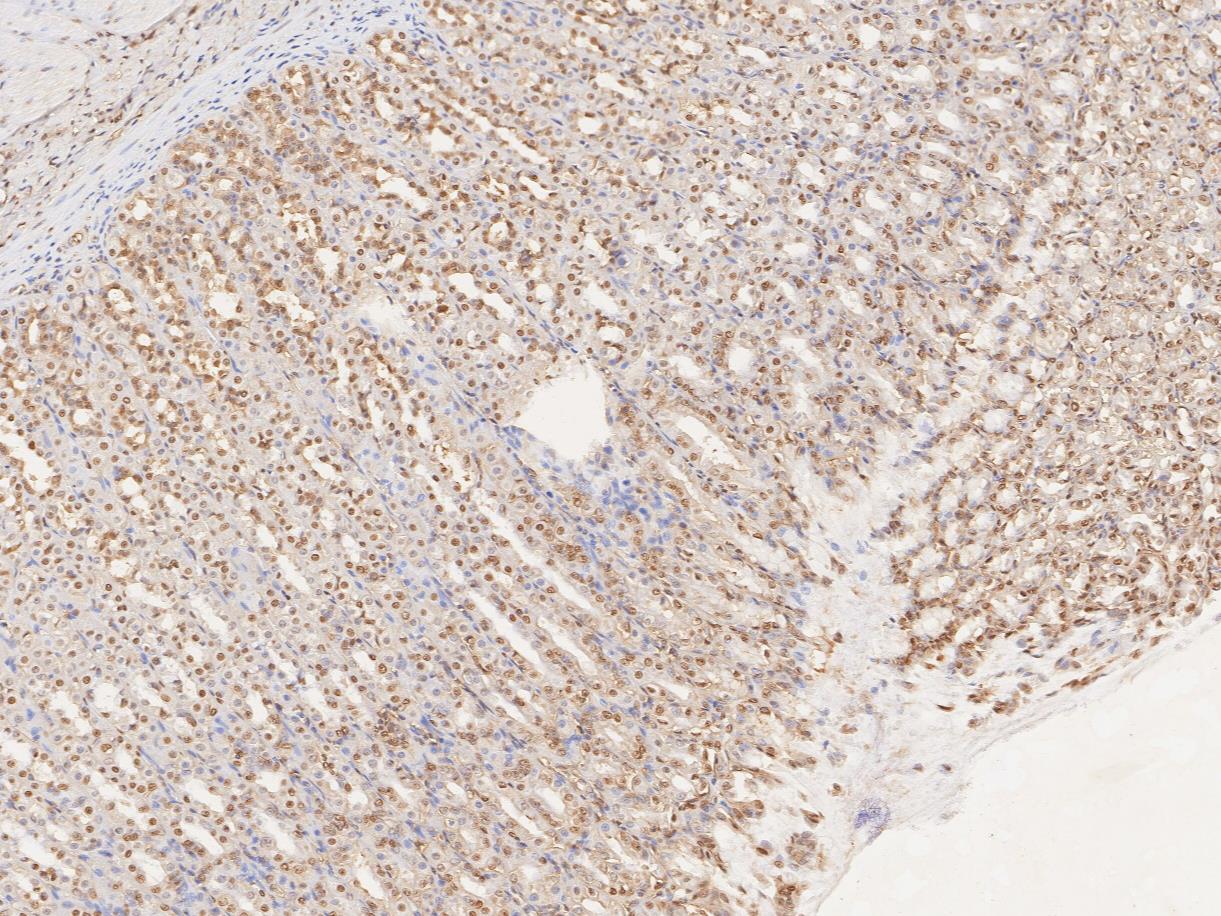

Supplement: Supplementary file 3 [file DataSheet1.zip › raw data/The images of Occludin, ZO-1, Claudin-4 and E-cadherin/E-cadherin_M1.jpg]

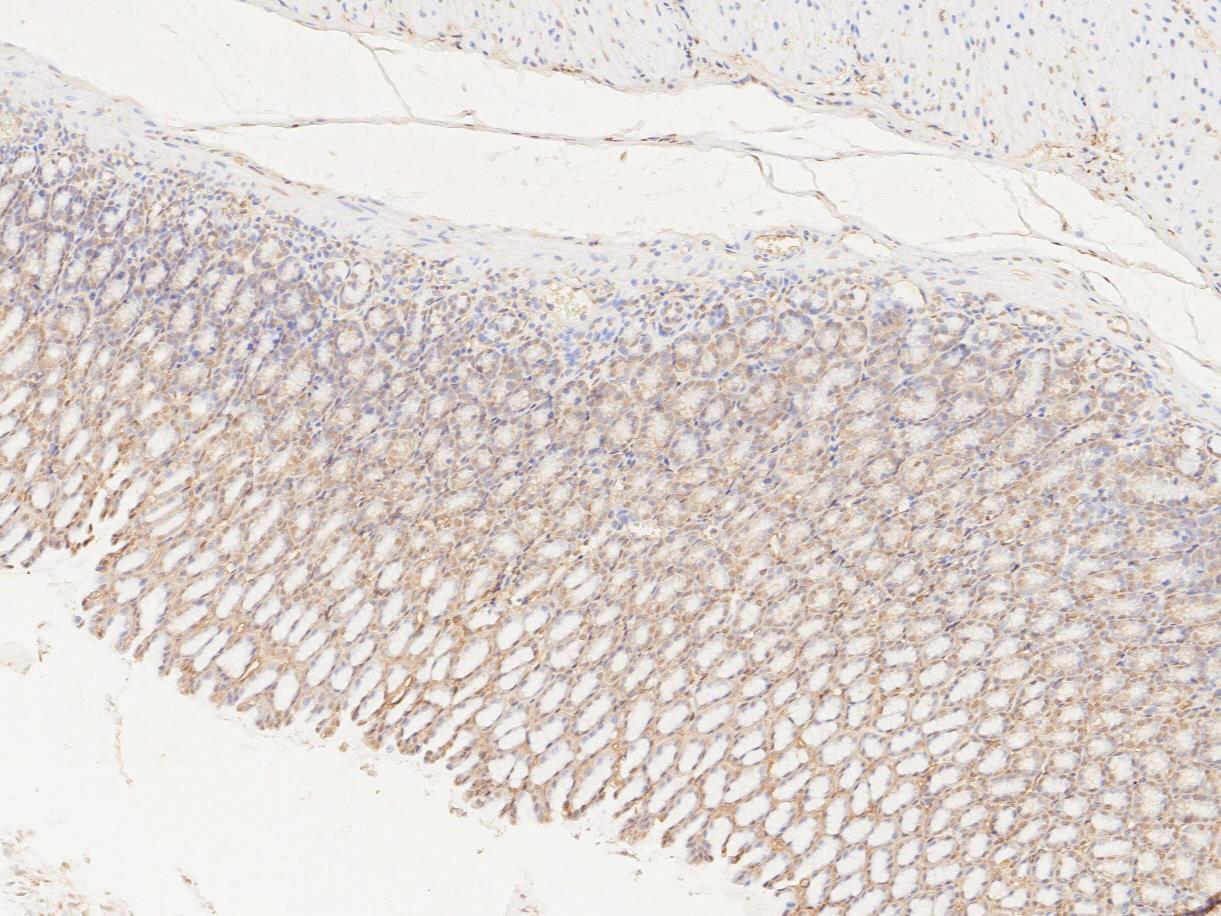

Supplement: Supplementary file 3 [file DataSheet1.zip › raw data/The images of Occludin, ZO-1, Claudin-4 and E-cadherin/E-cadherin_M2.jpg]

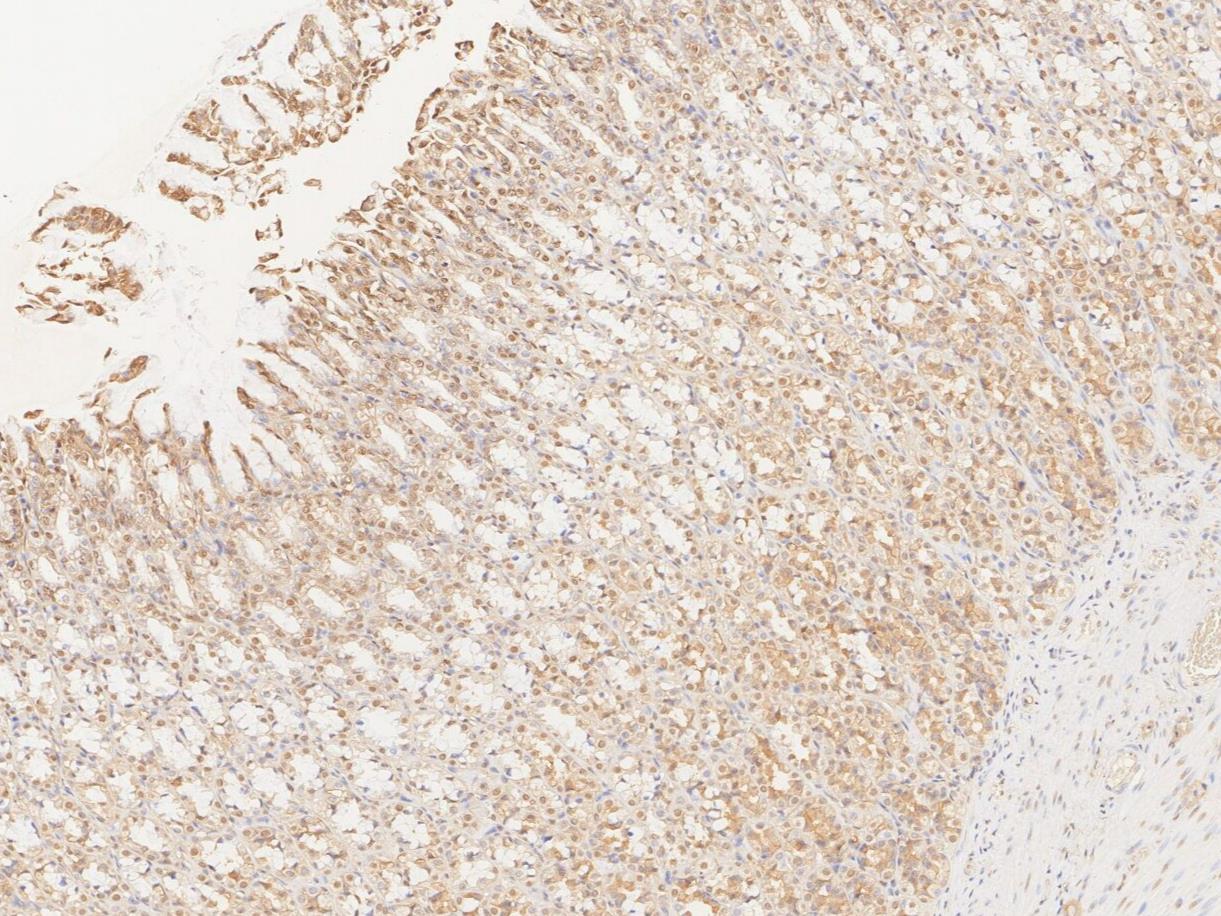

Supplement: Supplementary file 3 [file DataSheet1.zip › raw data/The images of Occludin, ZO-1, Claudin-4 and E-cadherin/E-cadherin_M3.jpg]

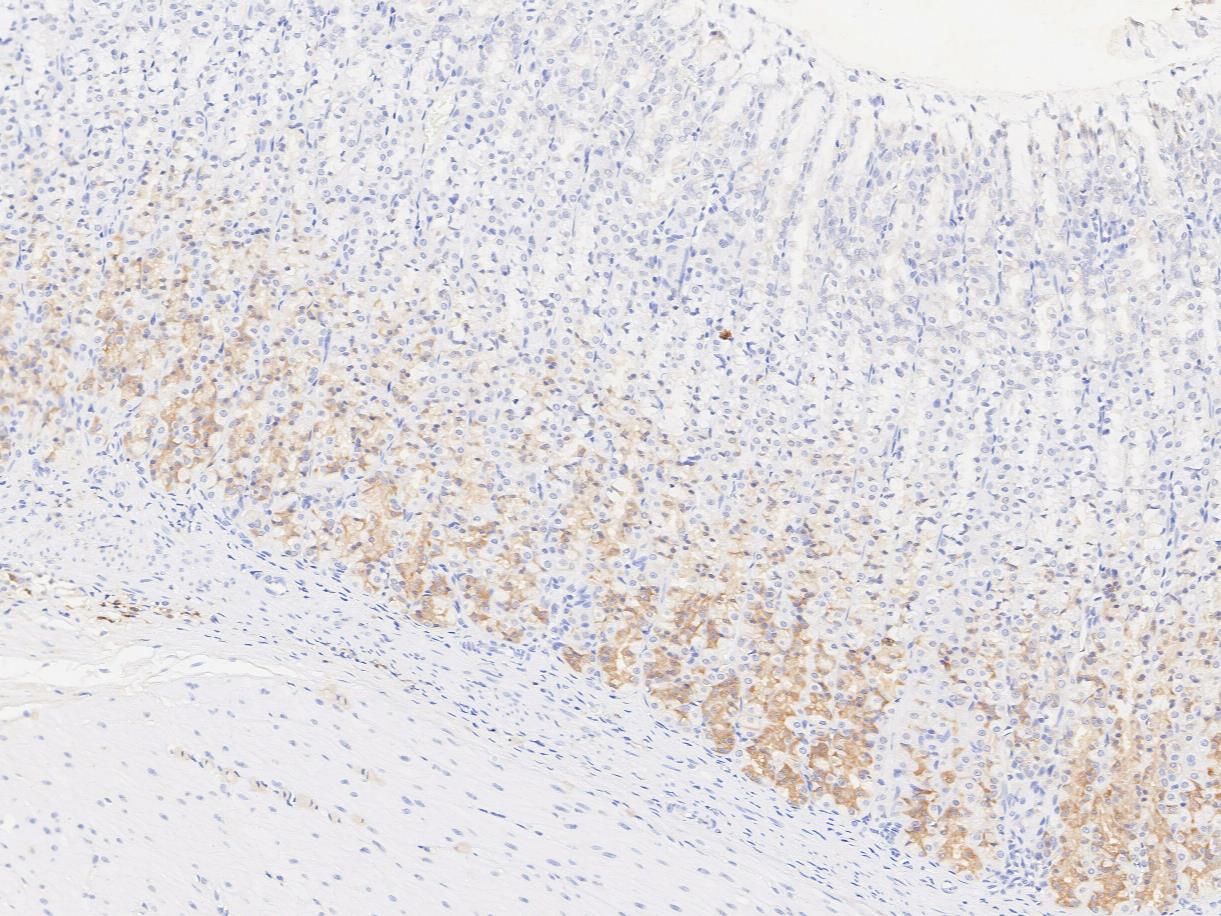

Supplement: Supplementary file 3 [file DataSheet1.zip › raw data/The images of Occludin, ZO-1, Claudin-4 and E-cadherin/Occludin_BH1.jpg]

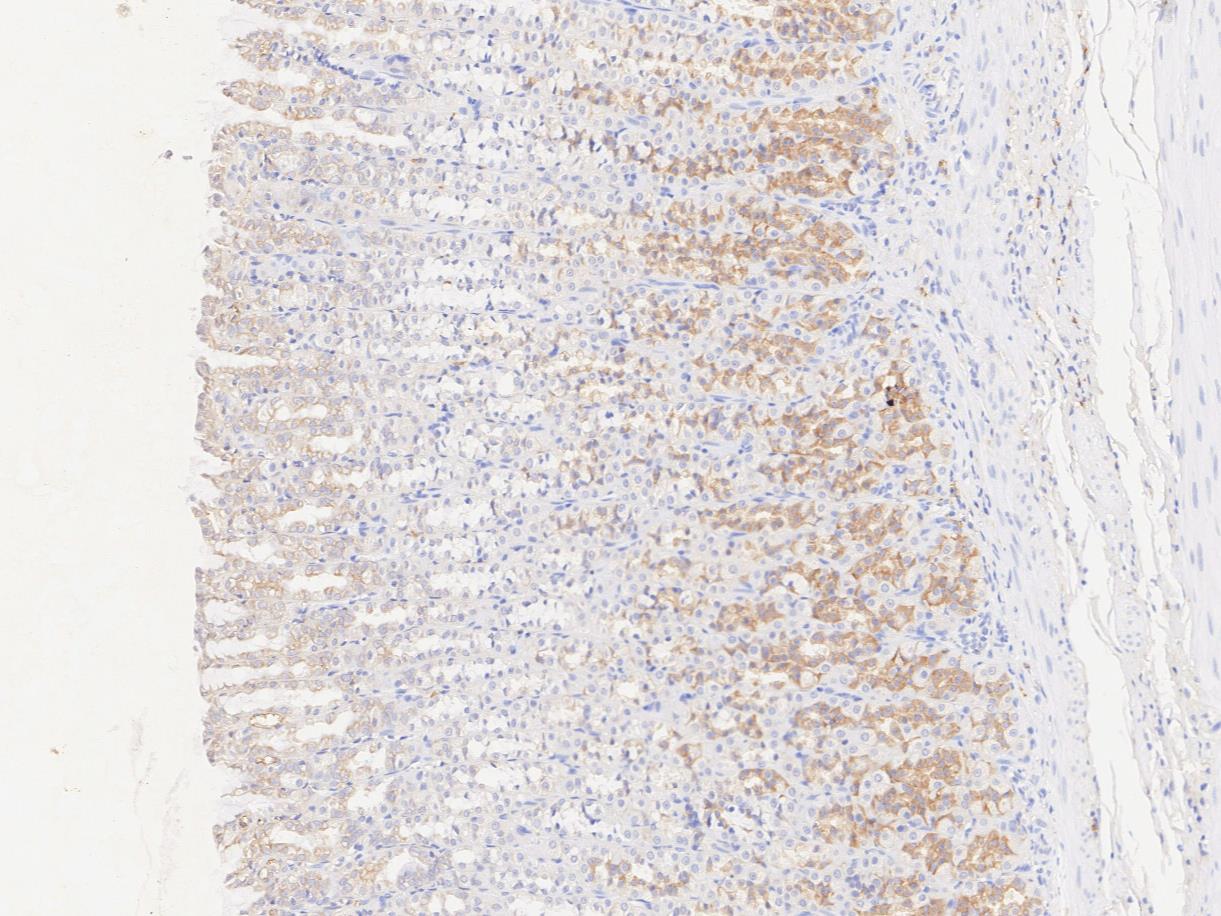

Supplement: Supplementary file 3 [file DataSheet1.zip › raw data/The images of Occludin, ZO-1, Claudin-4 and E-cadherin/Occludin_BH2.jpg]

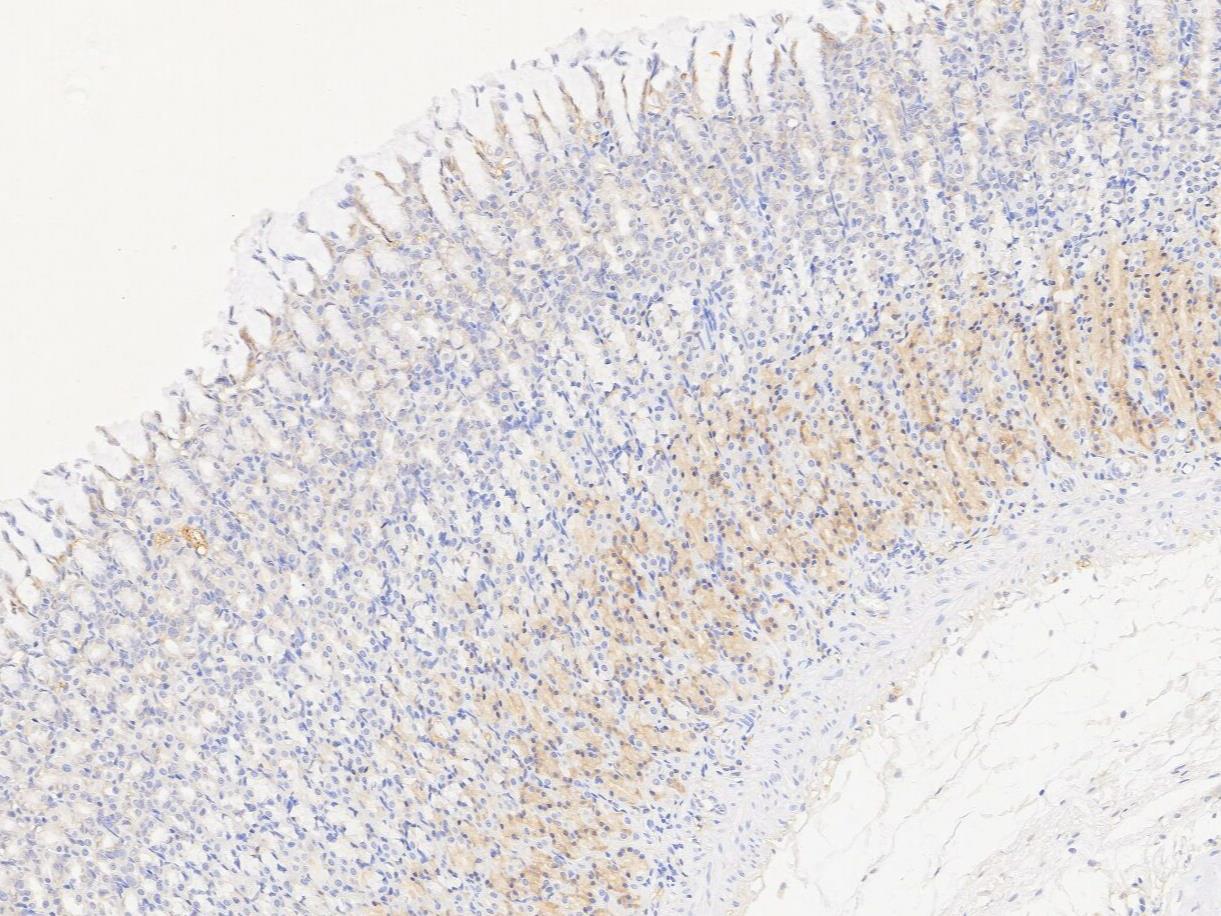

Supplement: Supplementary file 3 [file DataSheet1.zip › raw data/The images of Occludin, ZO-1, Claudin-4 and E-cadherin/Occludin_BH3.jpg]

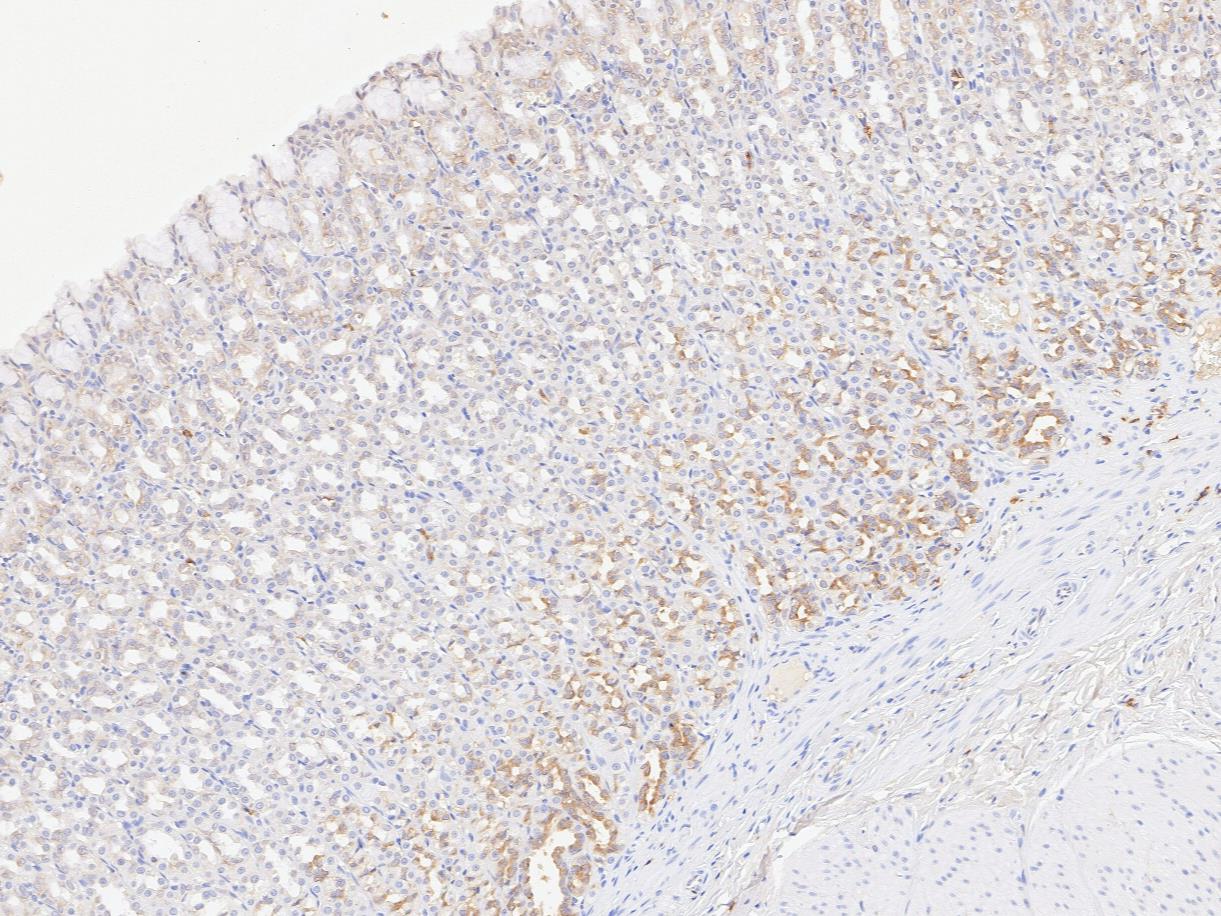

Supplement: Supplementary file 3 [file DataSheet1.zip › raw data/The images of Occludin, ZO-1, Claudin-4 and E-cadherin/Occludin_BL1.jpg]

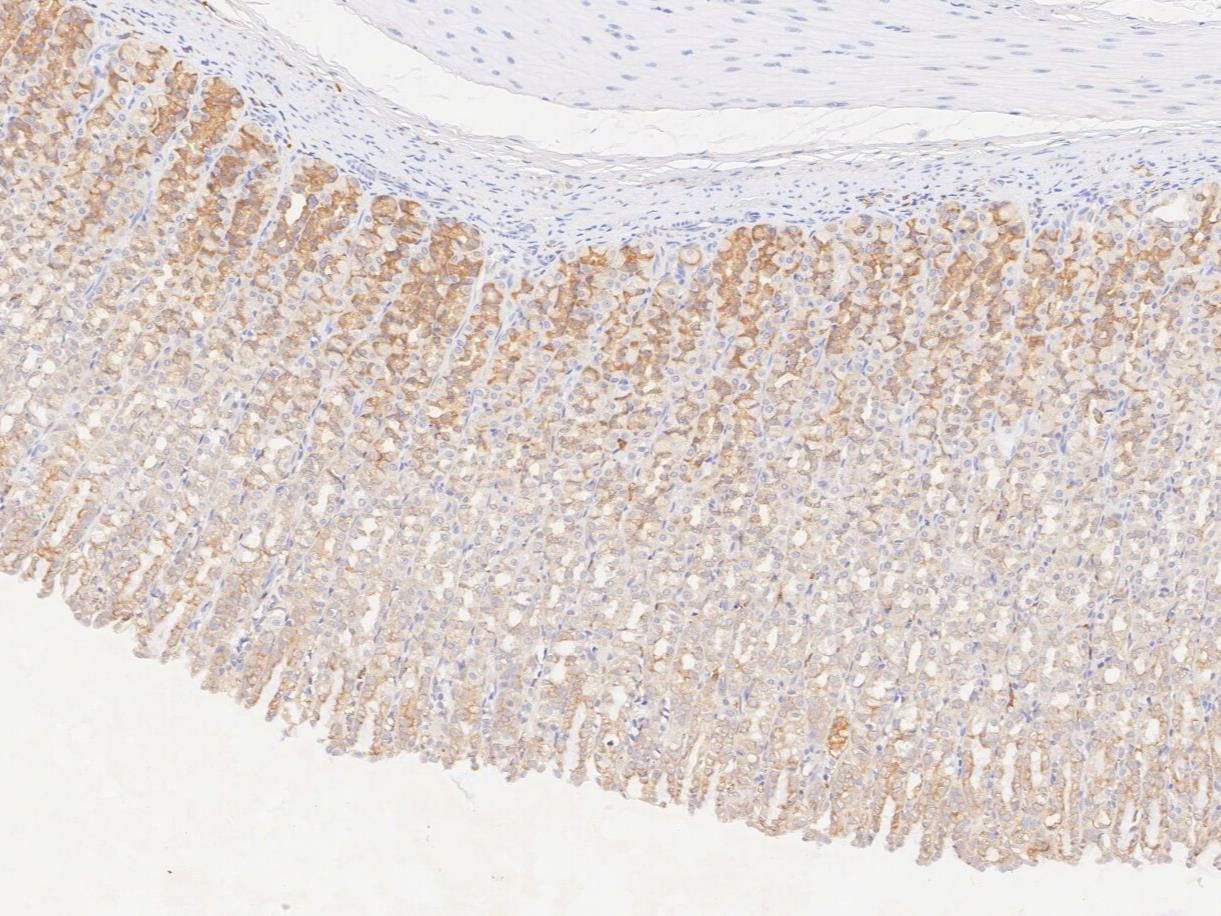

Supplement: Supplementary file 3 [file DataSheet1.zip › raw data/The images of Occludin, ZO-1, Claudin-4 and E-cadherin/Occludin_BL2.jpg]

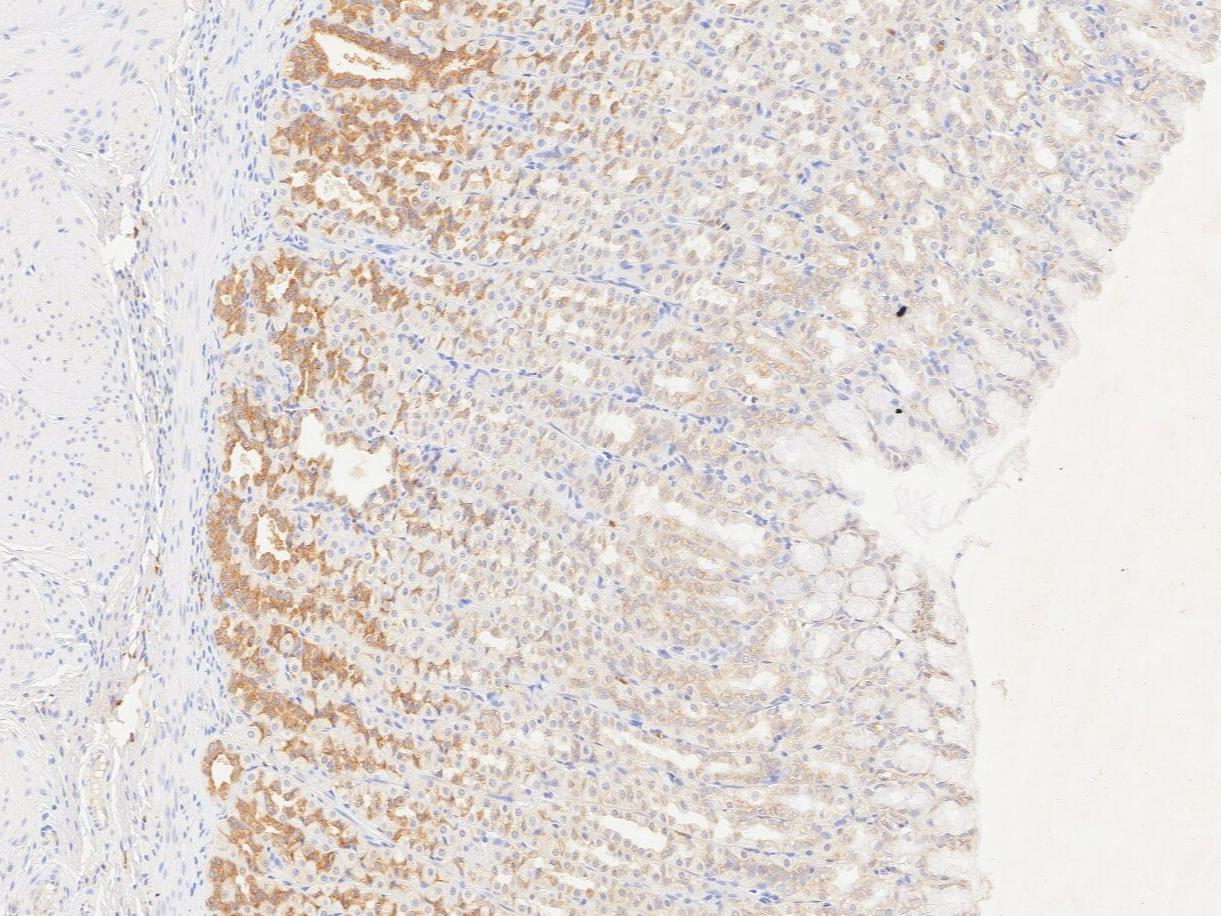

Supplement: Supplementary file 3 [file DataSheet1.zip › raw data/The images of Occludin, ZO-1, Claudin-4 and E-cadherin/Occludin_BL3.jpg]

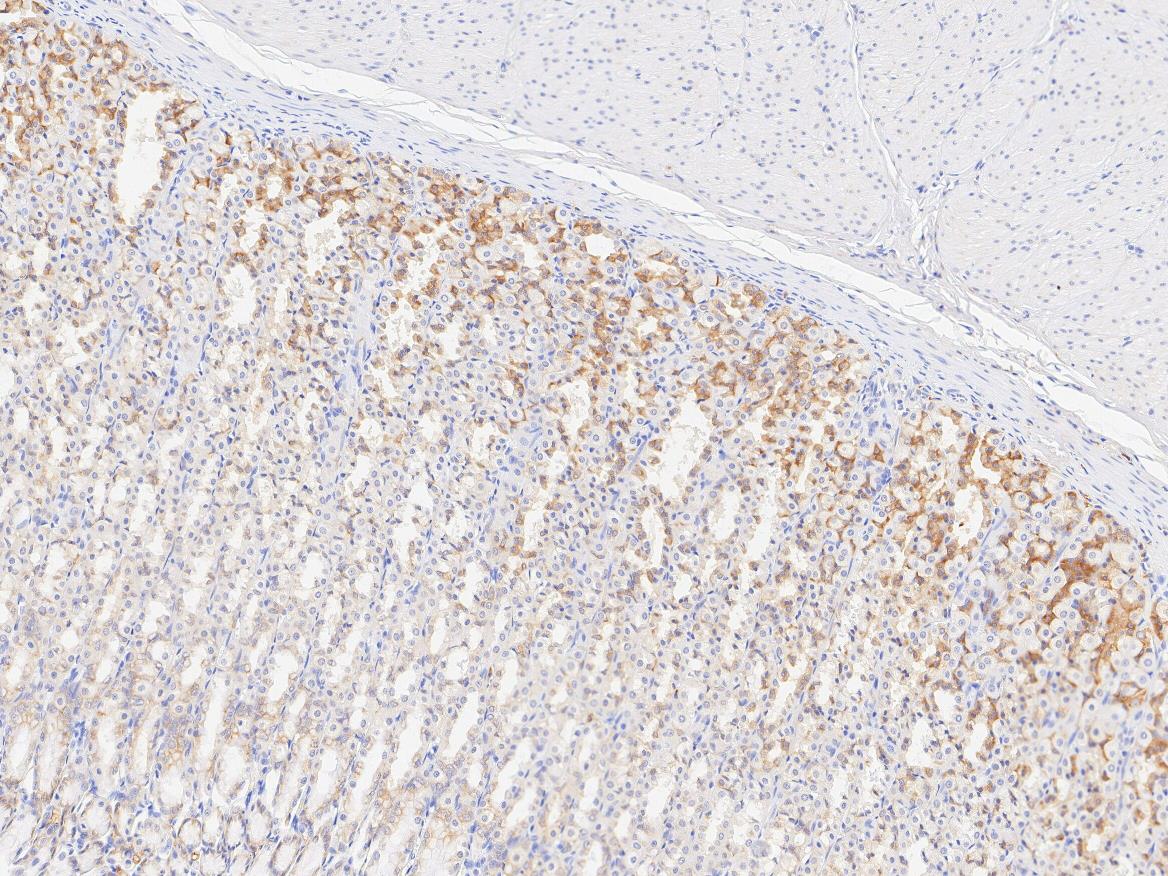

Supplement: Supplementary file 3 [file DataSheet1.zip › raw data/The images of Occludin, ZO-1, Claudin-4 and E-cadherin/Occludin_K1.jpg]

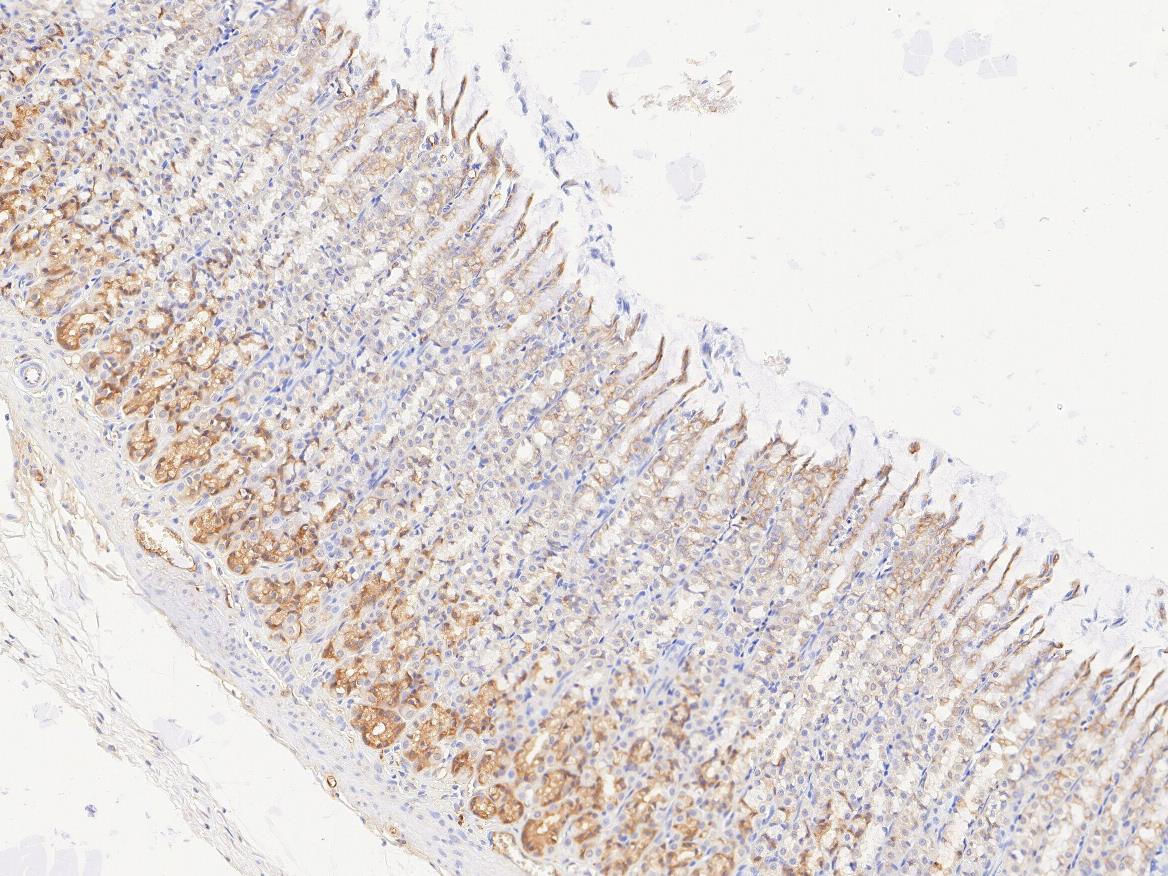

Supplement: Supplementary file 3 [file DataSheet1.zip › raw data/The images of Occludin, ZO-1, Claudin-4 and E-cadherin/Occludin_K2.jpg]

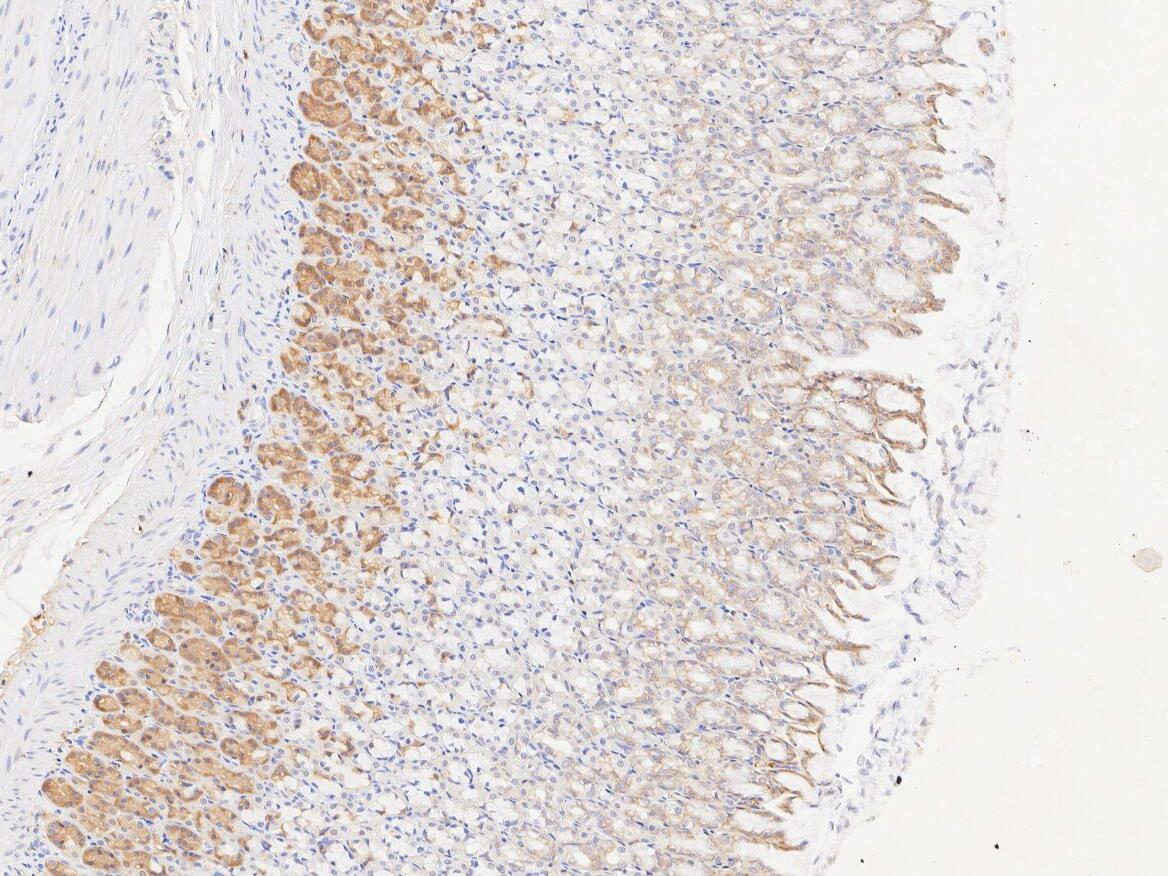

Supplement: Supplementary file 3 [file DataSheet1.zip › raw data/The images of Occludin, ZO-1, Claudin-4 and E-cadherin/Occludin_K3.jpg]

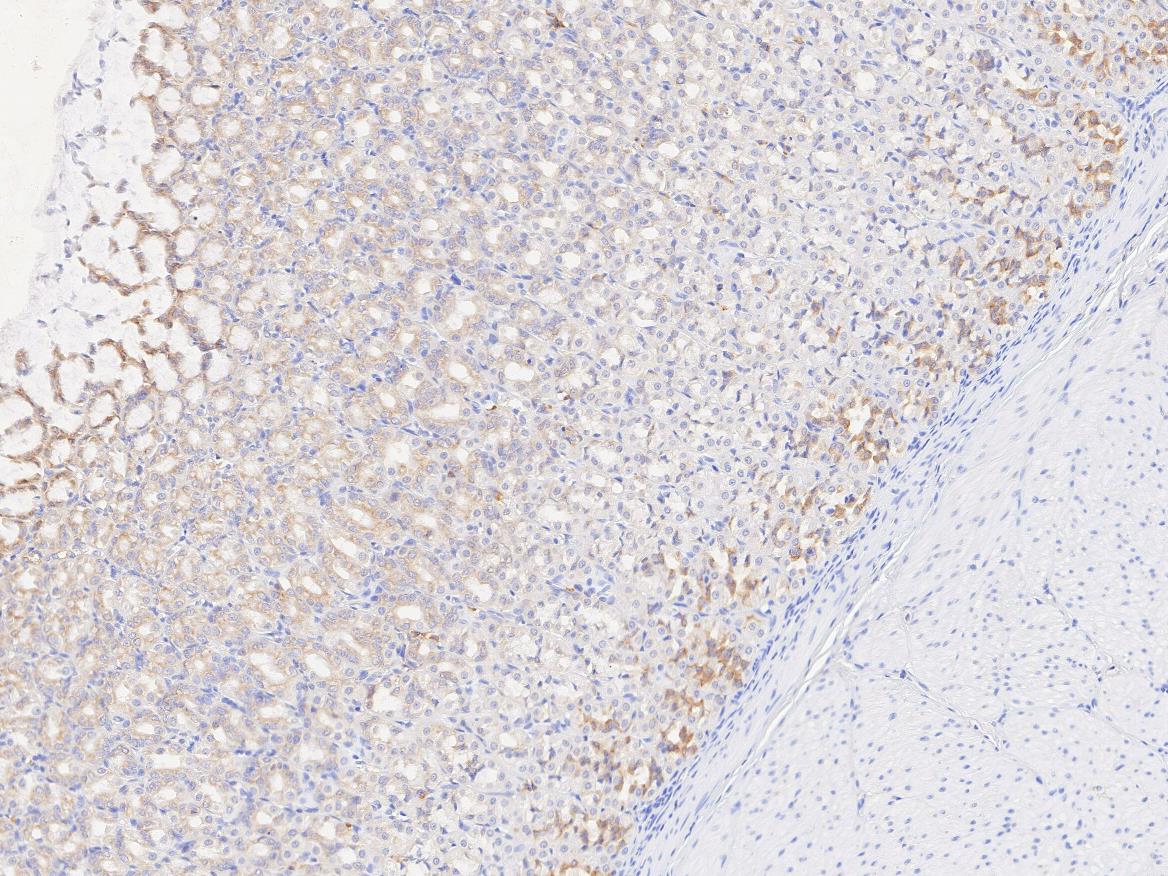

Supplement: Supplementary file 3 [file DataSheet1.zip › raw data/The images of Occludin, ZO-1, Claudin-4 and E-cadherin/Occludin_M1.jpg]

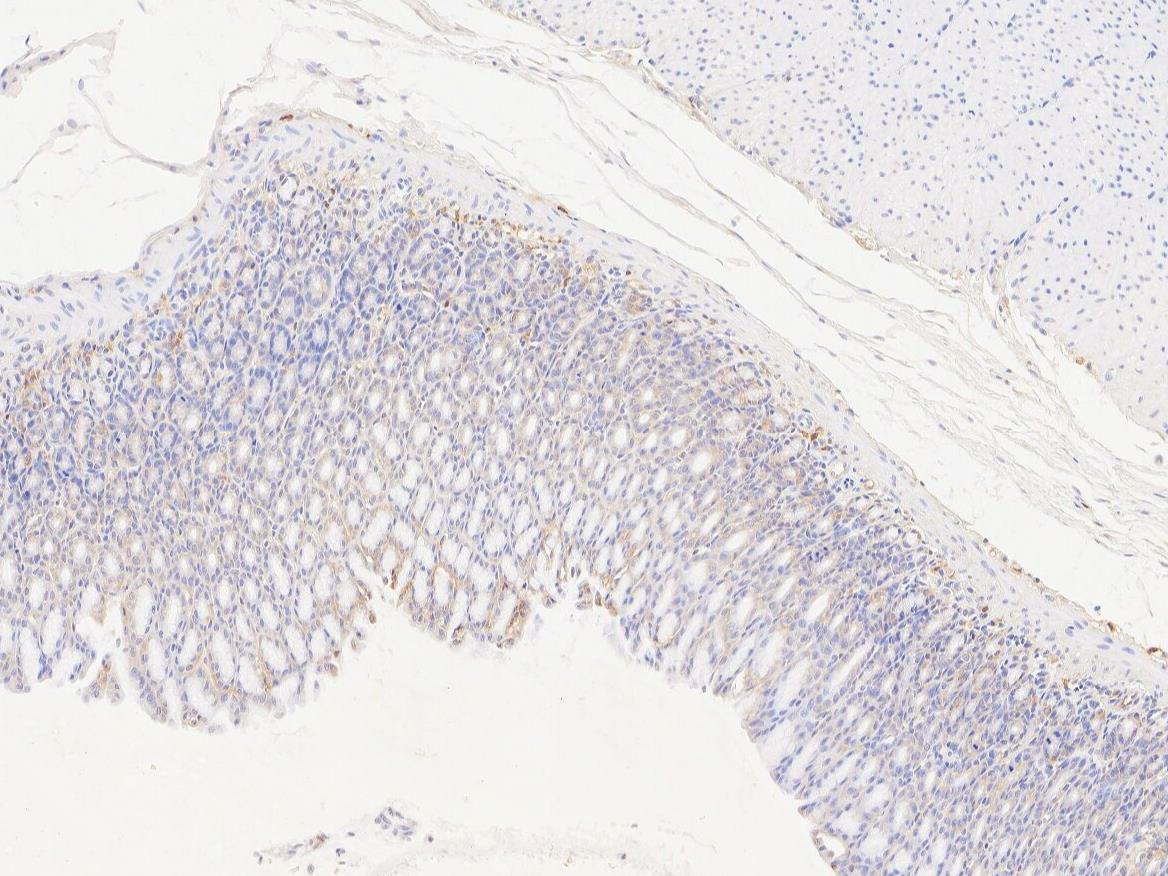

Supplement: Supplementary file 3 [file DataSheet1.zip › raw data/The images of Occludin, ZO-1, Claudin-4 and E-cadherin/Occludin_M2.jpg]

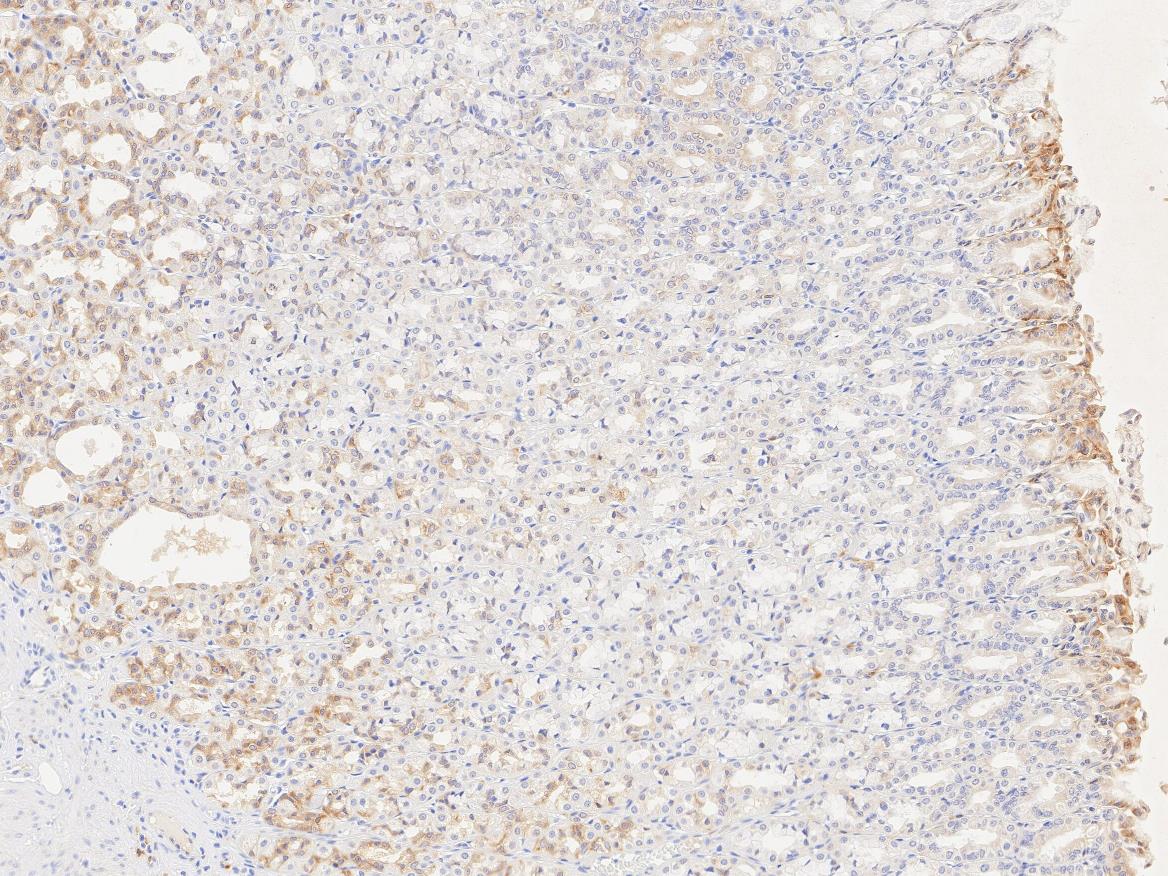

Supplement: Supplementary file 3 [file DataSheet1.zip › raw data/The images of Occludin, ZO-1, Claudin-4 and E-cadherin/Occludin_M3.jpg]

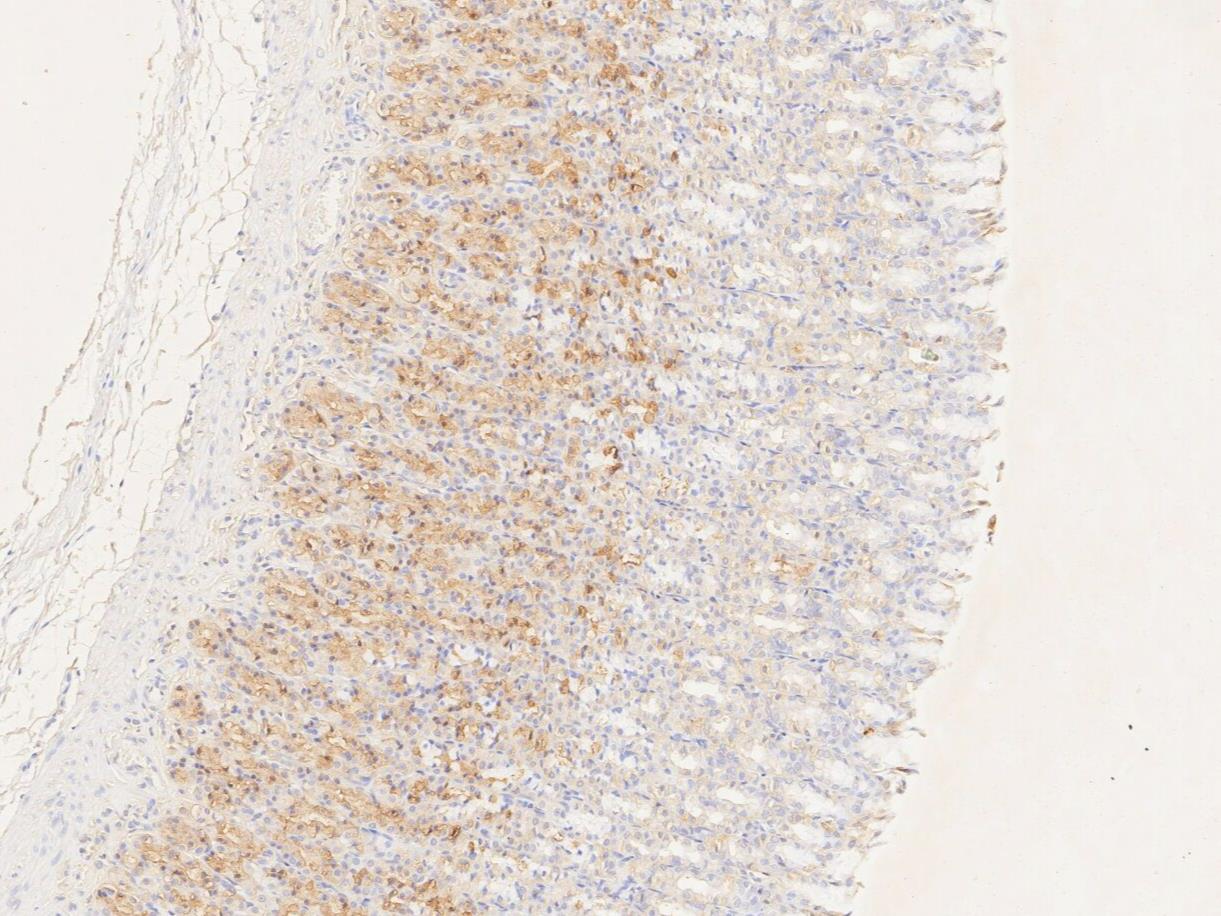

Supplement: Supplementary file 3 [file DataSheet1.zip › raw data/The images of Occludin, ZO-1, Claudin-4 and E-cadherin/ZO-1_BH1.jpg]

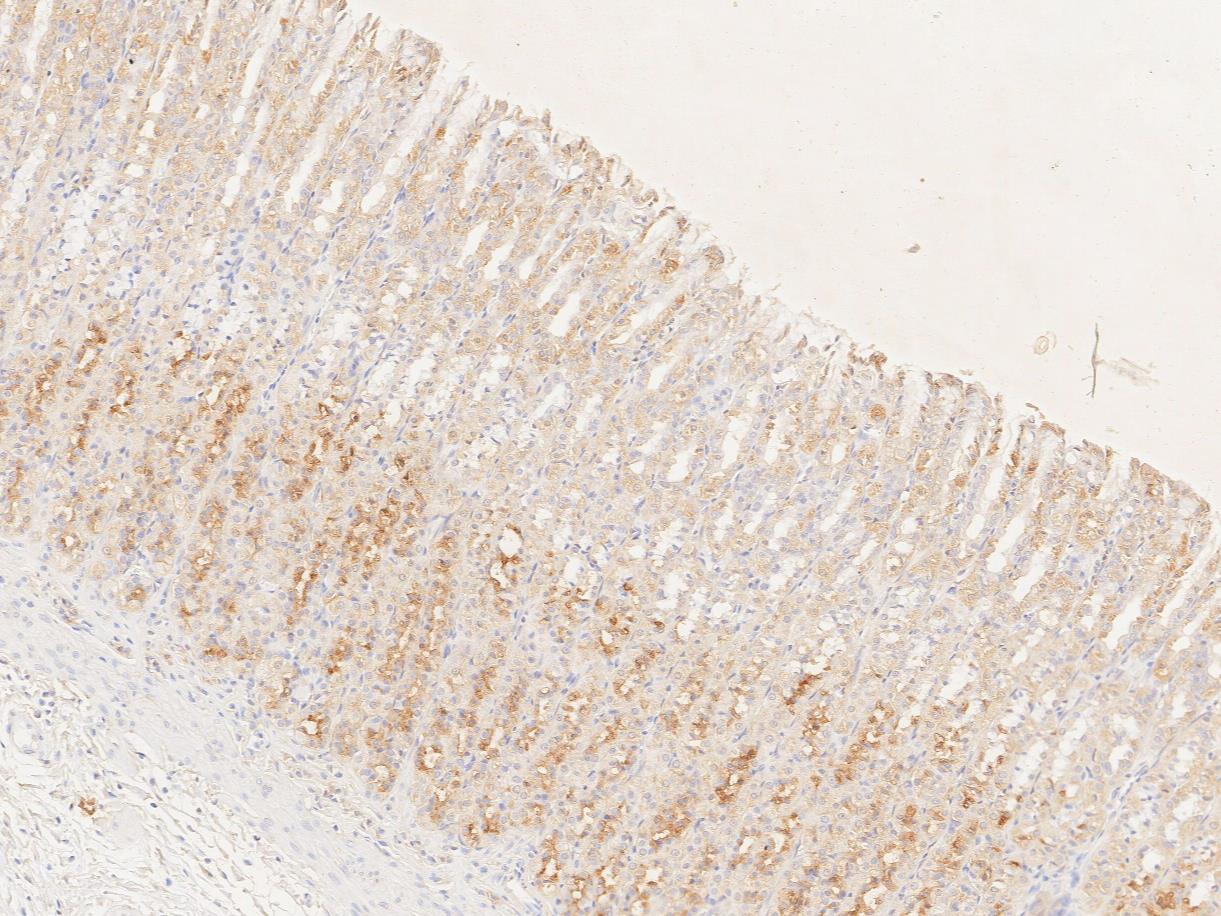

Supplement: Supplementary file 3 [file DataSheet1.zip › raw data/The images of Occludin, ZO-1, Claudin-4 and E-cadherin/ZO-1_BH2.jpg]

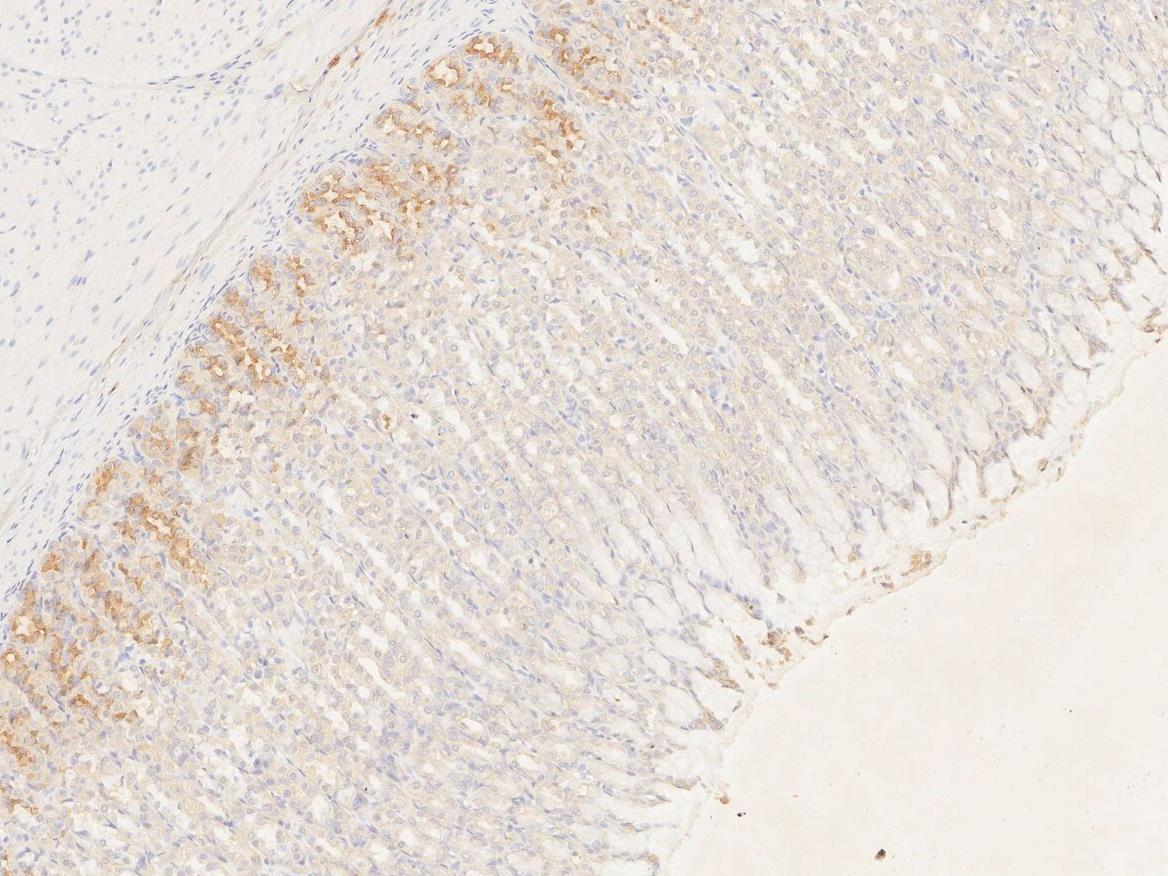

Supplement: Supplementary file 3 [file DataSheet1.zip › raw data/The images of Occludin, ZO-1, Claudin-4 and E-cadherin/ZO-1_BH3.jpg]

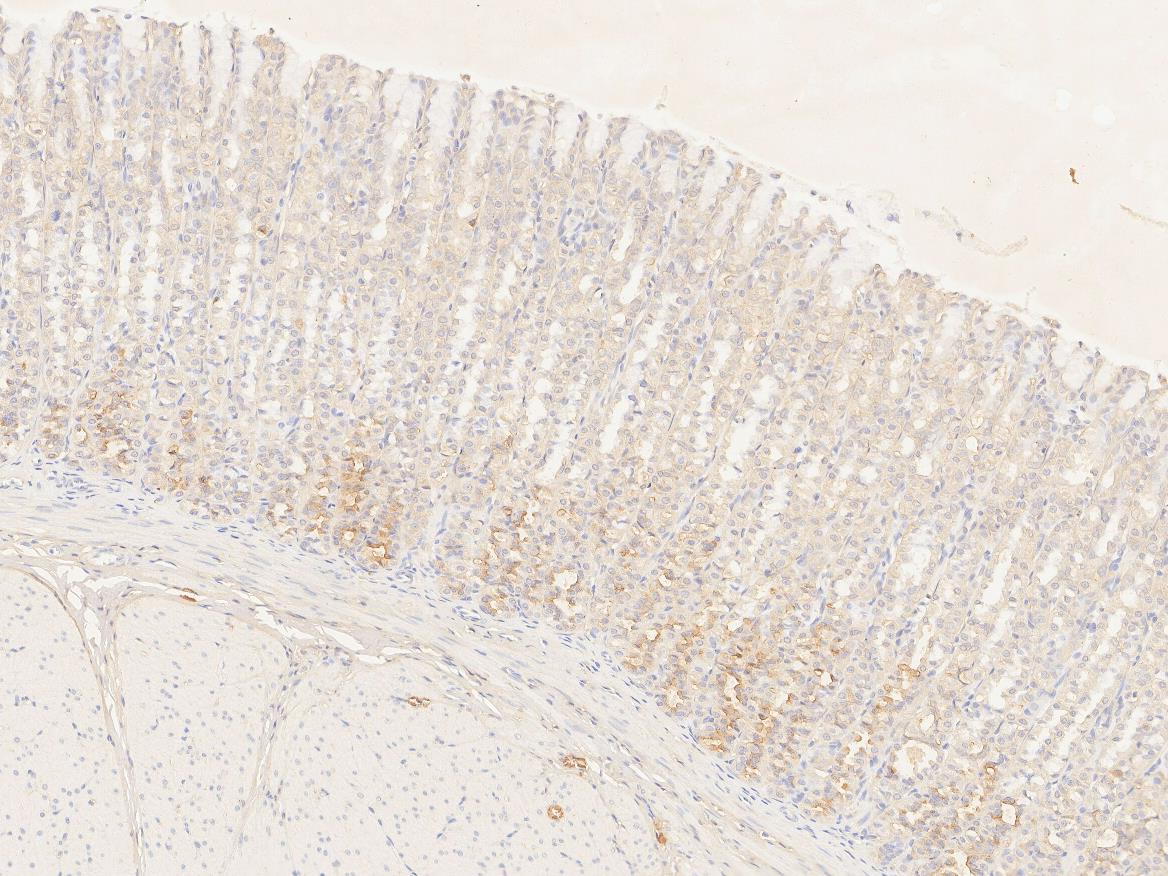

Supplement: Supplementary file 3 [file DataSheet1.zip › raw data/The images of Occludin, ZO-1, Claudin-4 and E-cadherin/ZO-1_BL1.jpg]

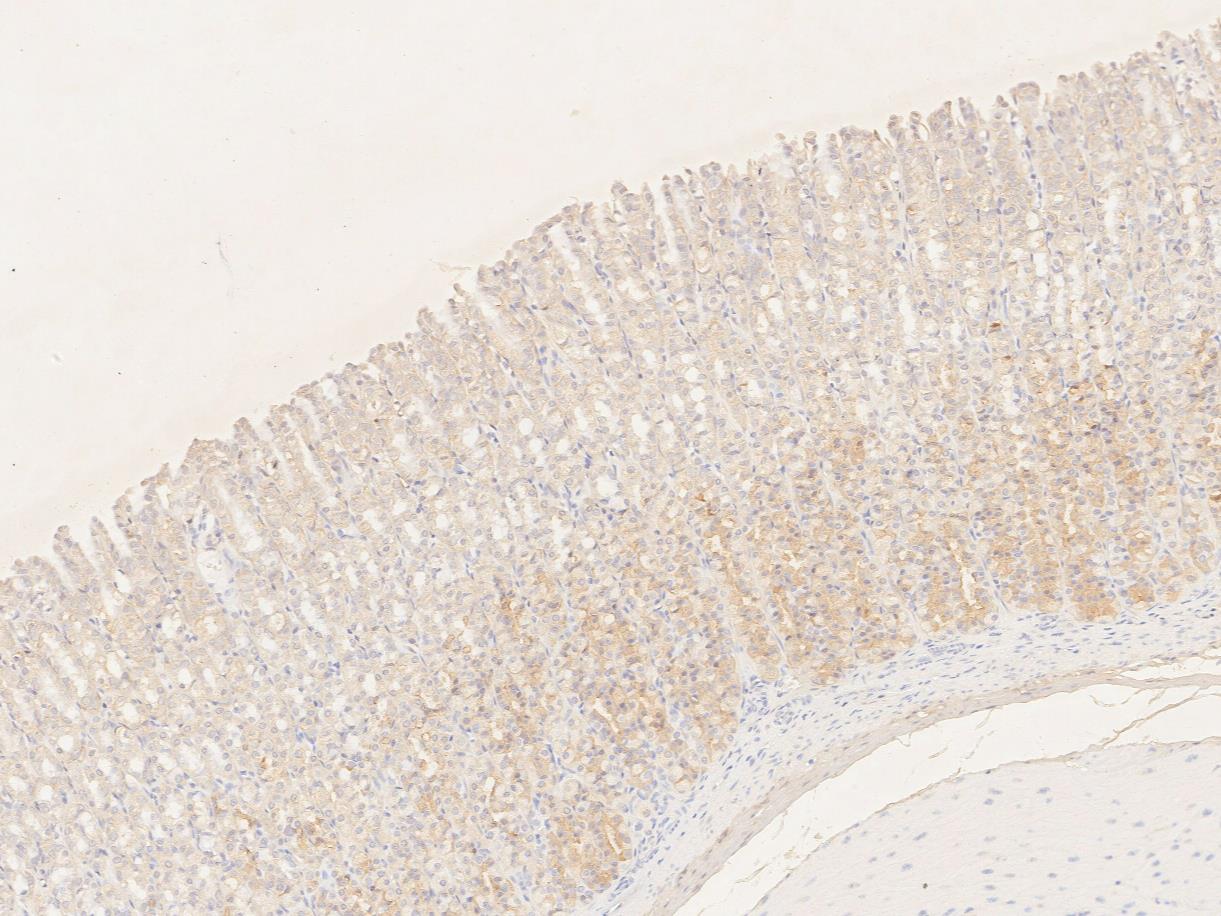

Supplement: Supplementary file 3 [file DataSheet1.zip › raw data/The images of Occludin, ZO-1, Claudin-4 and E-cadherin/ZO-1_BL2.jpg]

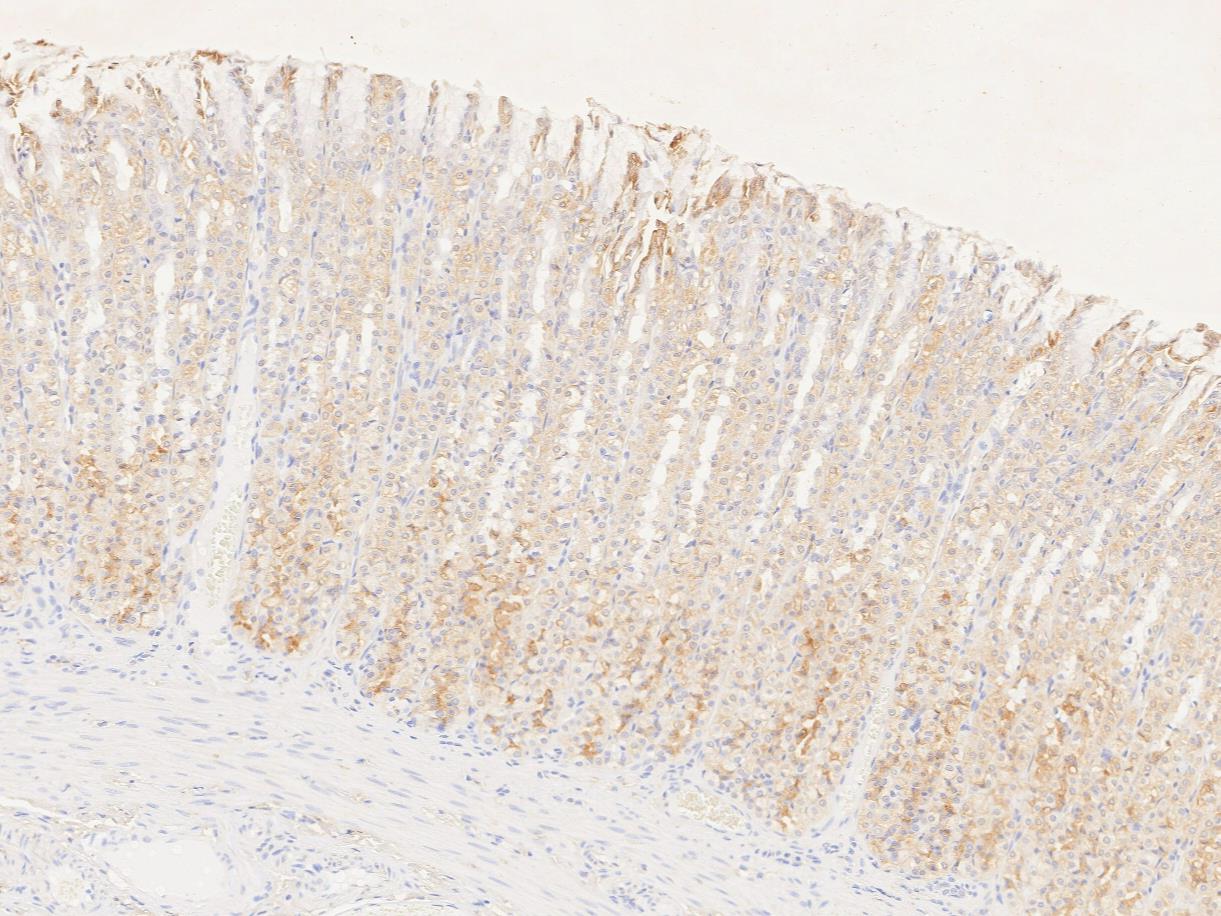

Supplement: Supplementary file 3 [file DataSheet1.zip › raw data/The images of Occludin, ZO-1, Claudin-4 and E-cadherin/ZO-1_BL3.jpg]

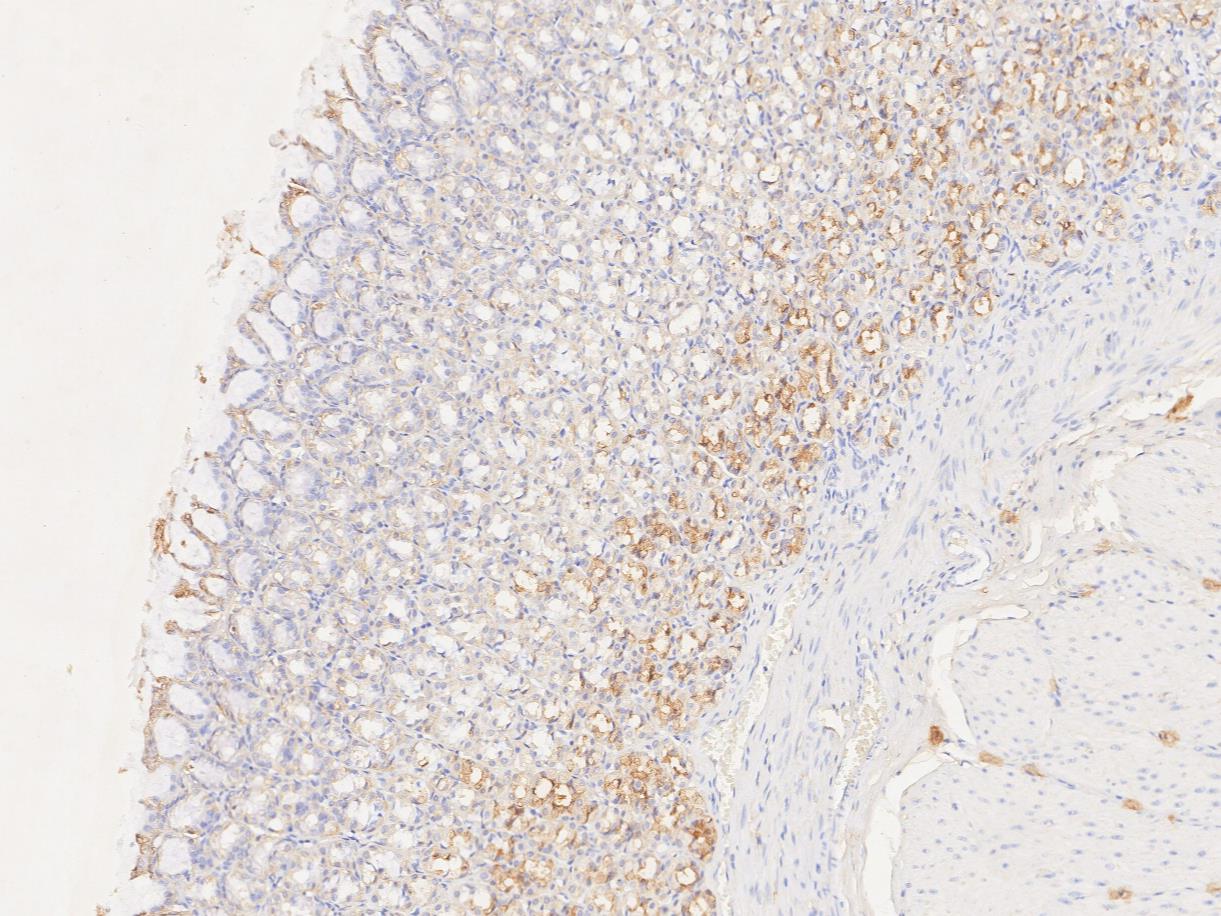

Supplement: Supplementary file 3 [file DataSheet1.zip › raw data/The images of Occludin, ZO-1, Claudin-4 and E-cadherin/ZO-1_K1.jpg]

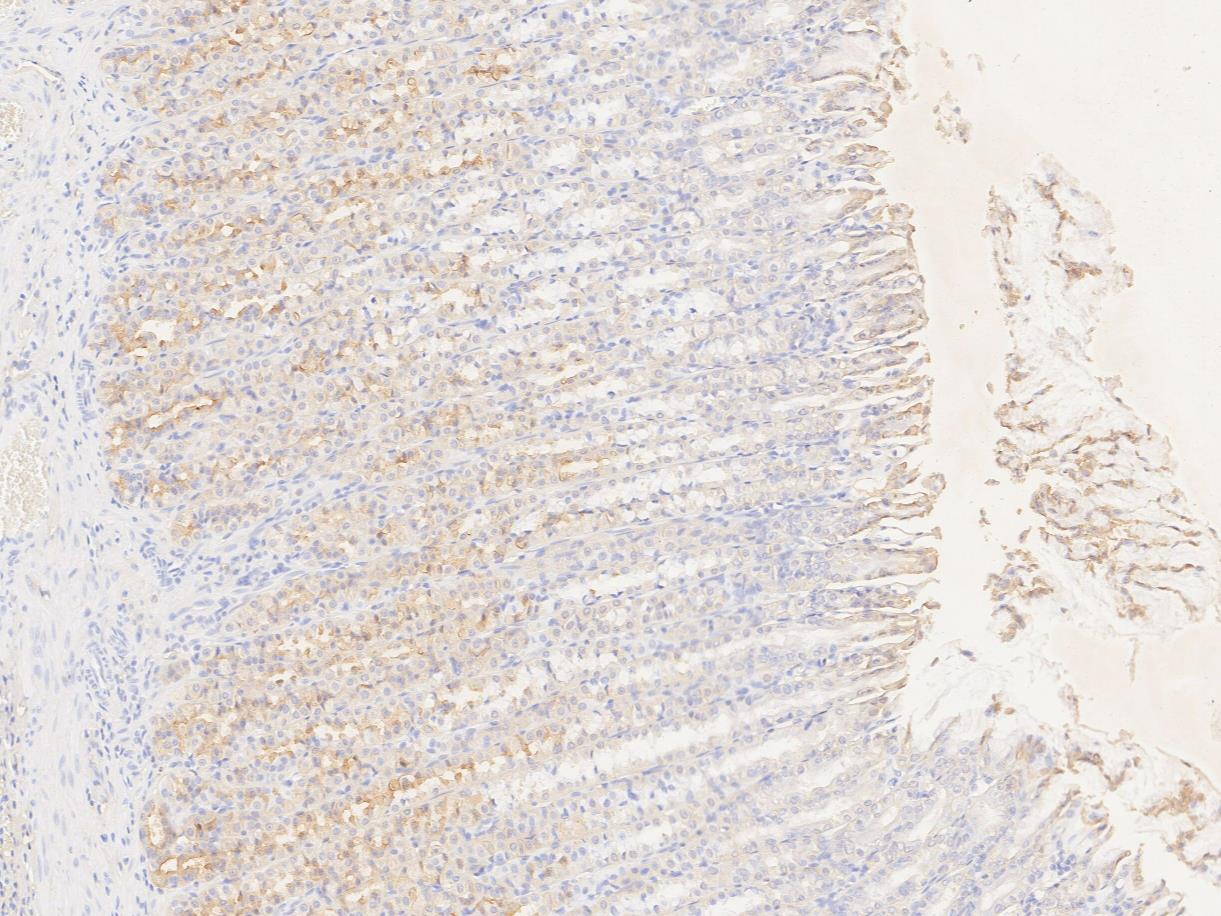

Supplement: Supplementary file 3 [file DataSheet1.zip › raw data/The images of Occludin, ZO-1, Claudin-4 and E-cadherin/ZO-1_K2.jpg]

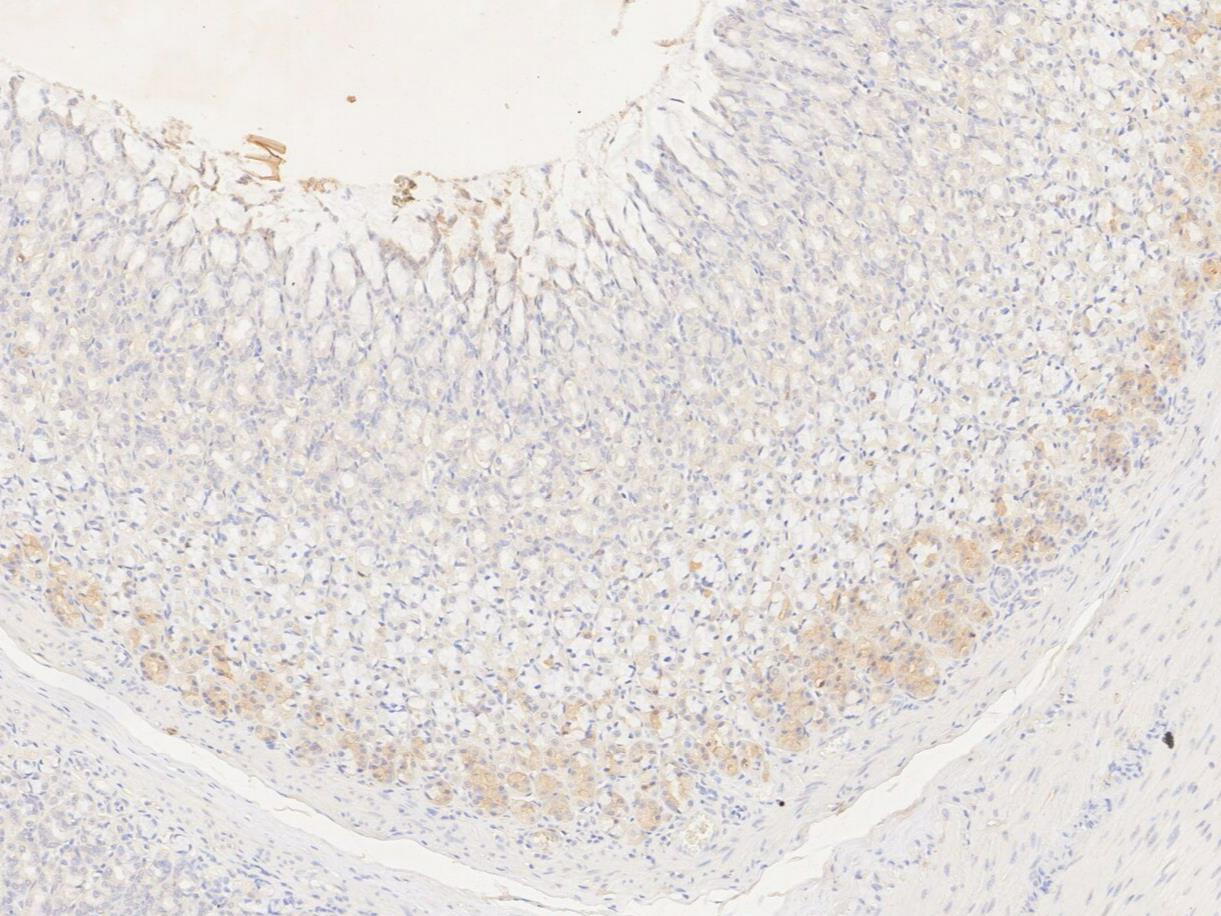

Supplement: Supplementary file 3 [file DataSheet1.zip › raw data/The images of Occludin, ZO-1, Claudin-4 and E-cadherin/ZO-1_K3.jpg]

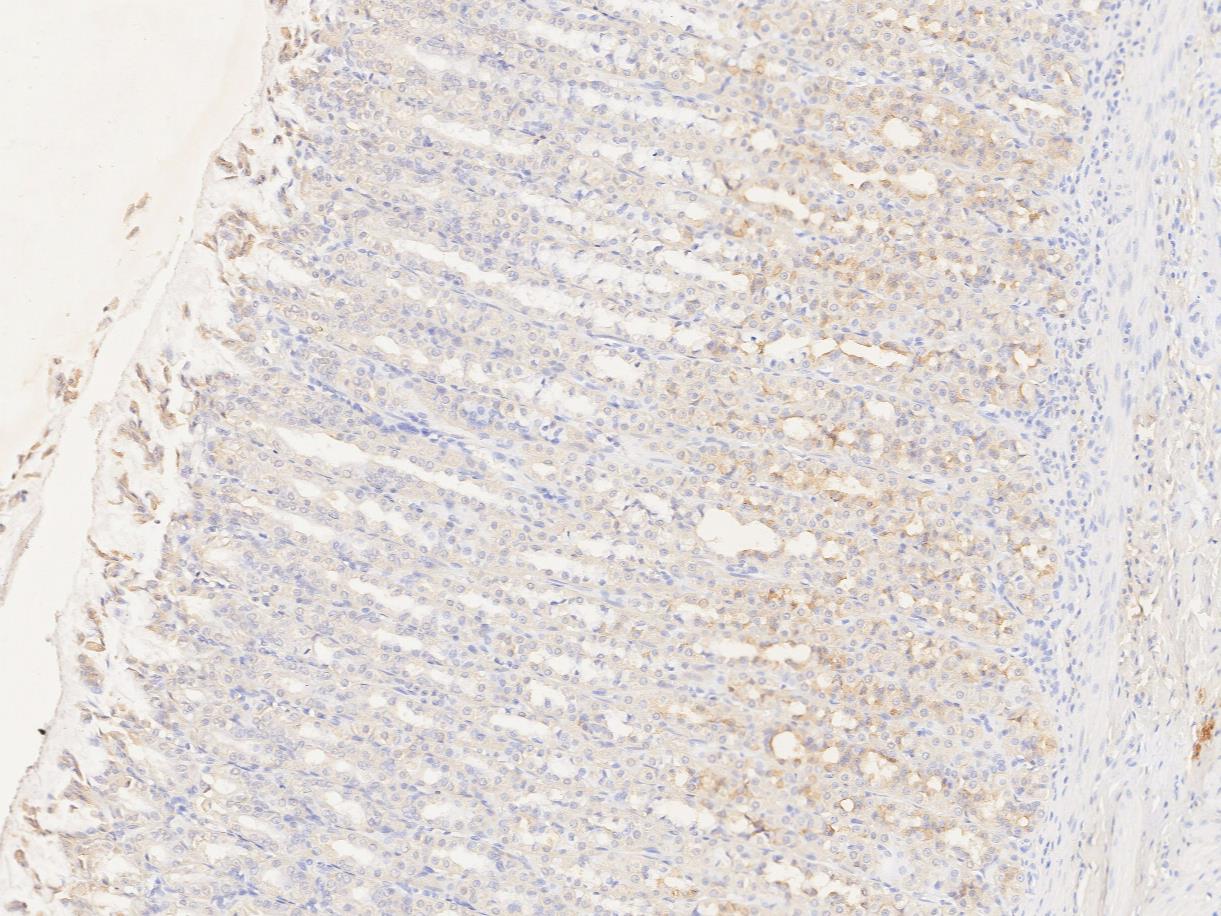

Supplement: Supplementary file 3 [file DataSheet1.zip › raw data/The images of Occludin, ZO-1, Claudin-4 and E-cadherin/ZO-1_M1.jpg]

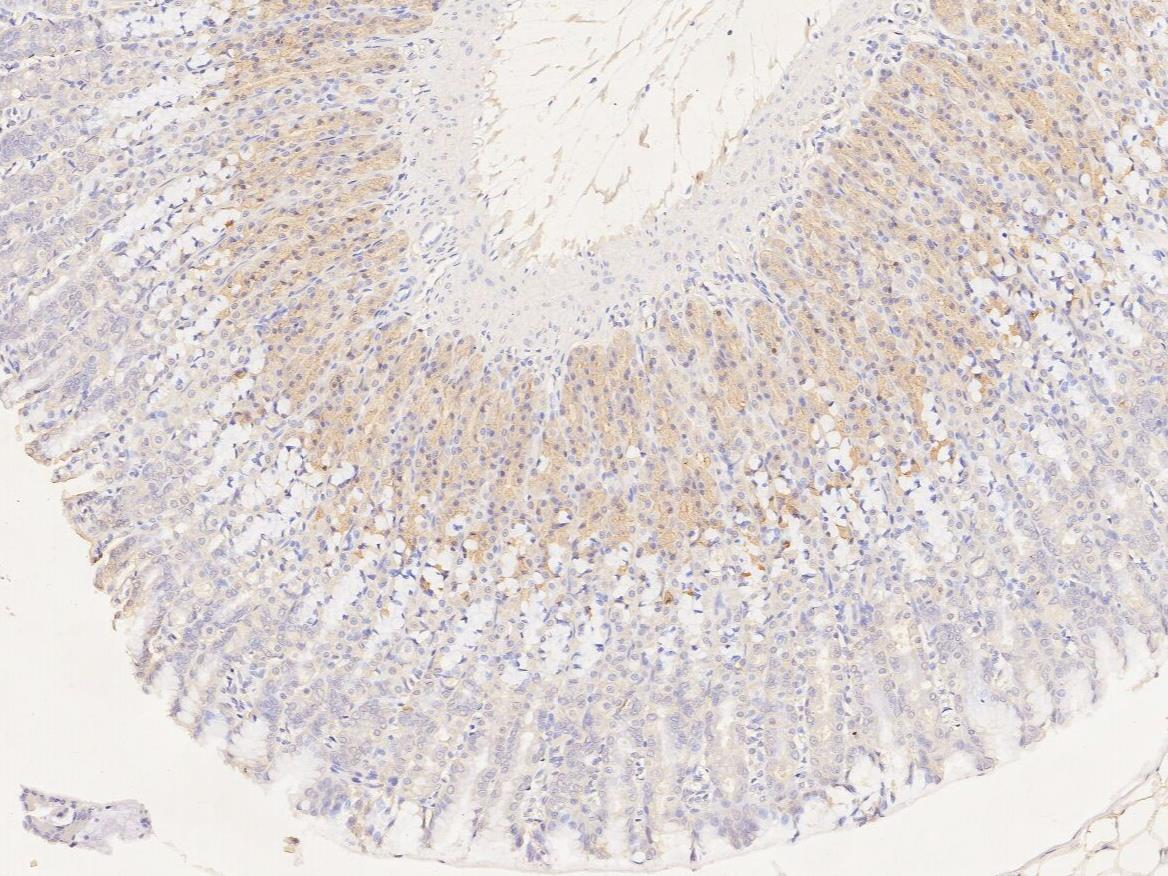

Supplement: Supplementary file 3 [file DataSheet1.zip › raw data/The images of Occludin, ZO-1, Claudin-4 and E-cadherin/ZO-1_M2.jpg]

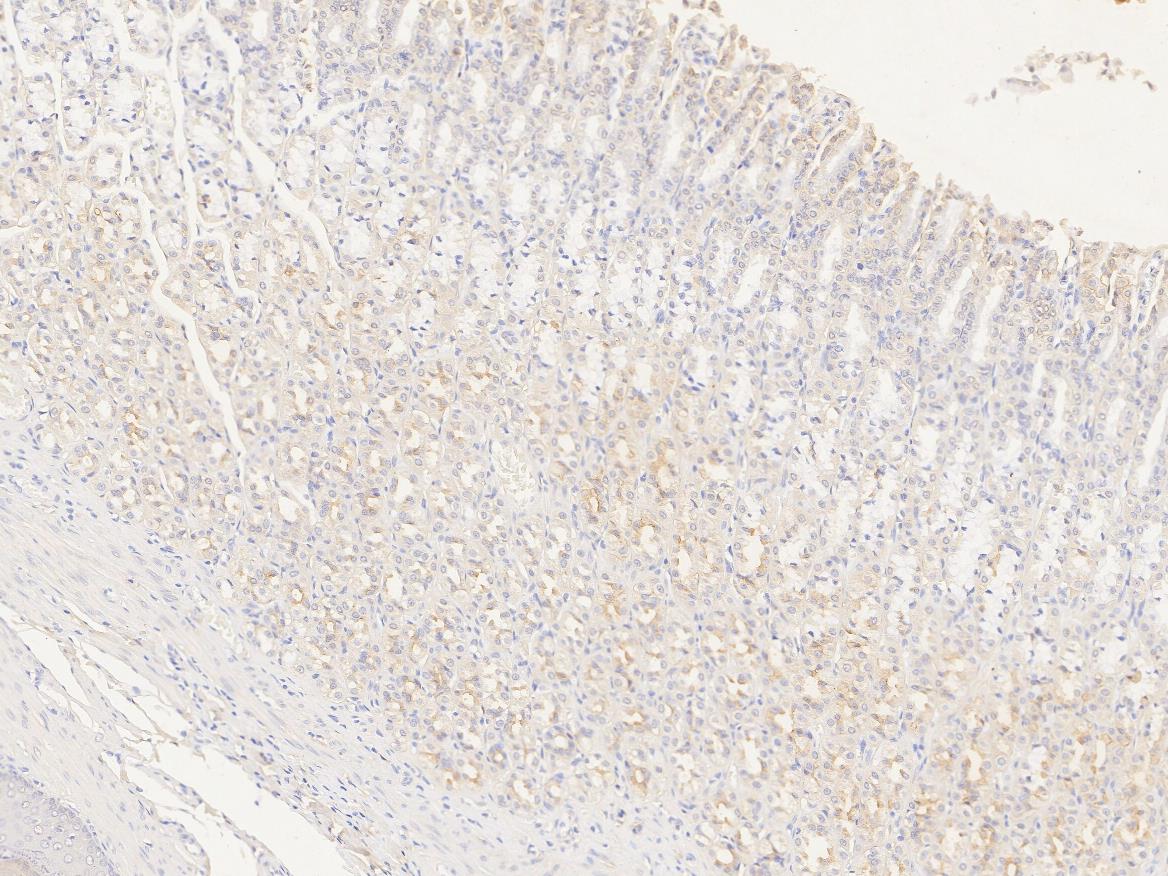

Supplement: Supplementary file 3 [file DataSheet1.zip › raw data/The images of Occludin, ZO-1, Claudin-4 and E-cadherin/ZO-1_M3.jpg]

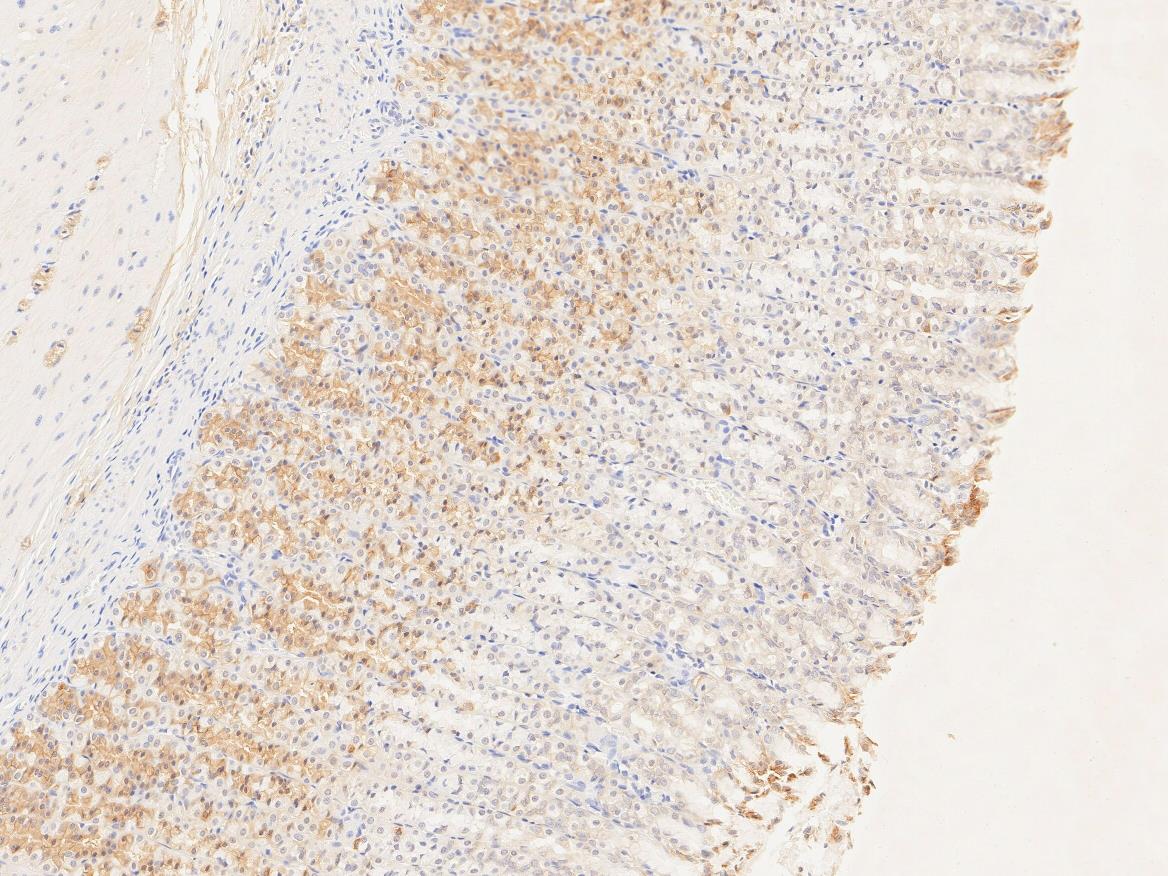

Supplement: Supplementary file 3 [file DataSheet1.zip › raw data/The images of Occludin, ZO-1, Claudin-4 and E-cadherin/cloudin4_BH1.jpg]

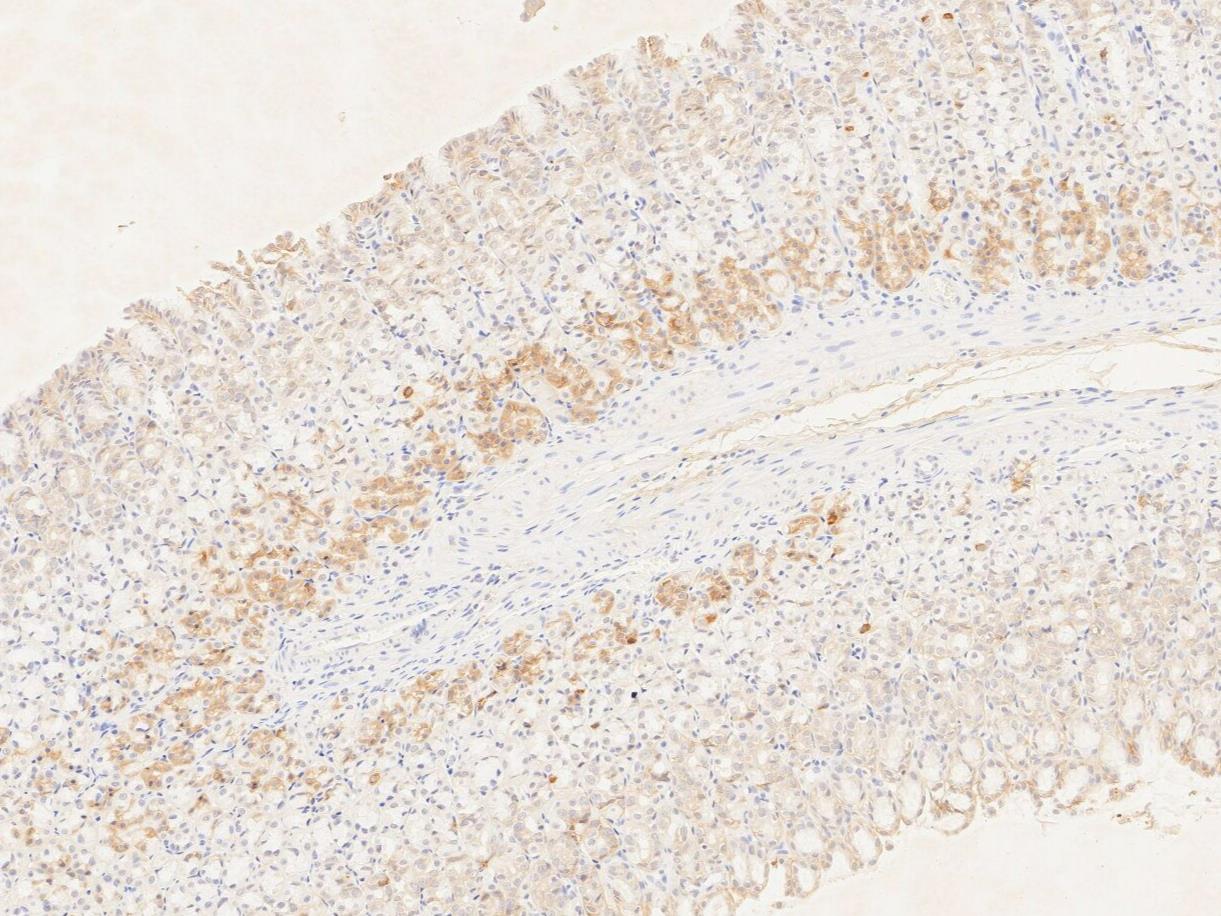

Supplement: Supplementary file 3 [file DataSheet1.zip › raw data/The images of Occludin, ZO-1, Claudin-4 and E-cadherin/cloudin4_BH2.jpg]

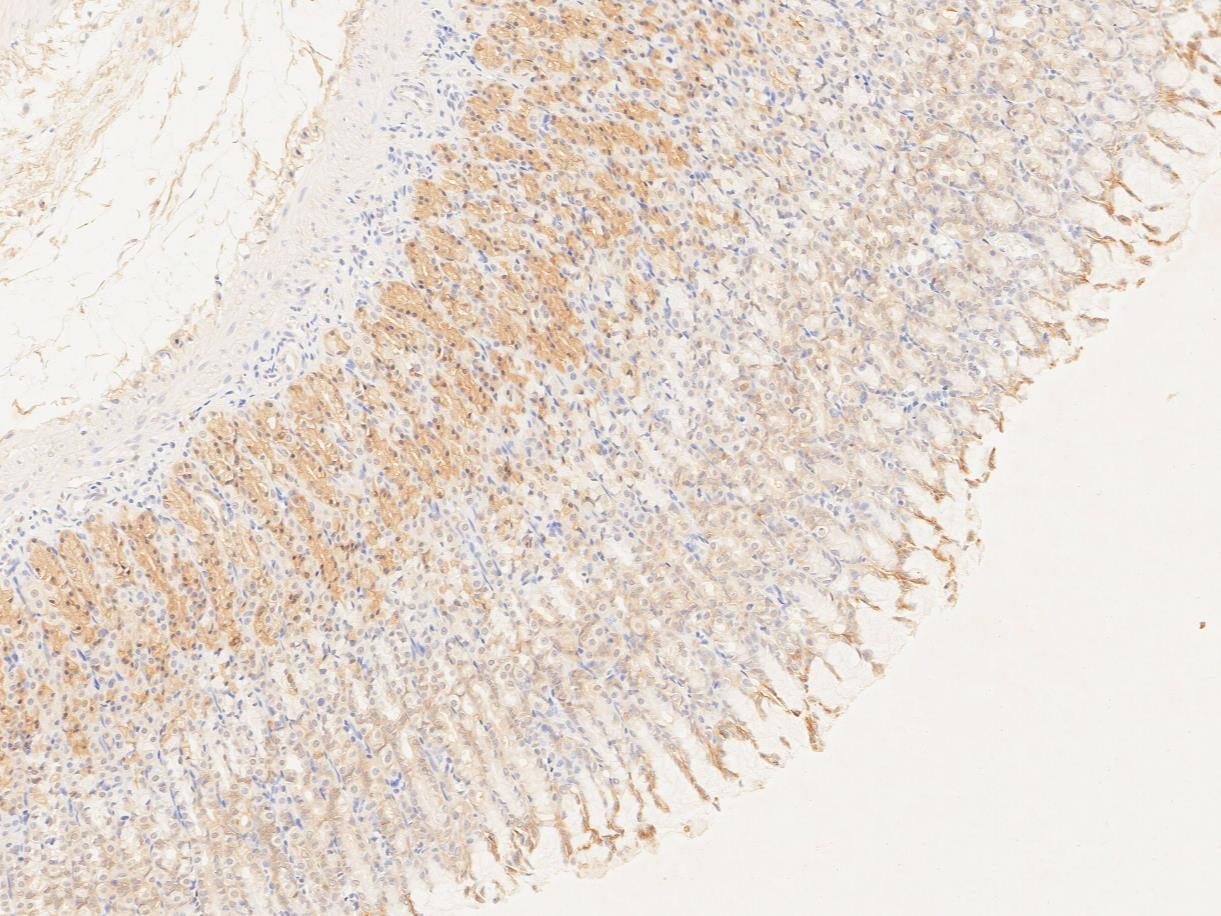

Supplement: Supplementary file 3 [file DataSheet1.zip › raw data/The images of Occludin, ZO-1, Claudin-4 and E-cadherin/cloudin4_BH3.jpg]

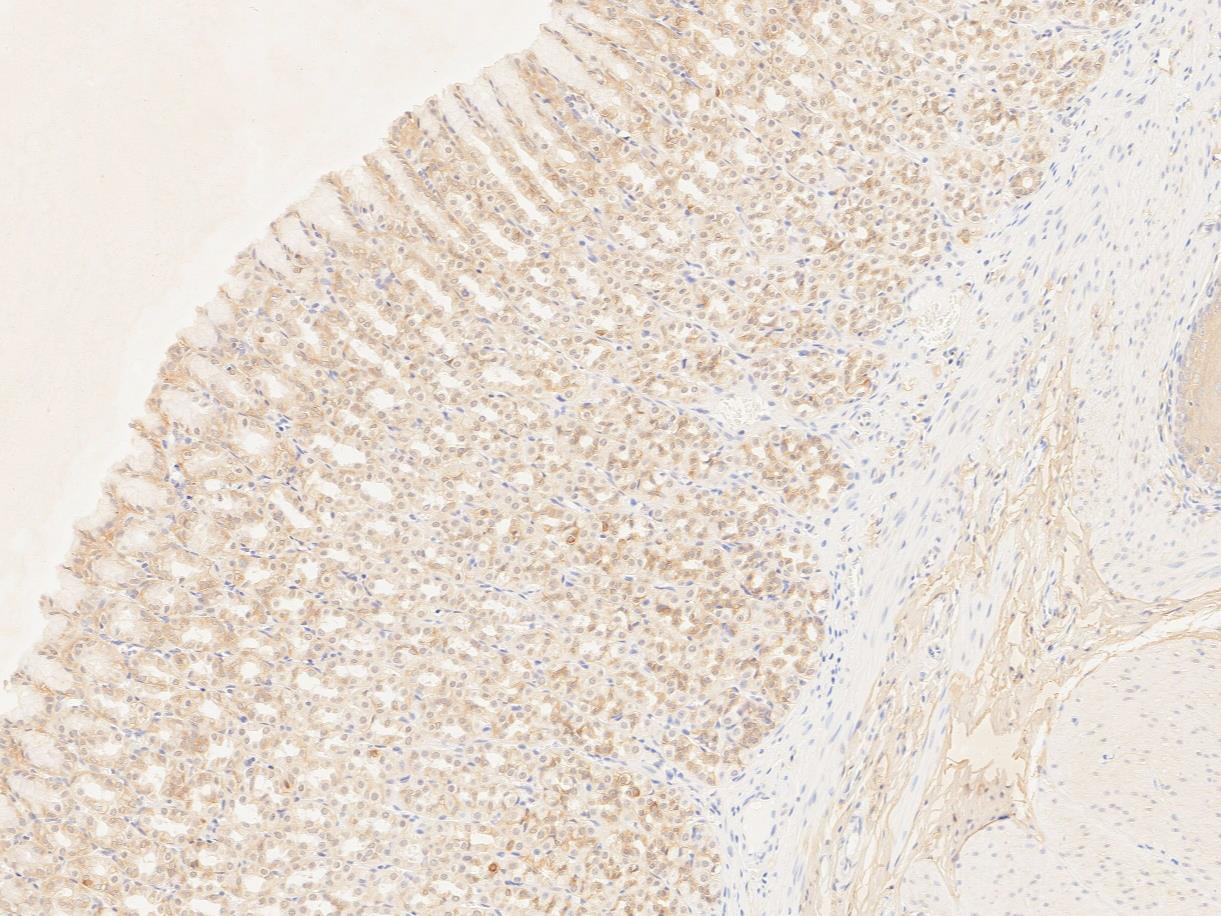

Supplement: Supplementary file 3 [file DataSheet1.zip › raw data/The images of Occludin, ZO-1, Claudin-4 and E-cadherin/cloudin4_BL1.jpg]

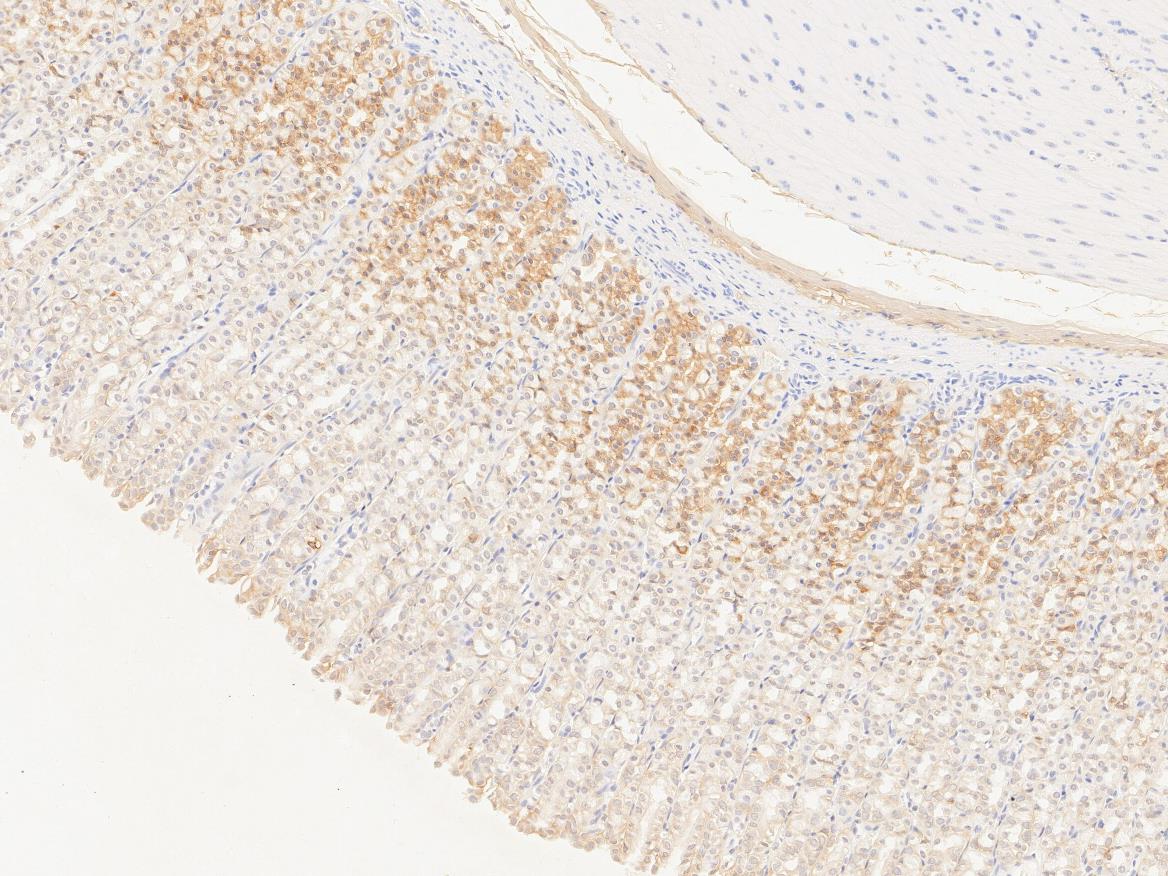

Supplement: Supplementary file 3 [file DataSheet1.zip › raw data/The images of Occludin, ZO-1, Claudin-4 and E-cadherin/cloudin4_BL2.jpg]

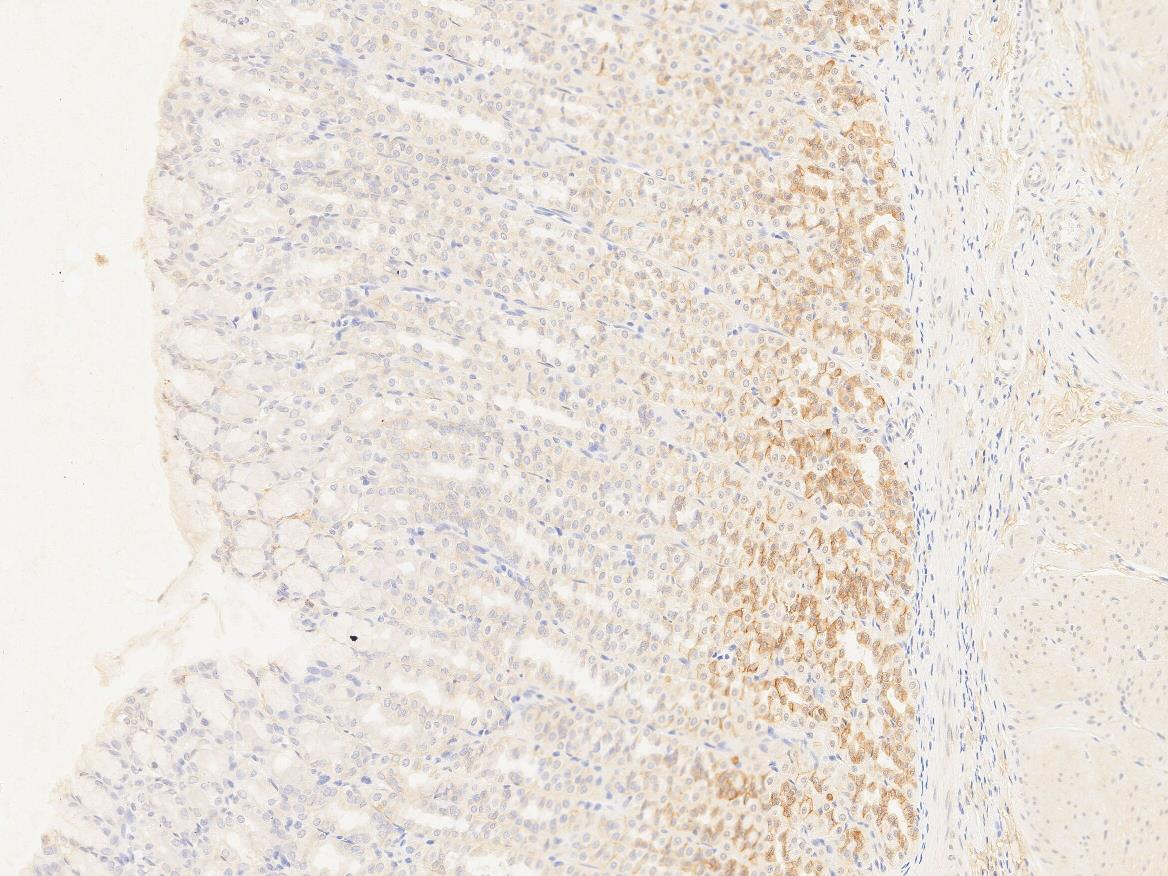

Supplement: Supplementary file 3 [file DataSheet1.zip › raw data/The images of Occludin, ZO-1, Claudin-4 and E-cadherin/cloudin4_BL3.jpg]

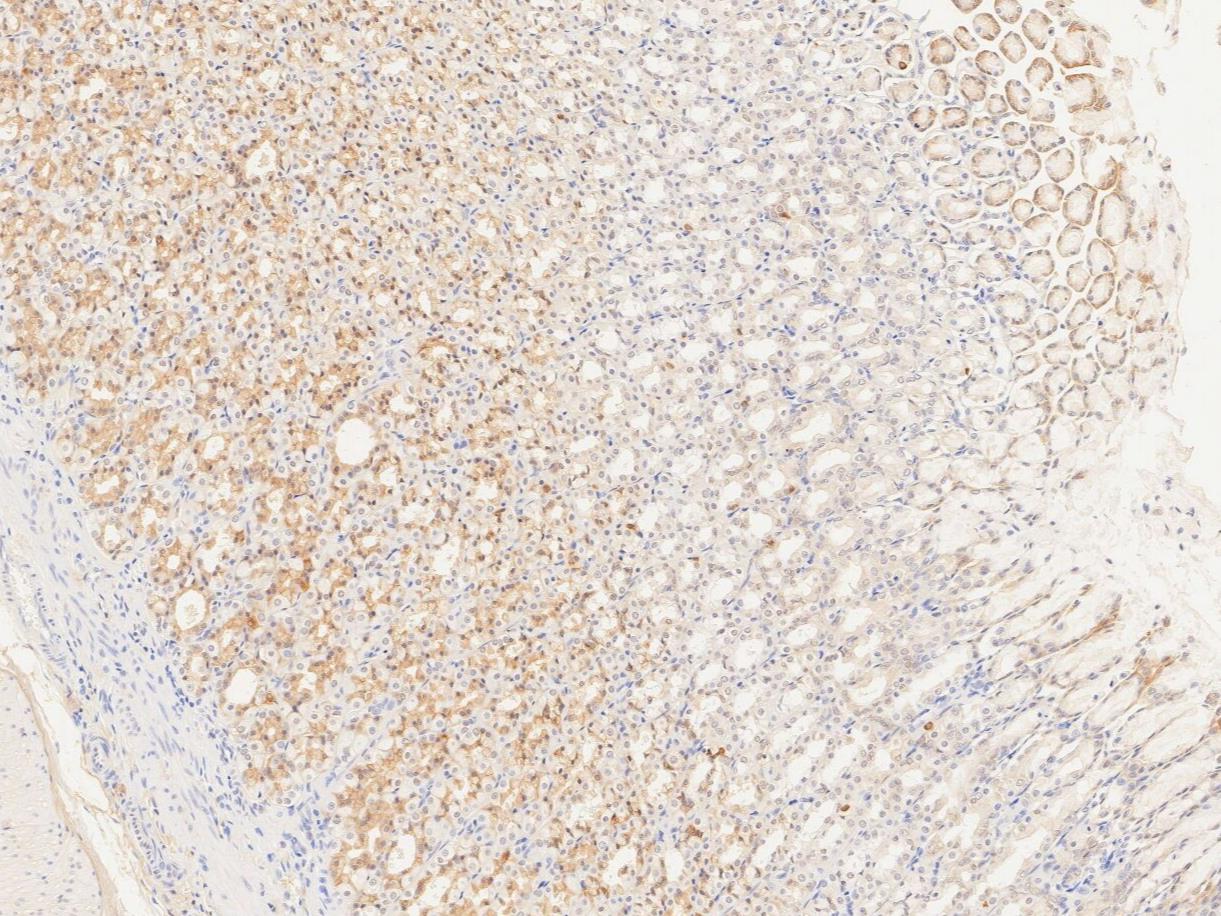

Supplement: Supplementary file 3 [file DataSheet1.zip › raw data/The images of Occludin, ZO-1, Claudin-4 and E-cadherin/cloudin4_K1.jpg]

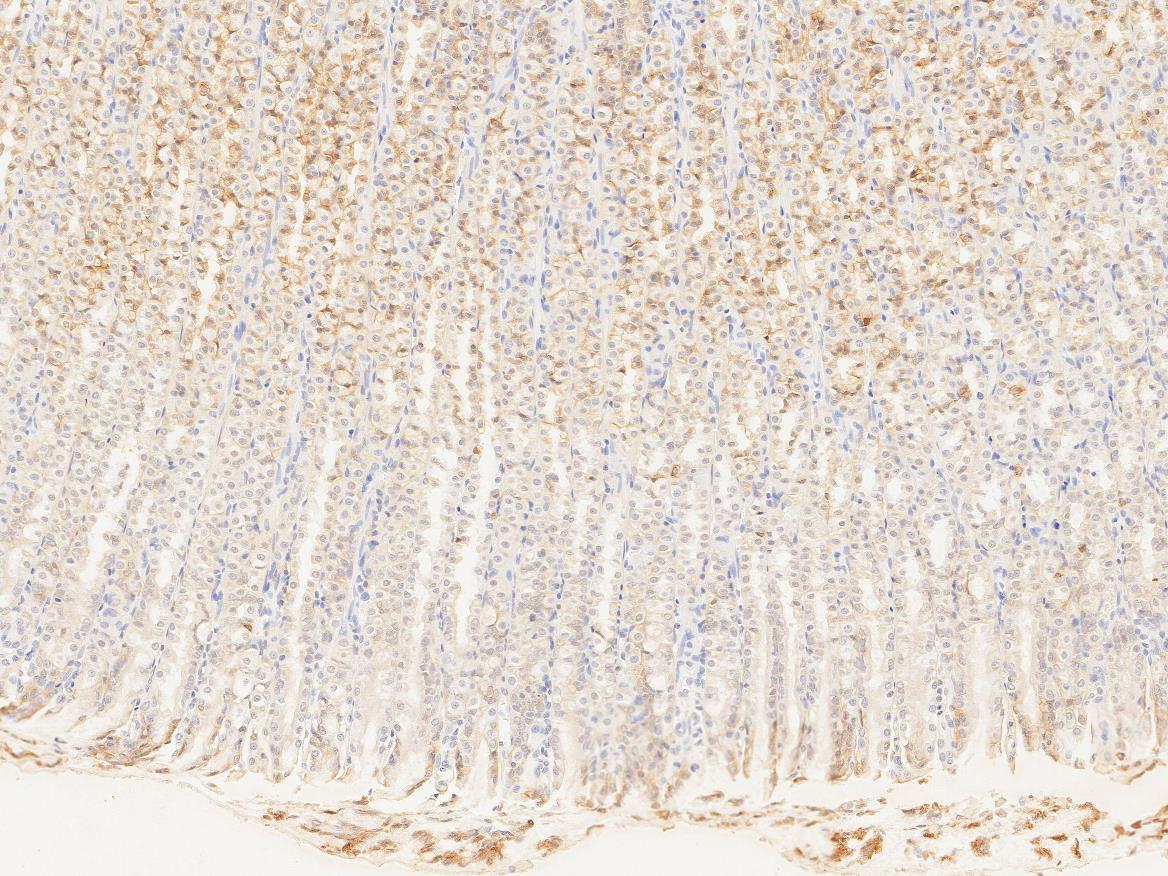

Supplement: Supplementary file 3 [file DataSheet1.zip › raw data/The images of Occludin, ZO-1, Claudin-4 and E-cadherin/cloudin4_K2.jpg]

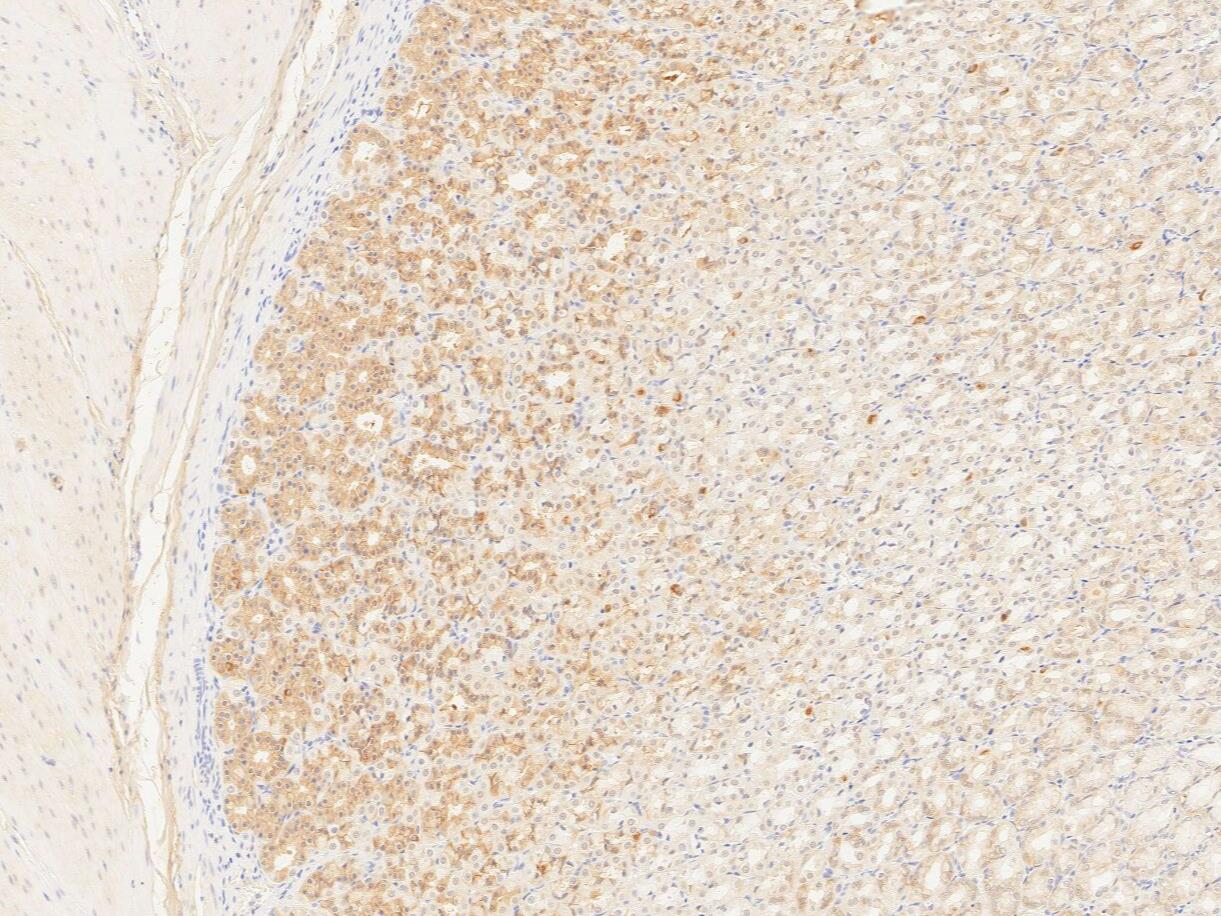

Supplement: Supplementary file 3 [file DataSheet1.zip › raw data/The images of Occludin, ZO-1, Claudin-4 and E-cadherin/cloudin4_K3.jpg]

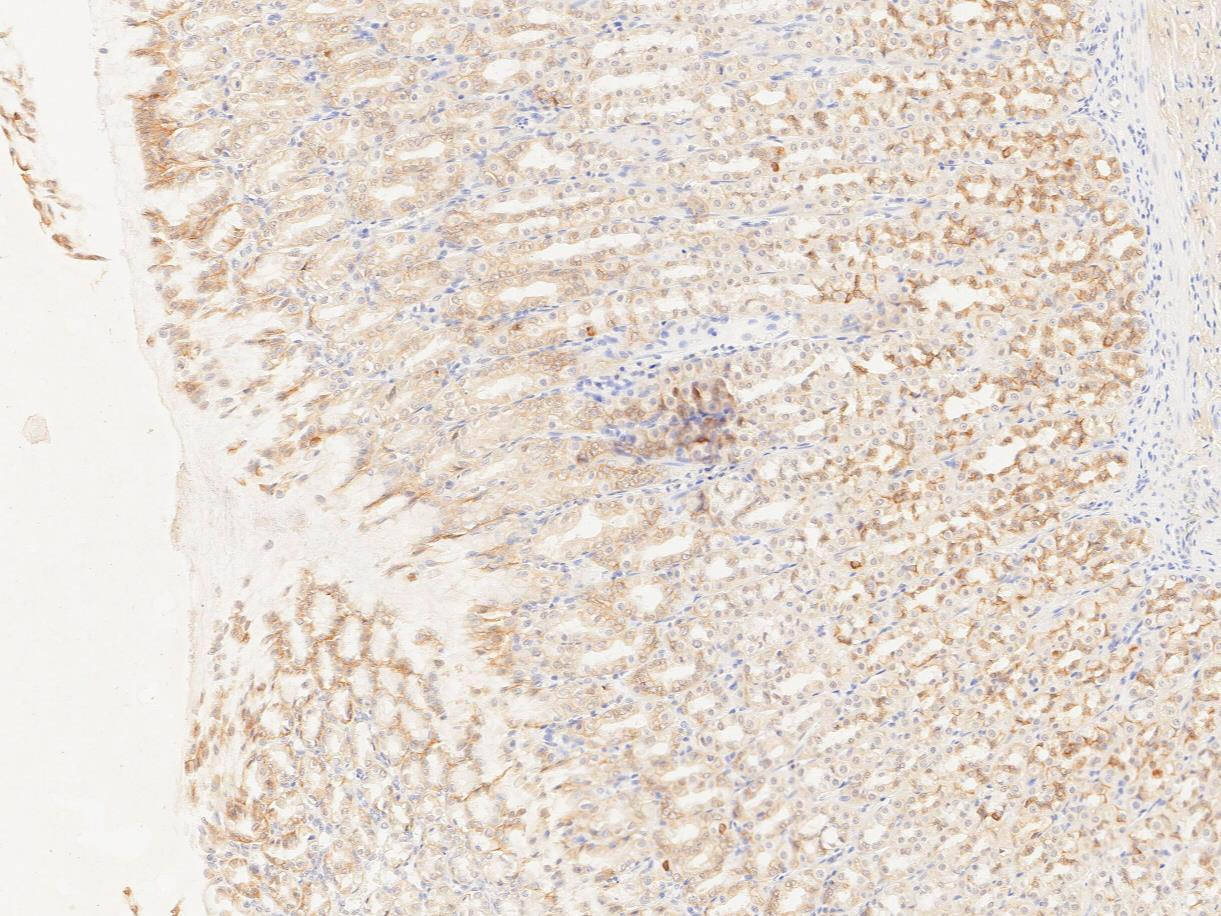

Supplement: Supplementary file 3 [file DataSheet1.zip › raw data/The images of Occludin, ZO-1, Claudin-4 and E-cadherin/cloudin4_M1.jpg]

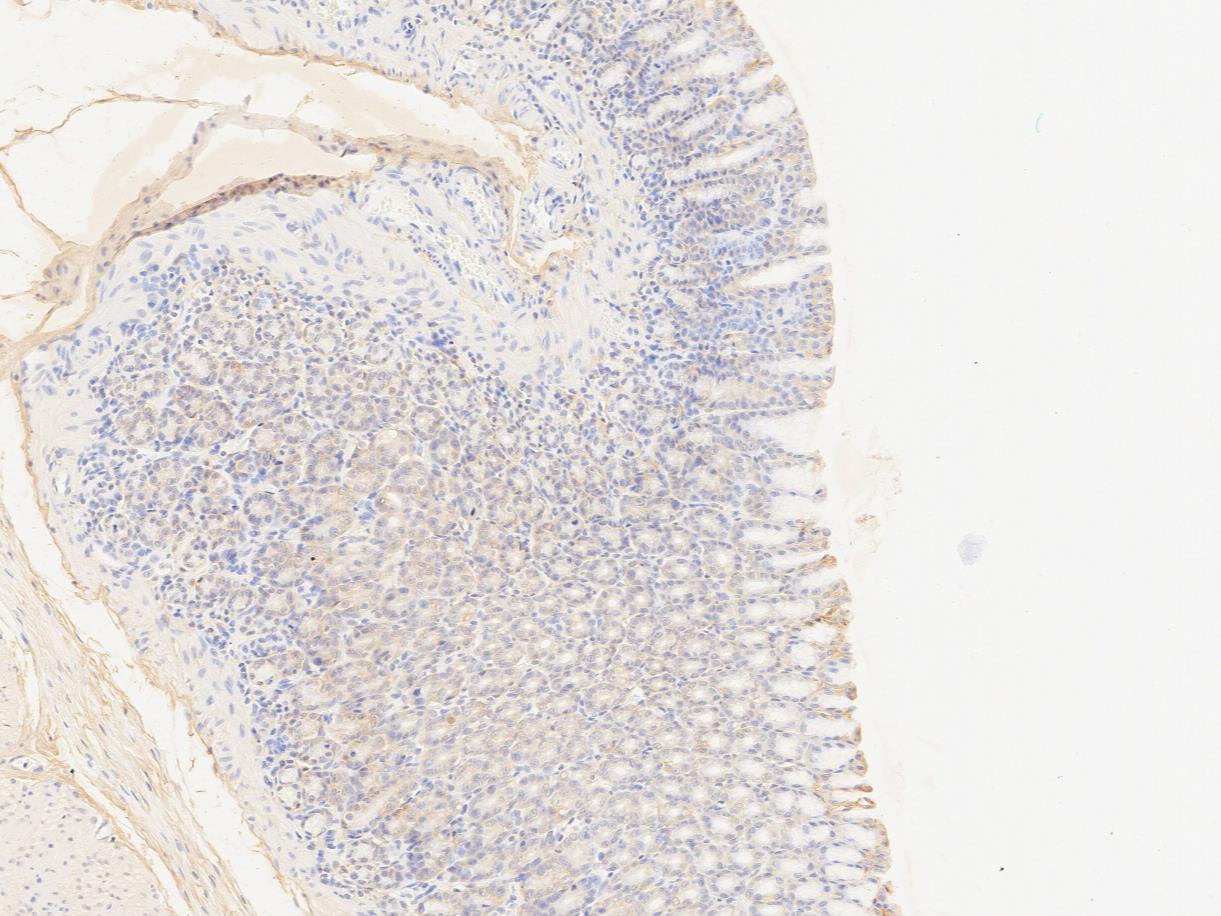

Supplement: Supplementary file 3 [file DataSheet1.zip › raw data/The images of Occludin, ZO-1, Claudin-4 and E-cadherin/cloudin4_M2.jpg]

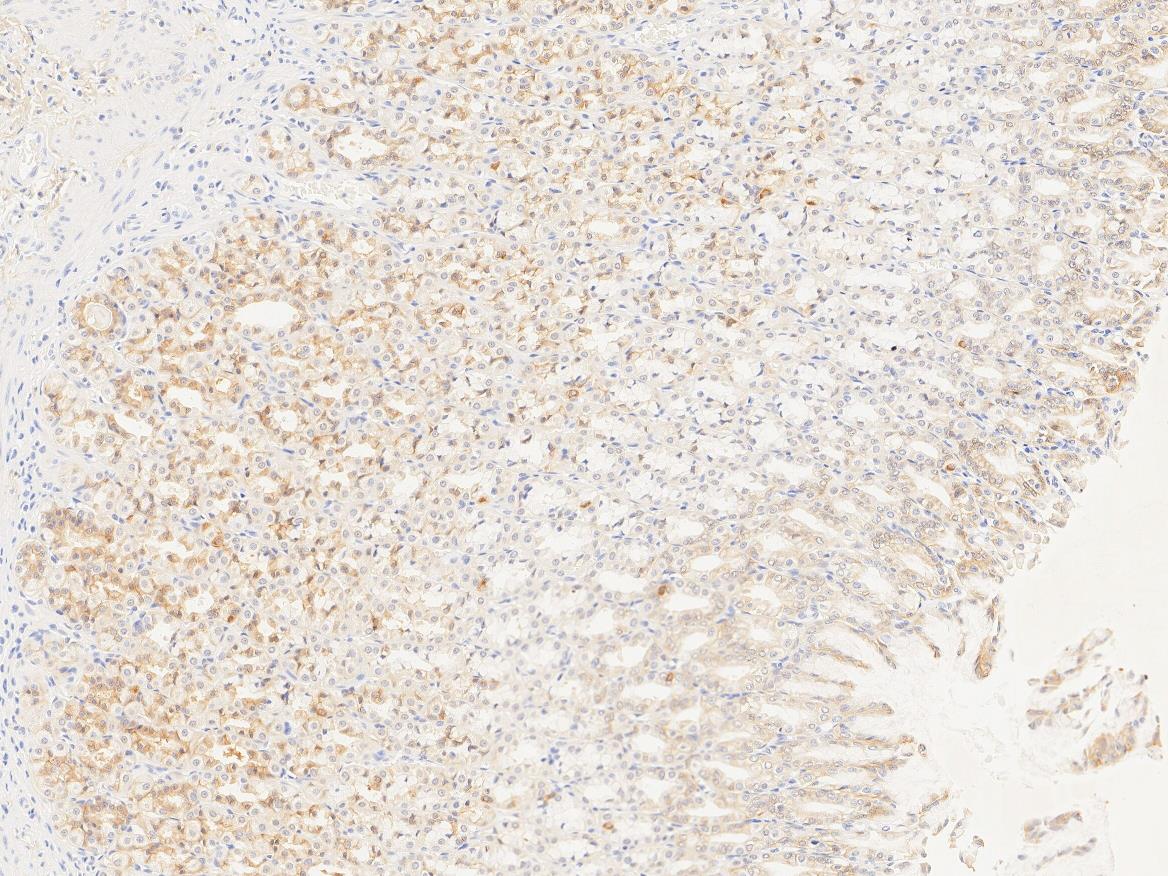

Supplement: Supplementary file 3 [file DataSheet1.zip › raw data/The images of Occludin, ZO-1, Claudin-4 and E-cadherin/cloudin4_M3.jpg]

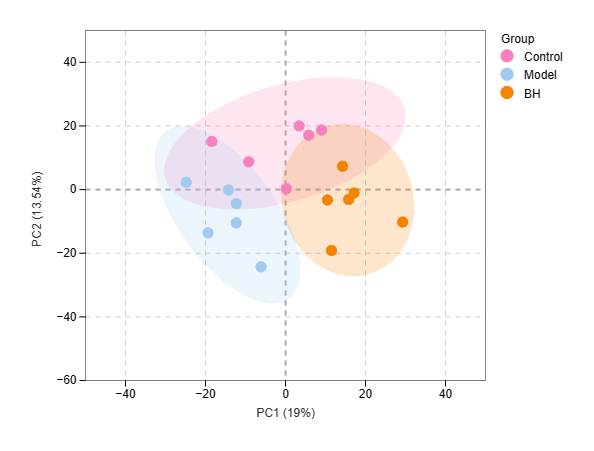

Supplement: Supplementary file 4 [file Image2.jpeg]

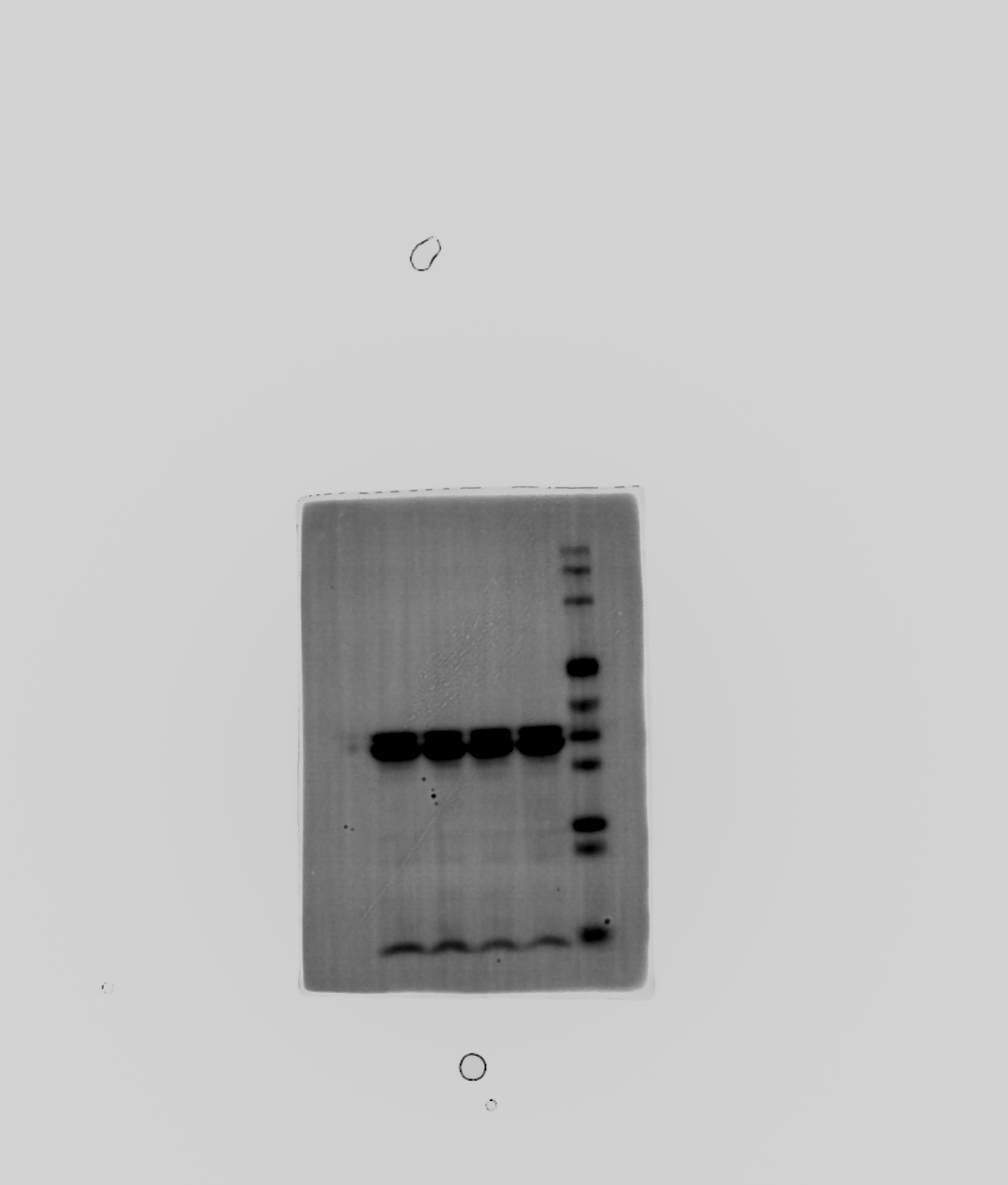

Supplement: Supplementary file 8 [file DataSheet2.zip › Original Images for Blots/Original Images for Blots/ERK-20240127163920T1.tif]

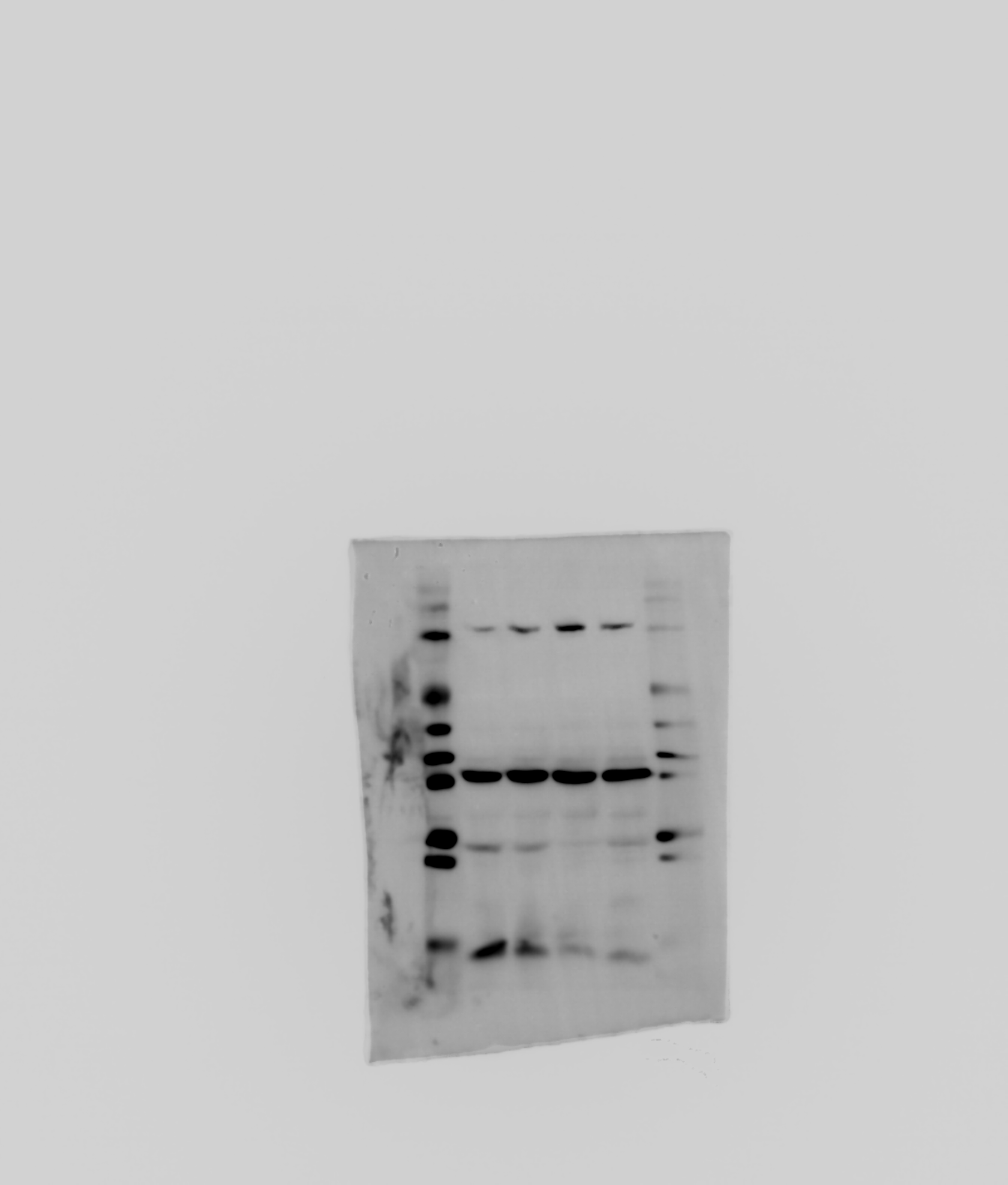

Supplement: Supplementary file 8 [file DataSheet2.zip › Original Images for Blots/Original Images for Blots/GAPDH-20240123054818T0.1.tif]

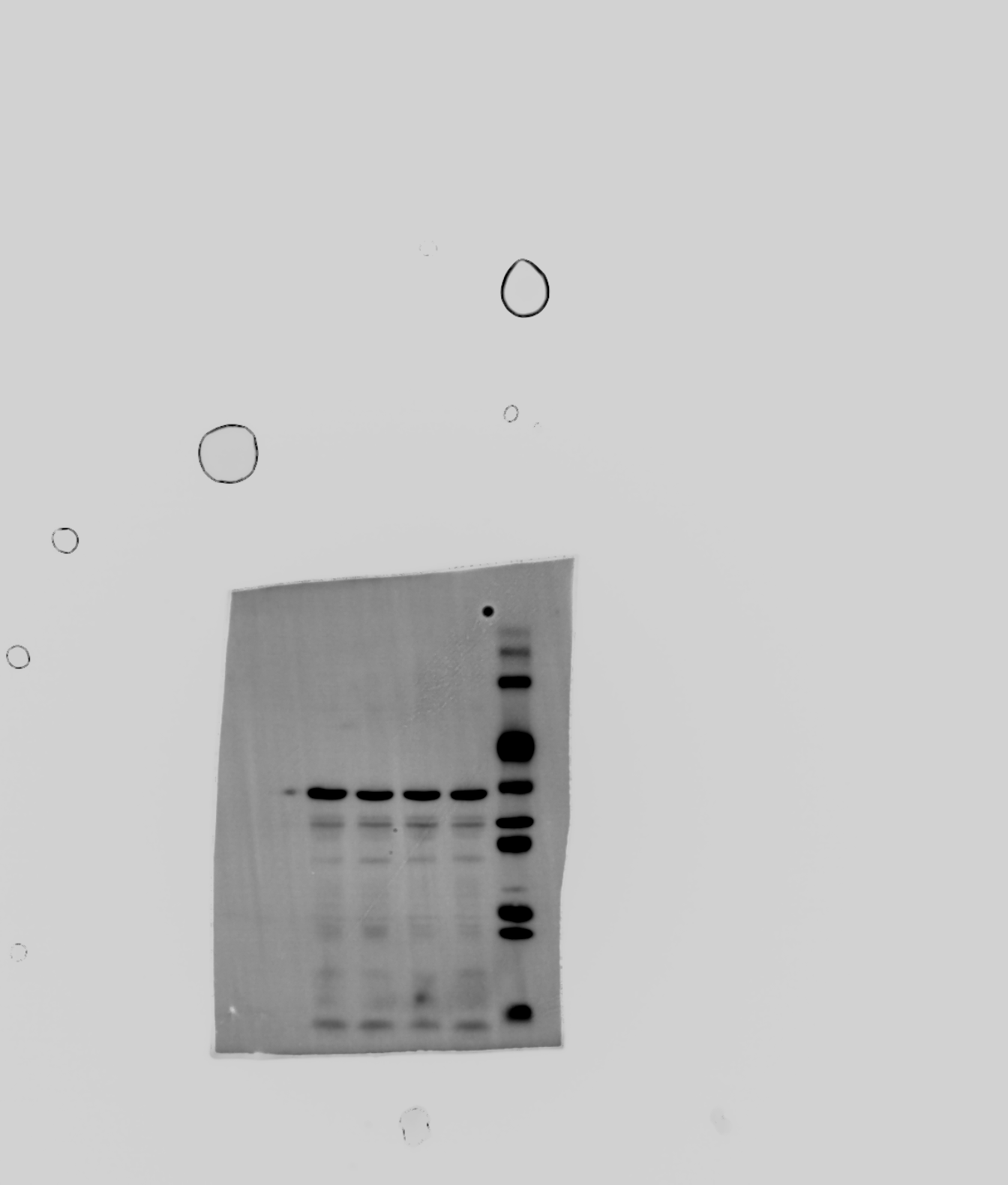

Supplement: Supplementary file 8 [file DataSheet2.zip › Original Images for Blots/Original Images for Blots/JNK-20240127164833T1.tif]

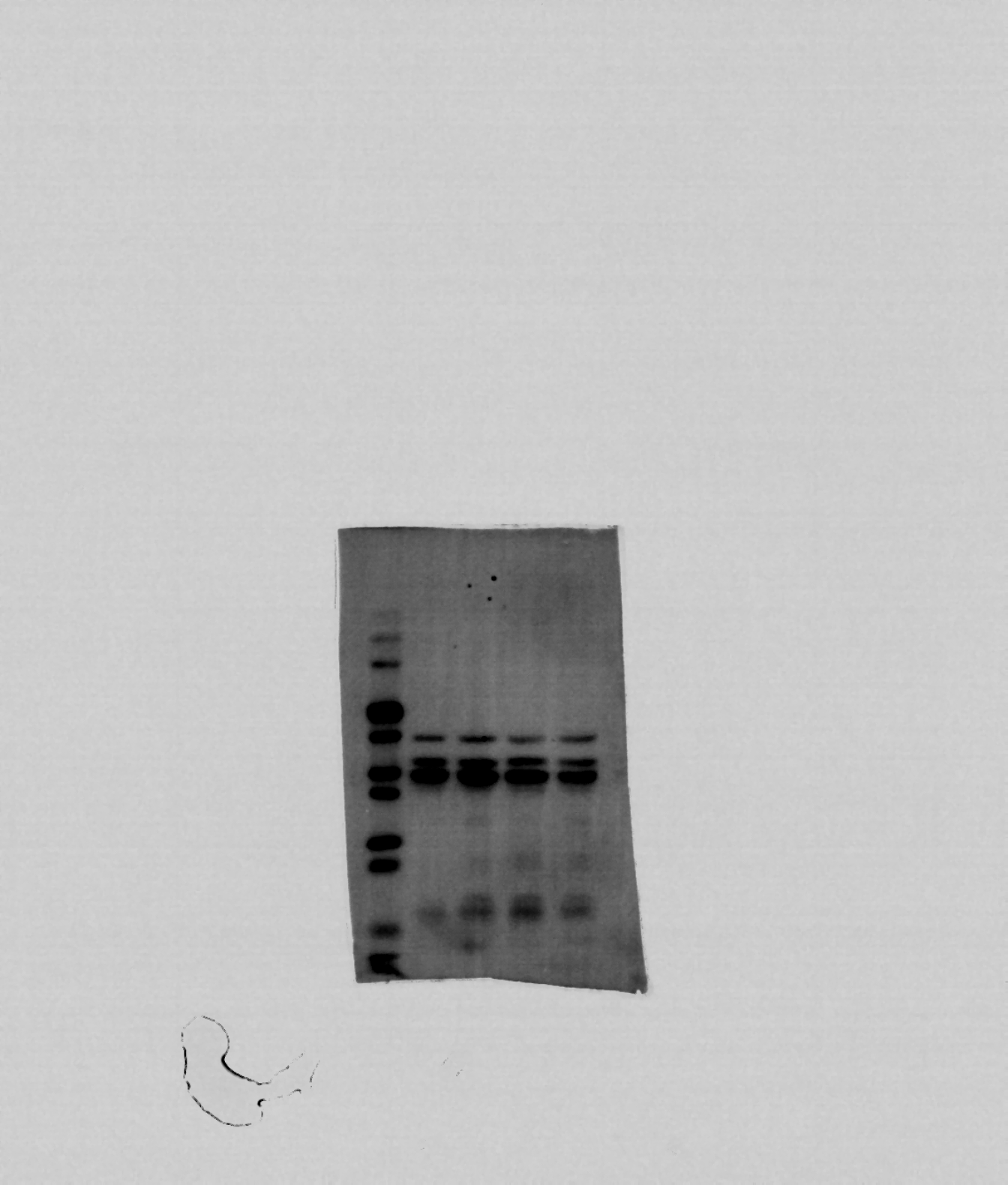

Supplement: Supplementary file 8 [file DataSheet2.zip › Original Images for Blots/Original Images for Blots/P-ERK-20240131215121T0.5.tif]

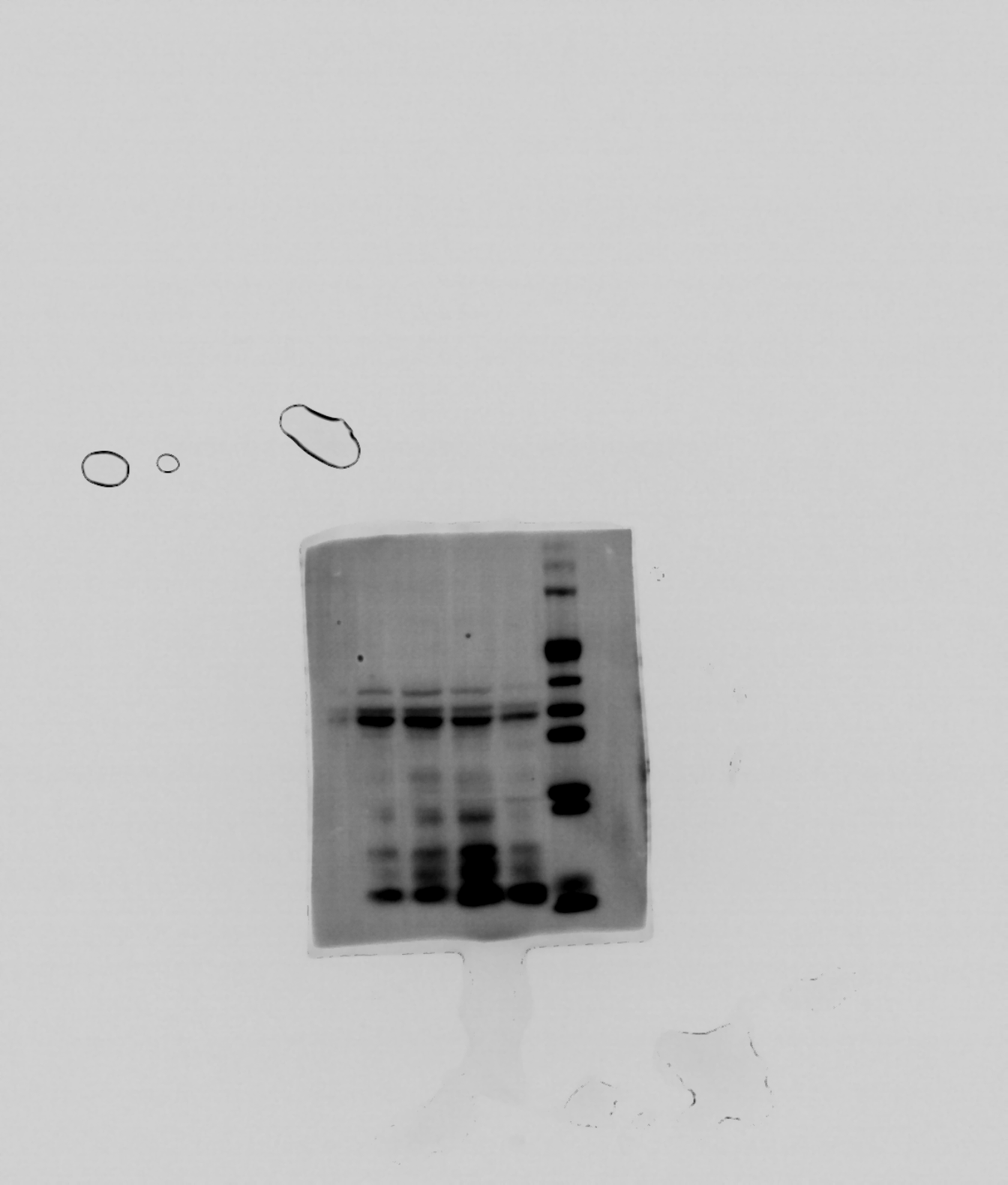

Supplement: Supplementary file 8 [file DataSheet2.zip › Original Images for Blots/Original Images for Blots/P-JNK-20240131213932T0.5.tif]

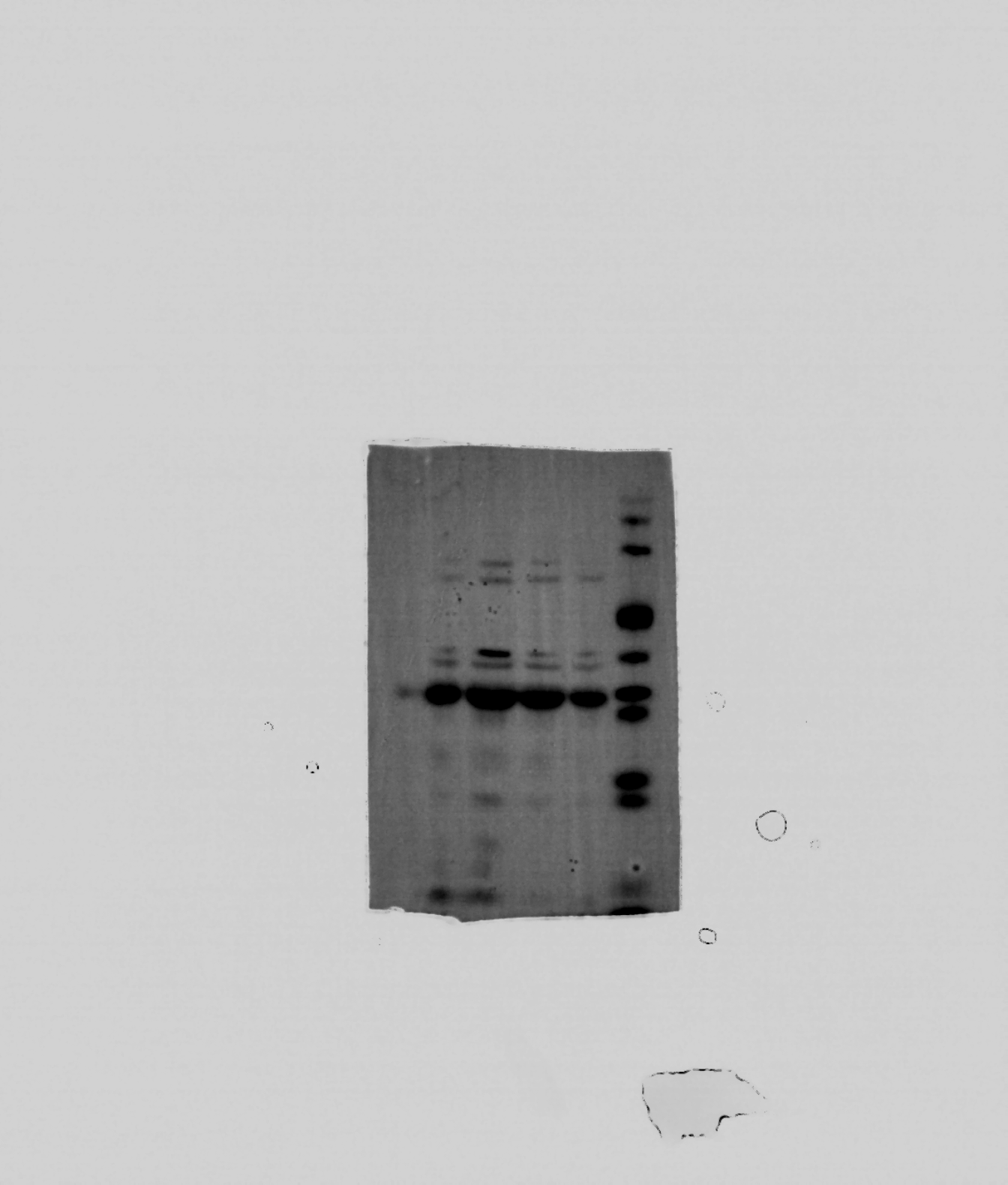

Supplement: Supplementary file 8 [file DataSheet2.zip › Original Images for Blots/Original Images for Blots/P-P38-20240129220239T0.1.tif]

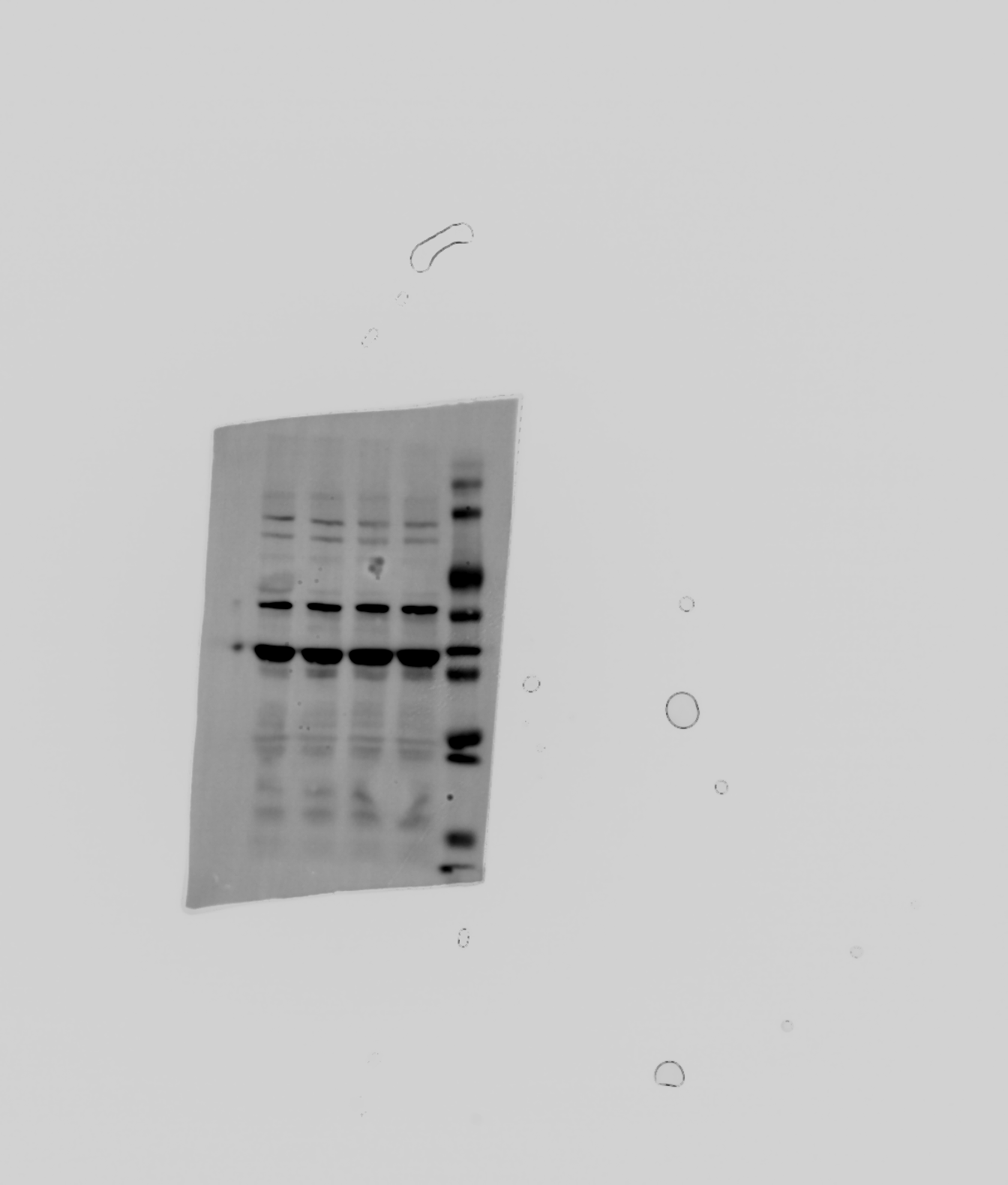

Supplement: Supplementary file 8 [file DataSheet2.zip › Original Images for Blots/Original Images for Blots/P38-20240127163049T0.1.tif]
