# Supplementary material for: Elucidation of the mechanism of berberine against gastric mucosa injury in a rat model with chronic atrophic gastritis based on a combined strategy of multi-omics and molecular biology
Source: Front Pharmacol. 2025 Jan 6;15:1499753. doi: 10.3389/fphar.2024.1499753 (PMC11743660; doi:10.3389/fphar.2024.1499753)
Supplement: Supplementary file 5 [file Table2.docx]

**Supplementary Table 2. Primers used for real-time PCR**

| **Gene** | **Forward (5′–3′)** | **Reverse (5′–3′)** |
| --- | --- | --- |
| **ITGB1** | CTGTGAATGTGGTGCTTGTAAGTG | AAGGCAGGTCTGACAGGTCTC |
| **TNC** | CCTGGTCTATGAGTCTGTGGATG | TGCCTGGATTCTTGCTGTGTAG |
| **FN1** | CTGTGAAGAACGAGGAGGATGTG | CACGCTGGAGACACTGACTAAG |
| **COL8A2** | GTGGGAGGCGTCTACTACTTTG | CTTCTTGTATTCGTCGTATGTGTAGG |
| **COL8A1** | ACACATACGACGAGTACAAGAAGG | TCTGAAGGCATTTGGAGGAACAC |
| **LGALS1** | ACAACCTGTGCCTACACTTCAAC | GTCCCATCGTCCTTGCTGTTAC |
| **RPS6KA2** | ATCGGGACCTGAAACCAAGTAAC | AGTCACAGATGCGGATAGATTCG |
| **PHLDA3** | CGCCACATCTACTTCACGCTAG | GCCTGTTGATTCTTGAACTTGACC |
| **NOG** | GCCAGCACTATCTACACATCCG | GTCTCGTTCAGATCCTTCTCCTTAG |
| **SLIT2** | GAATGTGAGGAAGGGTGGATGG | CTGTAGGAGAAGGCGTTGATGG |
| **CX3CL1** | ACAAGATGACCTCGCCAATCC | GTGTCTGTGCTGTCTCGTCTC |
| **HADHA** | GGTGTCTTGCTCCCATGATGTCAG | GAAGCCGAAGCCTGTGGTCAAG |
| **CPT1A** | CAGGAGAGTGCCAGGAGGTCATAG | TGCCGAAAGAGTCAAATGGGAAGG |
| **ACAA2** | GCTCCTCAGTTCTTGGCTGTTCAG | CAGGTGTGCGGTGATTCTGGATC |
| **CTPS1** | CCTGTAGACGAAGATGGCTTAGAAC | CTTGAACTGGAACTGGCGGAAG |
| **GFPT2** | ACACGGAGACCATCGCCAAG | GCCTTCCAACTGCTGAATGACTC |
| **ACOT2** | ACTTTGAAGAAGCCGTGAACTACC | ATGACAACAGCAGCCGTGATG |
| **ACOT4** | ATGACGCTGTAGGAGGCTGTG | AACCGTTCCGAGGCTATCTGAG |
| **ACSL1** | CTCAGAGCAGTTCATCGGCATC | GTCGGTTCCAAGCGTGTCATAG |
| **GCLM** | AAGTTAATCTTGCCTCCTGCTGTG | TTTGGGTCATTGTGAGTCAGTAGC |
| **GSR** | GGATTGGCTGCGATGAGATGC | GTAGGATGAATGGCGACCGTATTG |
| **PLA2G4C** | CTGTTGGATGCTGTCACATACCTC | GTGCCACTCATTCTTCTCATACCG |
| **PNLIPRP1** | GAGGAGCGGACGGAGTACAAC | CTGAGGTTTCACAAGGCAAGAGAG |
| **SLC25A20** | TGGTGGCTGGCGGCTTTG | GGAGGTGCGGTCTGGAATCG |
| **GAPDH** | AAGTTCAACGGCACAGTCAAGG | GACATACTCAGCACCAGCATCAC |
