# Supplementary material for: Elucidation of the mechanism of berberine against gastric mucosa injury in a rat model with chronic atrophic gastritis based on a combined strategy of multi-omics and molecular biology
Source: Front Pharmacol. 2025 Jan 6;15:1499753. doi: 10.3389/fphar.2024.1499753 (PMC11743660; doi:10.3389/fphar.2024.1499753)
Supplement: Supplementary file 7 [file Table3.docx]

**Supplementary Table 3. The specific information of the antibodies**

| **Antibodies** | **Dilution** | **Manufacturer** | **Cat. No.** |
| --- | --- | --- | --- |
| Rabbit Anti-ERK | 1:1000 | BOSTER | BM4326 |
| Rabbit Anti-JNK | 1:1000 | BOSTER | BA1219 |
| Rabbit Anti-P38 | 1:1000 | BOSTER | A00176-2 |
| Rabbit Anti-p-ERK | 1:1000 | Cell Signaling Technology | 4668 |
| Rabbit Anti-p-JNK | 1:1000 | Cell Signaling Technology | 4370 |
| Rabbit Anti-p-P38 | 1:1000 | Cell Signaling Technology | 4511 |
| Rabbit Anti-GAPDH | 1:1000 | Huaxingbio | HX1832 |
| Anti-Rabbit IgG (H + L)/HRP | 1:10000 | Huaxingbio | HX2031 |
